# Group-based nutrition interventions to promote healthy eating and mobility in community-dwelling older adults: a systematic review

**DOI:** 10.1017/S136898002200115X

**Published:** 2022-05-16

**Authors:** Kylie Teggart, Rebecca Ganann, Davneet Sihota, Caroline Moore, Heather Keller, Christine Senson, Stuart M Phillips, Sarah E Neil-Sztramko

**Affiliations:** 1 School of Nursing, Faculty of Health Sciences, McMaster University, Hamilton, ON, Canada; 2 Global Health Graduate Program, Faculty of Health Sciences, McMaster University, Hamilton, ON, Canada; 3 Schlegel-UW Research Institute for Aging, Waterloo, ON, Canada; 4 Department of Kinesiology and Health Sciences, Faculty of Health, University of Waterloo, Waterloo, ON, Canada; 5 Healthy and Safe Communities Department, City of Hamilton Public Health Services, Hamilton, ON, Canada; 6 Department of Kinesiology, Faculty of Science, McMaster University, Hamilton, ON, Canada; 7 Department of Health Research Methods, Evidence and Impact, Faculty of Health Sciences, McMaster University, Hamilton, ON L8S 4L8, Canada

**Keywords:** Older adults, Nutrition, Healthy eating, Systematic review, Community

## Abstract

**Objective:**

To identify the efficacy of group-based nutrition interventions to increase healthy eating, reduce nutrition risk, improve nutritional status and improve physical mobility among community-dwelling older adults.

**Design:**

Systematic review. Electronic databases MEDLINE, CINAHL, EMBASE, PsycINFO and Sociological Abstracts were searched on July 15, 2020 for studies published in English since January 2010. Study selection, critical appraisal (using the Joanna Briggs Institute’s tools) and data extraction were performed in duplicate by two independent reviewers.

**Setting:**

Nutrition interventions delivered to groups in community-based settings were eligible. Studies delivered in acute or long-term care settings were excluded.

**Participants:**

Community-dwelling older adults aged 55+ years. Studies targeting specific disease populations or promoting weight loss were excluded.

**Results:**

Thirty-one experimental and quasi-experimental studies with generally unclear to high risk of bias were included. Interventions included nutrition education with behaviour change techniques (BCT) (e.g. goal setting, interactive cooking demonstrations) (*n* 21), didactic nutrition education (*n* 4), interactive nutrition education (*n* 2), food access (*n* 2) and nutrition education with BCT and food access (*n* 2). Group-based nutrition education with BCT demonstrated the most promise in improving food and fluid intake, nutritional status and healthy eating knowledge compared with baseline or control. The impact on mobility outcomes was unclear.

**Conclusions:**

Group-based nutrition education with BCT demonstrated the most promise for improving healthy eating among community-dwelling older adults. Our findings should be interpreted with caution related to generally low certainty, unclear to high risk of bias and high heterogeneity across interventions and outcomes. Higher quality research in group-based nutrition education for older adults is needed.

Older adults are the fastest-growing age group, and the number of adults aged 65 years and older worldwide is expected to more than double from 727 million in 2020 to over 1·5 billion in 2050^(1)^. As the population ages, the prevalence of chronic diseases, multimorbidity and frailty will also increase^(1–3)^. Several modifiable risk factors are associated with an increased risk of disability and disease with aging, one of which is poor diet quality^(4,5)^. Unfortunately, many older adults do not meet current age-specific nutrition guidelines^(6,7)^ concerning both diet quality and quantity^(8,9)^. As individuals age, many decrease their total food intake^(10)^, in part due to reduced appetite, sensory impairment, hormonal imbalance and changes in the gastrointestinal tract and dentition^(11)^. Age-related changes in living situations, retirement, social isolation and loss of relationships can also negatively impact food intake and diet quality^(12,13)^. The intersection of financial, psychosocial, environmental, physical, cognitive, gender and cultural factors are known to influence eating behaviour^(13)^, food access^(14)^ and mobility^(15)^ among older adults.

The relationship between mobility (the ability to move oneself within the immediate environment and broader community^(15)^) and nutrition has been shown to be bidirectional in older adults. One’s mobility can impact food access (e.g. ability to transport oneself to locations with high-quality food sources)^(16)^ and may also be influenced by dietary quality. Reduced intake of both micronutrients and macronutrients may lead to sarcopaenia^(17–19)^, and the loss of muscle mass in aging may result in mobility limitations and impaired quality of life^(20)^. Proper nutrition also plays an important role in maintaining skeletal strength and preventing falls and chronic diseases among older adults^(20–22)^. Given this, promoting healthier eating and reducing nutrition risk is necessary to maintain and improve health and mobility among community-dwelling older adults. However, many older adults perceive functional decline as an inevitable part of ageing and may experience difficulties accessing available programmes and services^(23)^.

Group-based nutrition interventions, including education, interactive discussion and hands-on activities, have demonstrated benefits in supporting older adults to learn from each other’s knowledge and experiences, overcome psychosocial and environmental barriers to healthy eating, enhance motivation and promote dietary behavioural change^(24–26)^. Group-based interventions among older adults also foster a sense of group cohesion^(27)^, allowing individuals to feel acknowledged and form bonds with others who understand their experiences firsthand. Although many group-based nutrition interventions exist, some of which have been formally evaluated for effectiveness^(28,29)^, these interventions vary widely and optimal design features remain unclear.

In a previous umbrella review of systematic reviews to identify existing synthesised evidence regarding group-based physical activity and/or nutrition interventions for community-dwelling older adults, only nine reviews evaluated interventions with a nutrition component (namely protein supplementation combined with physical activity)^(30)^. No systematic reviews of group-based nutrition interventions alone were identified, and there was no benefit observed for the addition of protein supplementation with physical activity in this population. Further, none of the nutrition interventions evaluated at the review level extended beyond supplementation, highlighting a lack of synthesised evidence to identify the effectiveness of group-based interventions targeting healthy eating. This understanding is key to informing the development and implementation of evidence-informed, group-based community programmes to promote healthy eating and mobility among older adults.

To address this gap, our team initiated a systematic review of single studies focussed on group-based nutrition interventions targeting healthy eating in community-dwelling older adults. We specifically aimed to understand whether group-based interventions targeting healthy eating in community-dwelling older adults (≥ 55 years) improved access to nutrition, affected nutritional intake or changed markers of physical mobility.

## Methods

This systematic review was registered with PROSPERO (CRD42020205045). The reporting of this review is based on PRISMA guidelines^(31)^.

### Search strategy

The electronic databases MEDLINE, CINAHL, EMBASE, PsycINFO and Sociological Abstracts were searched on July 15, 2020, by a research librarian trained in building search strategies for systematic reviews (see online supplementary material, Supplemental Table 1–5). To focus on interventions germane to the current context and nutrition guidelines, database searches were limited to studies published from January 2010. Only English language studies were eligible due to the research team’s capacity. Reference lists of all identified systematic reviews were screened for potentially relevant and eligible studies; experts in the field were contacted to locate any additional studies not identified in our search.

### Study selection

Citations were uploaded into Covidence (Veritas Health Innovation Ltd., Melbourne, Australia), and duplicates were removed. Following a pilot test, titles and abstracts were screened in duplicate by two independent reviewers against predetermined eligibility criteria. Full texts of potentially relevant studies were retrieved and screened for eligibility in duplicate by two independent reviewers. Disagreements were resolved through discussion or with the input of a third reviewer.

### Eligibility criteria

#### Types of studies

This review included experimental and quasi-experimental study designs, including randomised controlled trials (RCT), non-RCT, before and after studies and interrupted time-series studies. Mixed methods studies with quantitative designs cited above were also included, although only quantitative data were extracted and analysed. Theses and dissertations were eligible; publication status was not a criteria for inclusion. Conference abstracts, reviews, observational designs and qualitative studies were excluded.

#### Participants

Eligible studies must have included community-dwelling older adults ≥ 55 years old or reported a mean age of participants as ≥ 55 years. Studies focussed on disease-specific populations were excluded, although included participants could report risk factors for or the presence of chronic diseases.

#### Interventions

Studies that evaluated group-based interventions targeting healthy eating were eligible. Examples of modes of delivery included interventions based on nutrition, education, gardening and congregate dining. If studies reported on interventions with multiple delivery modes, only group-based interventions were extracted and analysed. Programmes focussed on weight management or weight loss were excluded. Interventions delivered in any community-based setting were eligible, including seniors’ and community centres. Studies that took place in acute or long-term care settings were excluded.

#### Comparators

Studies that compared an intervention to any comparison group (including single group pre-test/post-test) were eligible. Examples of comparator groups included pre-intervention, other intervention or non-exposed control groups.

#### Outcomes

Studies that reported on a change in nutrition outcomes from pre- to post-intervention were eligible for inclusion. Nutrition outcomes were grouped retrospectively into three categories: (1) food and fluid intake (e.g. vegetables and fruit, whole grain foods and protein), (2) nutrition risk, defined as factors that impact food intake^(32)^ (e.g. dietary habits, food access) and (3) healthy eating knowledge (e.g. nutrient functions, recommended servings). Physical mobility outcomes were considered secondary outcomes and were retrospectively grouped into two categories: (1) physical activity and (2) functional outcomes (e.g. Timed Up and Go test, gait speed).

### Assessment of methodological quality

Two independent reviewers critically appraised all eligible studies for methodological quality using the Joanna Briggs Institute critical appraisal instruments for experimental or quasi-experimental studies^(33)^. Overall scores for each study were calculated by responses to the questions. Any disagreements between reviewers were resolved through discussion or input from a third reviewer.

### Data extraction

Two independent reviewers performed data extraction in duplicate using a pre-developed and tested data extraction form. This form included general study information (i.e. study aim, design, country, start/end dates), population (i.e. age, sex, number of participants, ethnicity, socioeconomic status), intervention details (including duration, frequency, who delivered, how it was delivered, where it was delivered and theoretical framework, with questions framed according to the Template for Intervention Description and Replication (TIDieR) checklist and guide^(34)^), comparison groups, limitations and conclusions reported by study authors. Relevant nutrition and mobility outcomes were also extracted for all time points reported in the individual studies. When measures of overall food and fluid intake were reported (e.g. Food Frequency Score, Dietary Variety Score), these were extracted over specific food group intake results. Any disagreements between reviewers were resolved through discussion or by a third reviewer. Data collection forms and extracted data used for analyses are available upon request.

### Data synthesis

A meta-analysis was not possible given the variation in intervention types and outcomes across included studies. A narrative approach was used to synthesise included studies^(35)^, with data summarised and presented in supporting tables. Results tables with effect size measures, including mean differences, odds ratio, effect sizes and proportional changes, were structured by intervention category and outcome measures to explore variation and possible sources of heterogeneity. When only pre-test/post-test means or percentages were reported, mean or percent differences between groups were calculated. When missing, mean differences, confidence intervals and/or standard deviations of the changes were calculated using accepted equations^(36)^ and RevMan software^(37)^. A correlation coefficient of 0·5 was estimated for both food and fluid intake outcomes^(38–42)^ and physical activity outcomes^(43–45)^, based on available literature. Reporting bias was not explored as most studies did not cite a protocol or trial registration. Sensitivity analyses were not performed. A comprehensive approach to assess the overall certainty of the evidence for each outcome was not used due to high heterogeneity across interventions and outcomes.

## Results

### Description of included studies

**Fig. 1:**
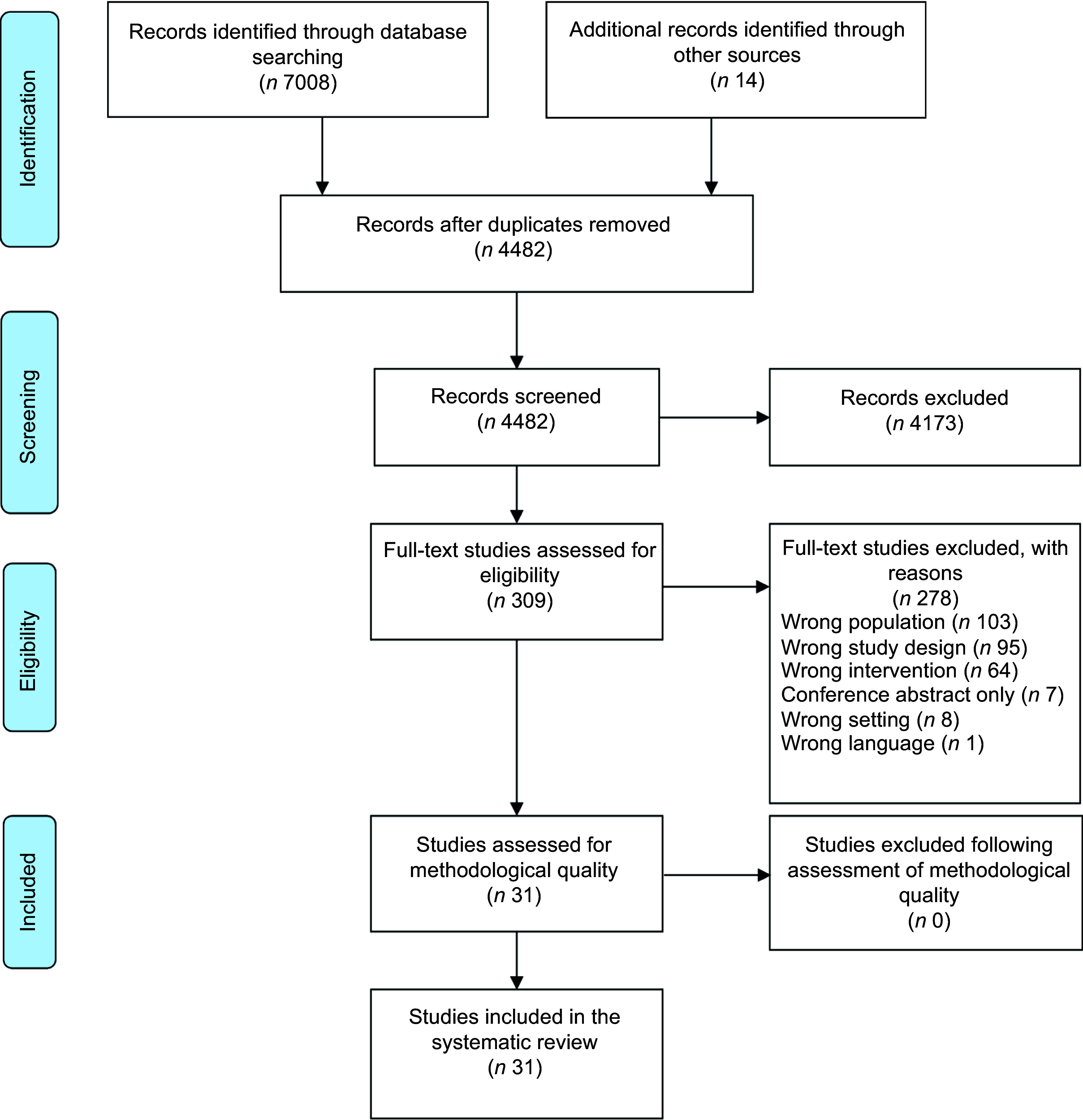
PRISMA flow diagram

The search resulted in 4482 unique records, of which 309 were identified as potentially relevant and underwent full-text review (Fig. 1). A total of thirty-one studies met all eligibility criteria and were included in the analysis (Table 1), including eleven single group, pre-test/post-test studies^(46–56)^, ten RCT^(57–66)^ and ten non-randomised, two group study designs^(67–76)^. A list of excluded studies with reasons for exclusion is provided in Supplemental Appendix 1. Studies were most often conducted in North America (*n* 20, 65 %), with the remainder in Asia (*n* 7, 23 %), Europe (*n* 3, 9 %) and Australia (*n* 1, 3 %). The total number of participants enrolled was 6723 (Range: 10–761), with high loss to follow-up noted (Range: 0–65 % where reported; 48 % (*n* 15) reported > 20 % attrition). Mean age ranged from 64 to 82 years (range 50–98 years when mean age was not reported). Most participants were female, with 74 % (*n* 23) of studies reporting > 70 % female participants. Nine studies (29 %) explicitly targeted low-income or economically disadvantaged populations^(46,51,53–55,62,64,68,70)^.

**Table 1:** Characteristics of included studies (*n* 31)

Study	Design	Country	Description of intervention/comparator	Intervention details	Population	Race/ethnicity (%)	SES	*n* started (completed) trial	Mean age, years (sd)	Sex (% F)
Abusabha 2011^(46)^	Single group pre/post	USA	I: Veggie Mobile van delivers discounted fresh produce to low-income neighbourhoods C: None	Duration: 6 months Frequency: 1 h/week Who? NR How? In-person Where? Two senior housing sites TF: NR	Community-dwelling seniors, ≥ 55	White: 58; Black: 39	Income < $10 000/year: 51 %	79 (43)	68·2 (9)	82
Beasley 2019^(47)^	Single group pre/post	USA	I: Diabetes Prevention Programme, including reducing calories and fat, overall healthy eating, PA and managing eating triggers. C: None	Duration: 6 weeks Frequency: 1 h/week Who? Certified group facilitator How? Interactive webinars Where? Participants at senior centre, facilitator remote TF: NR	Older adults ≥ 60 with diabetes risk score ≥ 5	White: 56; Black/African American: 38; Hispanic: 8; Asian: 6	High school: 6 %, some college or technical school: 31 %, college: 63 %	16 (12)	70·1 (5·6)	69
Brewer 2016^(67)^	Non-randomised, two groups	USA	I: FV nutrition education (e.g. phytochemicals, serving sizes shopping) and educational tools (e.g. recipe cards, phytochemical guide, health information) C: Educational tools	Duration: NR Frequency: 5 × 15 min Who? Research personnel How? In-person lessons + handouts Where? Congregate dining programmes at senior centres TF: NR	Community-dwelling, older adults, ≥ 60	White, I: 84, C: 81	At least high school, I: 74 %, C: 88 %	64 (35, I: 19, C: 16)	I: 74·1 (8·4) C: 77·6 (8·2)	I: 79 C: 88
Chung 2014^(68)^	Non-randomised, two groups	Hong Kong	I1: Nutrition seminars covering nutrient classification, healthy foods and labelling, recipes, cooking demo. Provided ingredient samples for low-cost, nutrient-rich meals with 1-day food samples/week I2: As above with three, 1-day food samples/week	Duration: 3 weeks Frequency: 1×/week Who? Nutritionists How? In-person; cooking steps via video Where? Mobile integrative health centre TF: NR	Elderly adults ≥ 55 living independently, without cognitive or mobility disabilities	NR	All lived in subsidised housing	60, I1: 30, I2: 30 (22, I1: 9, I2: 13)	74·4 (7·8)	83
Francis 2014^(57)^	RCT	USA	I: Nutrition and health education including FV and Ca-rich food; PA; safe food handling; food security. Group discussion of smarter goal planning and taste-testing activity. C: Didactic education (newsletters) only	Duration: 6 months Frequency: Monthly Who? Program educator How? Newsletter + 30 min in-person discussion and facilitated education Where? four urban congregate meal sites TF: Social Marketing Theory, Health Belief Model	Older adults ≥ 60	White: 80; Black/ African American: 15	High school or less: 26·7 % Some college: 36·7 % Bachelors: 33·4 %	73 (60, I: 29, C: 31)	72·6, range 55–88	57
Gallois 2013^(69)^	Non-randomised, two groups	Germany	I: Tools to track FV, dairy products, fish, and PA; performance feedback and advice. Standard health info on PA, nutrition, recipes. C: Standard health info and recipes by mail	Duration: 3 months Frequency: 7 × 45–60 min Who? Trained moderators How? In-person discussion (6–10 participants), handouts Where? Community partners’ institutions, churches, mosques TF: Kanfer’s Self-regulation Model	Elderly people ≥ 57 with the ability to care for oneself	German, I: 90, C: 85; USSR, I: 2, C: 10; Turkish, I: 8, C: 5	Low SES neighbourhood, I: 41 %, C: 28 %; High SES neighbourhood, I 59 %, C: 73 %	423 (369)	Range 57–95	I: 82 C: 77
Geller 2012^(58)^	RCT	USA	I1: Decisional balance sheet for FV intake. Provides basic health knowledge and empowers individuals to consider pros and cons of behaviour adoption. I2: Identical programme targeting PA instead of FV	Duration: 1 d Frequency: Once Who? NR How? In-person, group discussion, guided completion of decisional balance sheet Where? two community housing sites TF: NR	Older adults residing in community living homes	Japanese: 24; Filipino: 19; Caucasian: 19; Native American: 5; Native Hawaiian: 5; Hispanic: 5; Other: 24	80 % graduated high school	34 (21, I1: 9, I2: 12)	72·2 (11·8)	76
Hersey 2015^(70)^	Non-randomised, two groups	USA	I: ‘Eat Smart, Live Strong’ nutrition education, including FV intake and PA goal setting; recipe modification; food assistance resources and community programmes; recipe cards; fact sheets. C: Waitlist control	Duration: 4 weeks Frequency: 65 min/week Who? Nutrition educators How? In-person interactive education + handouts Where? Low-income senior centres in urban and rural communities TF: BEHAVE Decision-Making Theory	Older adults ≥ 60	White: 69; Black: 19; Native American: 10; Hispanic or Latino: 8; Asian or > 1 race: 2	Low-income older adults	614, I: 267, C: 347 (603, I: 263, C: 340)	Range 60–80	I: 84 % C: 68 %
Hsu 2010^(71)^	Non-randomised, two groups	Taiwan	I: Nutrition education and practice via dietary choice games (food categories, healthy diet, cooking principles, food recognition), guided by CCAA and NIA materials. PA component (endurance, strength, balance, flexibility) C: No intervention	Duration: 12 weeks Frequency: 1, 3 h session and two phone call follow-up reminders Who? Physical therapist, assistant trainers How? In-person; PA demo via video; brochure; follow-up via phone Where? three community public health centres TF: NR	Community-based elderly ≥ 65	Mingnan, I: 24, C: 34; Hakka, I: 69, C: 58; Mainlander, I: 7, C:8	NR	584, I: 290, C: 294 (514, I: 259, C: 255)	Range 65–80+	51
Jancey 2017^(59)^	RCT	Australia	I: PA and nutrition education (e.g. goal setting, monitoring and feedback; skill building; social support; exercise demo); educational resources (booklet, calendar, exercise chart, resistance bands, newsletters); motivational interviewing (goal setting, adherence, sustainability) C: No intervention	Duration: 6 months Frequency: Tailored to participant needs (weekly to monthly) Who? Peer-trained programme ambassadors How? Educational resources, two face-to-face meetings, motivational interviewing via telephone Where? Retirement village TF: social cognitive theory	Older adults residing in retirement villages; not currently active or on special diet	NR	51 % completed Secondary school or less, 20 % certificate or diploma, 29 % University degree	363, I: 197, C: 166 (280, I: 139, C: 141)	72 (5·2)	75
Kimura 2013^(60)^	RCT	Japan	I: ‘Sumida TAKE10’ program. Lecture on good dietary habits; participants self-monitored dietary check sheets during lecture and received instructor feedback; stretching and strengthening exercise. C: Crossover	Duration: 3 months Frequency: 1·5 h/ biweekly Who? Researchers and staff How? In-person 30 min lecture/1 h exercise; home exercise and diet tracking Where? six community centres TF: NR	Community-dwelling older adults ≥ 65	NR	NR	94, I: 57, C: 37 (92, I: 57, C: 35)	I: 74·3 (5·9), C: 74·3 (5·0)	I: 84 C: 77
Lara 2015^(61)^	RCT	England	I: Group education including benefits of Mediterranean diet, shopping tips, meal planning. Material package including guidelines, menus, recipes; asked to adopt for 3 weeks. C: Educational group session and package (without menus, recipes or follow up)	Duration: 3 weeks Frequency: 1, 2 h session Who? Nutritionist, with research team support How? In-person, interactive educational group session (PowerPoint + discussion) + 10–15 min follow up phone calls on days 3, 11, 16 Where? Newcastle University TF: NR	Healthy older adults ≥ 50	NR	83 % retired	23, I: 13, C: 10 (23, I: 13, C: 10)	66 (9)	NR
Lillehoj 2018^(72)^	Non-randomised, two groups	USA	I: Supplemental Nutrition Assistance Program-Education (SNAP-Ed) including goal setting, recipe tasting, PA break. C: No intervention	Duration: 9 months Frequency: 30 min/months Who? Trained facilitators How? In-person, facilitative, non-didactic, discussion + newsletter Where? Congregate meal sites TF: Health Belief Model	Adults ≥ 60 from congregate meal sites	White: 92; Black: 2; Hispanic: 1; Asian: 1; Other: 1; Missing: 4	74·4 % High or marginal food security	761, I: 419, C: 342 (269, I: 121, C: 148)	78·6 (NR)	75
Luten 2016^(73)^	Non-randomised, two groups	Netherlands	I: Community-based media campaign to promote healthy eating and PA C: Region where no intervention took place	Duration: 3 months high intensity, 6 months low intensity Frequency: 244 posters, 600 radio broadcasts, 20 radio interviews, 4 newspaper ads Who? Local peers and healthcare professionals How? Posters, radio, newspaper Where? Community TF: Integrated Model for Change, ANGELO, Ecological Model	Healthy, community-based older adults ≥ 55	NR	Socio-economically disadvantaged areas I: 38·7 % C: 58·6 %	643, I: 430, C: 213 (564, I: 379, C: 185)	I: 66·2 (7·8), C: 67·0 (7·8)	I: 61 C: 56
MacNab 2017^(74)^	Non-randomised, two groups	USA	I1: Interactive whole grain nutrition education programme, hands-on activities to identify whole grains, case scenarios to apply knowledge, taste-testing, worksheets, handouts, recipes I2: Modified intervention based on delivery style (same activities)	Duration: 3 weeks Frequency: 1 h/week Who? Instructor How? I1 via PowerPoint with small group discussion, I2: discussion only (no PowerPoint) Where? Senior apartments, retirement communities, senior centres TF: Social Marketing Theory	Community residing adults ≥ 60	White: 96; Other: 4	High school, GED or less: 31·8 % Some college or degree: 32·5 % Bachelor’s degree or higher: 35·0 %	174 (157)	60–70: 28·7 % 71–80: 35·7 % 81+: 35·7 %	89
Manafo 2013^(48)^	Single group pre/post	Canada	I: Nutrition Information Series following Canada’s Food Guide to Healthy Eating; interactive activities including making a food record and reading food labels; healthy snack C: None	Duration: 12 week Frequency: 1×/week Who? NR How? In-person slideshow, discussion, Q&A, handouts; interpreters at each session Where? three neighbourhoods (only one included in analysis due to attendance) TF: NR	Seniors ≥ 55	Chinese, Persian, Filipino, Tamil (%NR)	NR	55 (24)	55–65: 17 % 66–75: 54 % 75+: 29 %	100
Meethien 2011^(62)^	RCT	Thailand	I: Nutrition education for elders and family members. Individual counselling; motivational plan for healthy eating; food preparation activities; training and guidance on meal planning; personal goal setting, behavioural monitoring, and maintenance C: Usual care	Duration: 3 months Frequency: 1×/week Who? Nurses How? In-person group discussion, phone, handouts Where? two community study sites + counselling in elder’s home TF: Pender’s Health Promotion Model	Elders ≥ 60 residing with at least one family member who is responsible for selecting and preparing their meals	Thai Buddhists	Low SES; participants perceived income as inadequate	180, I: 90, C: 90 (166, I: 86, C: 80)	I: 67·4 (6·6), C: 66·6 (5·5)	I: 65 C: 60
Mendoza-Ruvalcaba 2015^(63)^	RCT	Mexico	I: ‘I am Active’ alternating sessions on nutrition or cognitive function; meal planning; goal setting; strength, balance, and mobility physical exercises C: Waitlist control, weekly social activities	Duration: 2 months Frequency: 2 h, 2×/week Who? Trainer How? In-person presentation, take-home activities; 30-min PA Where? Senior centre TF: WHO Model for Active Aging	Healthy adults ≥ 60 from senior centres	NR	Years of education I: 5·55 (3·12) C: 3·97 (3·28)	64, I: 31, C: 33 (57, I: 27, C: 30)	I: 70·5 (6·4), C: 70·8 (7·2)	I: 94 C: 89
Moreau 2015^(49)^	Single group pre/post	Canada	I: Nutrition education and cooking workshops including healthy eating, cancer, CVD prevention, nutrition for aging, labels, fibre, bone health, eating for pleasure, social support, barriers and strategies, recipes, take-home meals. C: None	Duration: 8 weeks Frequency: 2 h/week Who? RD How? In-person interactive education, discussion, handouts. Out-of-pocket user fees ($20) for ingredients used, taken home for later consumption. Where? Community kitchen TF: NR	Community-dwelling adults ≥ 50	NR	NR	154 (144)	50–59: 14·7 % 60–69: 52·4 % 70+: 32·9 %	87
Murayama 2020^(75)^	Non-randomised, two groups	Japan	I: Drama-style lectures on nutrition (protein, fat, carbohydrates) and dietary variety; food tasting; discussion to share knowledge, success and failures; home activities. C: Crossover	Duration: 8 weeks Frequency: 2 h, Biweekly Who? Trained community health workers How? In-person, 30–40 min lecture, 60 min discussion, 20–30 min meal tasting Where? Community centre TF: NR	Community-dwelling older people aged 65–74	NR	‘Normal’ financial stability, I: 73 %, C: 70 %; ‘poor’ financial stability, I: 10 %, C: 0 %	84, I: 41, C: 43 (78, I: 41, C: 37)	I: 68·8 (3·0), C: 69·1 (3·4)	I: 63 C: 73
Pogge 2013^(50)^	Single group pre/post	USA	I: ‘Mindful Choices’ topics included calories, goal setting, building a support system, portion control, PA, nutrition, food labels, stress management. Snacks, tip sheets and calorie counting books provided. C: None	Duration: 12 weeks Frequency: NR (1 h sessions, $50 incentive after 10 sessions) Who? RD, exercise director, pharmacist How? In-person classroom style with PowerPoint presentation, discussion Where? Seniors independent living campus TF: NR	Independent living seniors	100 % Caucasian	Income < 20 000: 26·1 % 20 000–30 000: 8·7 % 30 000–40 000: 13 % > 40 000: 21·7 % NR: 30·4 %	30 (23)	82 (5·0)	87
Salehi 2011^(64)^	RCT	Iran	I: Group-based tailored nutrition intervention based on stages of change aiming for 5 FV servings/d. Included goal setting, action planning, reinforcement. C: 4 weekly general health education	Duration: 4 weeks Frequency: 90 min/week Who? NR How? In-person, 40 min PowerPoint, 30 min discussion, 10 min Q&A, 10 min reception with FV Where? ten elderly centres TF: Transtheoretical Model	Community-based elderly ≥ 60 from existing elderly centres	NR	Low income: 76·5 % Moderate income: 16·2 % High income: 7·3 %	400, I: 200, C: 200 (NR)	64·1 (4·5)	75
Schwingel 2017^(51)^	Single group pre/post + qualitative	USA	I: Nutrition education and culturally tailored lifestyle change curriculum, including healthy living, healthy eating, nutrition labels, buying healthy food, stress management, barriers, goal setting, action plans, home activities (e.g. healthy meal prep, grocery shopping with grandchildren, PA, pedometer step-tracking), motivational telephone calls C: None	Duration: 6 months active, 3 months maintenance Frequency: six workshops (frequency NR); weekly (active phase) and bi-weekly (maintenance phase) phone calls Who? Trained Promotoras How? In-person educational workshops (lectures plus group discussion and hands-on activities) + individual meetings, at home activities Where? Church facilities TF: Transtheoretical Model, Social Cognitive Theory	Healthy, Latina women aged ≥ 50	Latina	41 % employed 88 % encounter financial difficulty covered daily expenses	34 (19)	64 (8)	100
Silva-Smith 2013^(65)^	RCT	USA	I: ‘Promoting Older Adult Wellness’, education, social network, motivational support and short/long-term goal setting (for PA and DASH diet); supervised, progressive walking programme C: Attention control health newsletters	Duration: 8 weeks Frequency: 1 h/week Who? Trained interventionist delivered sessions, lay health advisor (older adult) for social and motivational support How? Group session, workbook, newsletters Where? Community health centre TF: Wellness Motivation Theory	Community-dwelling overweight/obese adults ≥ 60, sedentary or recently physically active and able to participate in walking	White, I: 75, C: 65; African American, I: 13, C: 19	Median monthly income $1000–1399	69, I: 32, C: 37 (63, I: 29, C: 34)	I: 71·3 (7·4), C: 67·8 (6·7)	I: 81 C: 84
Smith 2015^(52,95)^	Single group pre/post	USA	I: Texercise Select. Education on healthy dietary habits and cooking; PA and nutrition logs; goal setting, action plans and group brainstorming; PA component incorporating flexibility, strength, balance and endurance. C: None	Duration: 10 weeks Frequency: 1·5 h, 2×/week Who? Trained lay facilitators How? In-person workshops + interactive group discussion + 30–45 min guided exercise Where? Senior centres, community facilities, faith-based organisations and senior housing TF: Social Cognitive Theory	Primarily marketed to adults ≥ 55 (although ≥ 45 allowed to participate)	White: 83; Black/African American: 11; American Indian/ Alaska Native: 2; Asian: 1; Native Hawaiian or Pacific Islander: 1; Other: 3	Less than high school: 4·7 % Some high school: 9·5 % High school graduate or equivalent: 27·4 % Some college or vocational school: 37·9 % College graduate or higher: 20·5 %	220 (127)	74·9 (8·4)	85
Smith 2020^(76,96)^	Non-randomised, two groups	USA	I: Texercise Select. Education on healthy dietary habits and cooking; PA and nutrition logs; goal setting, action plans and group brainstorming; PA component incorporating flexibility, strength, balance and endurance. C: Usual care, waitlist control	Duration: 10 weeks Frequency: 1·5 h, 2×/week Who? twp trained lay leaders How? In-person workshops + interactive group discussion + 30–45 min guided exercise Where? Senior centres, faith-based and senior housing facilities and community centres TF: Social Cognitive Theory, Socioecological Framework	Middle-aged or older adults ≥ 45 (adults ≥ 60 focal target age group)	Non-Hispanic white: 47	High school or lower: 39 %, Some college: 31 %, College graduate: 30 %	430, I: 163, C: 267 (182, I: 74, C: 108)	74·5 (9·0)	77
Strout 2017^(53)^	Single group pre/post + qualitative	USA	I: ‘GROW’ (Green Organic Vegetable Gardens). Participants given a raised garden bed, ergonomic tools and supplies and chose seeds and recipes. C: None	Duration: 17 weeks Frequency: 1 h/week Who? Gardening expert from partnering university How? In-person Where? Congregate housing site TF: NR	Independent community-dwelling older adults ≥ 65	NR	Low-income senior housing site	10 (NR)	77·4, Range 67–89·5	80
Thomas 2010^(54)^	Single group pre/post	USA	I: Educational booklet including nutrition knowledge, recommended food items and PA to improve or prevent chronic diseases C: None	Duration: 1 month Frequency: 5 d/week Who? Booklet with congregate meal site director re-enforcement How? Passive distribution Where? Community and senior centres, seniors’ apartments, retirement facilities, high schools, salvation army, churches and fire stations TF: NR	Rural older adults ≥ 65	Caucasian: 81; Black/African American: 13	Low income, economically disadvantaged	432 (187)	Range 52–98 < 60: 2·6 %, 60–74: 36·7 %, 75+: 60·7 %	70
Turk 2016^(55)^	Single group pre/post	USA	I: ‘Wise Choices’ sessions focused on nutrition and PA, including FV, Ca, and fibre intake; portion sizes; USDA MyPlate food choices; step goals using pedometers given. C: None	Duration: 12 weeks Frequency: 45 min/week Who? Trained doctoral students How? In-person, info sheets, 10–15 mins of walking or activity Where? Senior centres, senior high-rise, family support centre TF: NR	Older adults ≥ 50, regular diet, ambulating independently or with assistive device	White: 53; Black: 44; Asian: 1; Biracial: 2	Low-income neighbourhoods Household income < $20 000: 59 %, $20 001–$50 000: 38, > $50 000: 4 %	118 (101)	71·7 (9)	88
Uemura 2018^(66)^	RCT	Japan	I: Educational health promotion on exercise, diet, nutrition, cognitive activity including malnutrition, food labelling, walking, resistance exercise. PA self-monitored via accelerometer. Self-planning for and implementing behavioural change C: No intervention	Duration: 24 weeks Frequency: 9 in/week Who? Licensed physical therapists and physical education teachers How? Exploratory learning, group work, discussion, homework Where? Classroom setting, location NR TF: NR	Rural, community-dwelling elders ≥ 65	Japanese	Average 12·9 years of education I: 83·3 % unemployed C: 69·0 % unemployed	84, I: 42, C: 42 (79, I: 40, C: 39)	I: 72·1 Range 65–83	I: 69 C: 71
									C: 71·6 Range 65–85	
Wunderlich 2011^(56)^	Single group pre/post	USA	I: Elderly Nutrition Programme, including education sessions focused on common conditions among older adults (e.g. hypertension and salt intake) and cooking demos. C: None	Duration: 2 years Frequency: Quarterly Who? Nutritionists How? Classroom format, 30–40 min lessons and interactive discussions, 1 h activity, Q&A, handouts. Optional free individual phone counselling. Where? Social and community centres, senior centres, churches, schools TF: NR	Seniors ≥ 60 at congregate meal sites	‘Predominantly white, followed by Black and Hispanic’	42·8 % ‘below poverty’	354 (259)	74·5	68·2

NR, not reported; PA, physical activity; FV, fruits and vegetables; RCT, randomised controlled trial; SES, socio-economic status; CCAA, Canadian Center for Activity and Aging, NIA, National Institute of Aging.

Four main intervention categories were identified: (1) nutrition education with behaviour change techniques (BCT) (*n* 21, 68 %)^(48,50–52,56–60,62–66,69–72,74–76)^, (2) didactic nutrition education (e.g. lectures, handouts) (*n* 4, 13 %)^(54,55,67,73)^, (3) interactive nutrition education (e.g. workshops, discussion) (*n* 2, 6 %)^(47,61)^ and (4) food access (e.g. mobile markets, gardening, food samples) (*n* 2, 6 %)^(46,53)^. Two studies (6 %) combined nutrition education with BCT and food access^(49,68)^. The BCT Taxonomy^(77)^ was used to identify interventions that incorporated BCT when components such as goal setting, action planning, feedback and monitoring, social support (e.g. motivational interviewing), shaping knowledge through instruction on how to perform a behaviour (e.g. cooking demonstrations) and behavioural practice/rehearsal (e.g. healthy food selection or recognition activities) were explicitly described. Physical activity education was reported as a co-intervention in 9 (29 %) studies^(47,50,54,57–59,69,70,73)^, and physical activity participation within sessions (e.g. strengthening, walking, step tracking) was reported in 10 (32 %) studies^(51,52,55,60,63,65,66,71,72,76)^.

Median intervention duration was 12 weeks (range 1 day to 2 years). Session frequency was variable, with weekly delivery most common (*n* 13, 42 %)^(46–49,53,55,62,64–66,68,70,74)^. Interventionists included trained facilitators (*n* 6, 19 %)^(47,51,65,69,72,74)^, research personnel (*n* 4, 13 %)^(54,55,60,67)^, educators (*n* 3, 10 %)^(53,57,70)^, nutritionists (*n* 3, 10 %)^(56,61,68)^, physiotherapists and/or trainers (*n* 3, 10 %)^(63,66,71)^, registered dietitians (*n* 2, 6 %)^(49,50)^, healthcare providers (*n* 2, 6 %)^(62,75)^, lay leaders (*n* 2, 6 %)^(52,76)^ and peer leaders (*n* 2, 6 %)^(59,73)^. Four studies (13 %) did not report interventionist details. Programmes were delivered within congregate meal sites (*n* 5, 16 %)^(54,56,57,67,72)^, seniors’ housing sites (*n* 5, 16 %)^(46,50,53,58,59)^, seniors’ centers (*n* 4, 13 %)^(47,63,64,70)^, community health centers (*n* 3, 10 %)^(65,68,71)^, community centers/kitchen (*n* 3, 10 %)^(49,60,75)^, a church facility (*n* 1, 3 %)^(51)^ and a university (*n* 1, 3 %)^(61)^, with 7 (23 %)^(52,55,62,69,73,74,76)^ delivered across multiple community settings and 2 (6 %) not reporting setting. Theoretical models were applied in 45 % of studies (*n* 14); the most common were Social Cognitive Theory (*n* 4, 13 %)^(51,52,59,76)^, Social Marketing Theory (*n* 2, 6 %)^(57,74)^, Health Belief Model (*n* 2, 6 %)^(57,72)^ and the Transtheoretical Model (*n* 2, 6 %)^(51,64)^. Multiple theories were often combined within studies, although none applied them in the same manner.

### Methodological quality

**Fig. 2:**
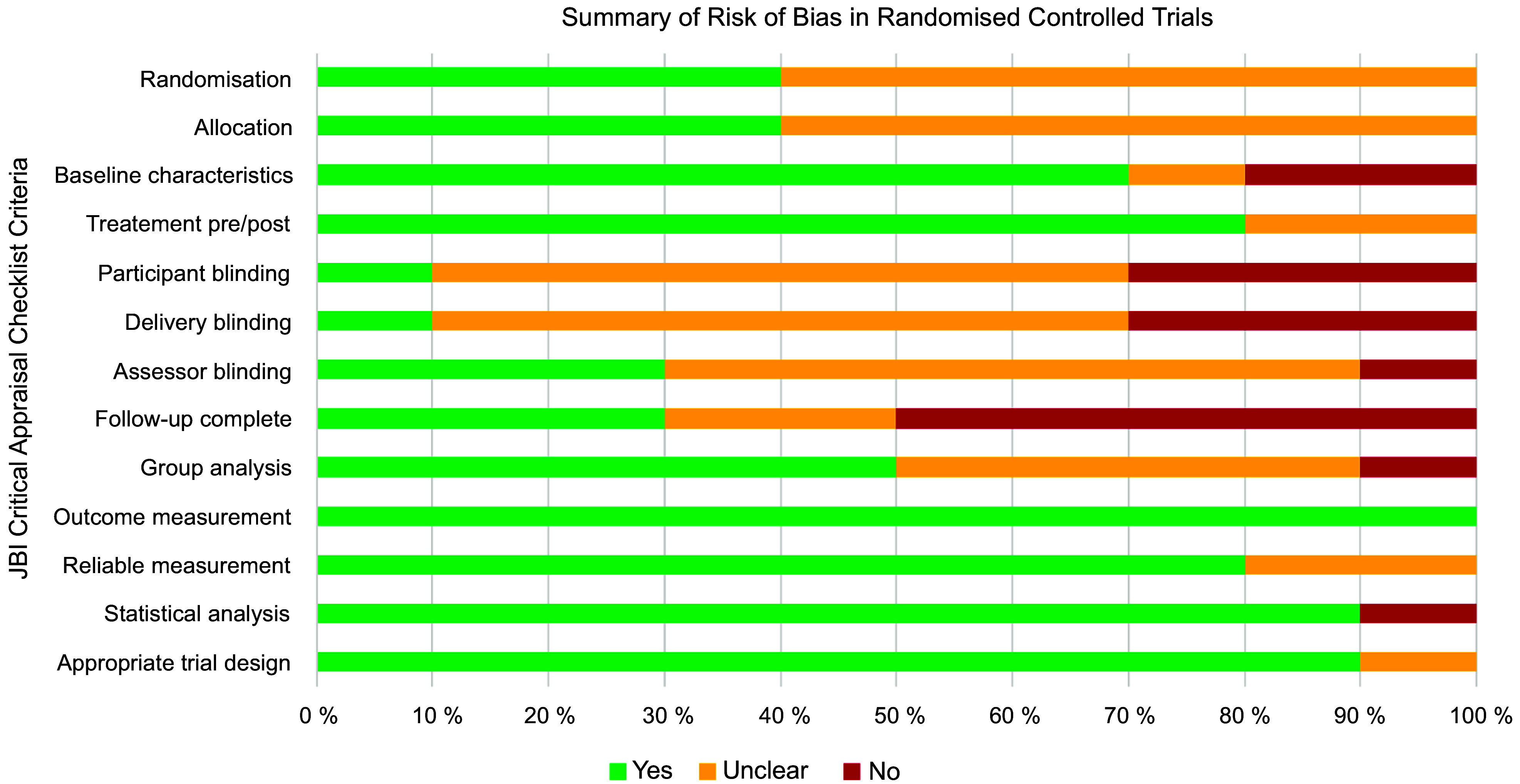
Summary of risk of bias in randomised controlled trials (*n* 10). Assessed using JBI Critical Appraisal Checklist for Randomised Controlled Trials

**Fig. 3:**
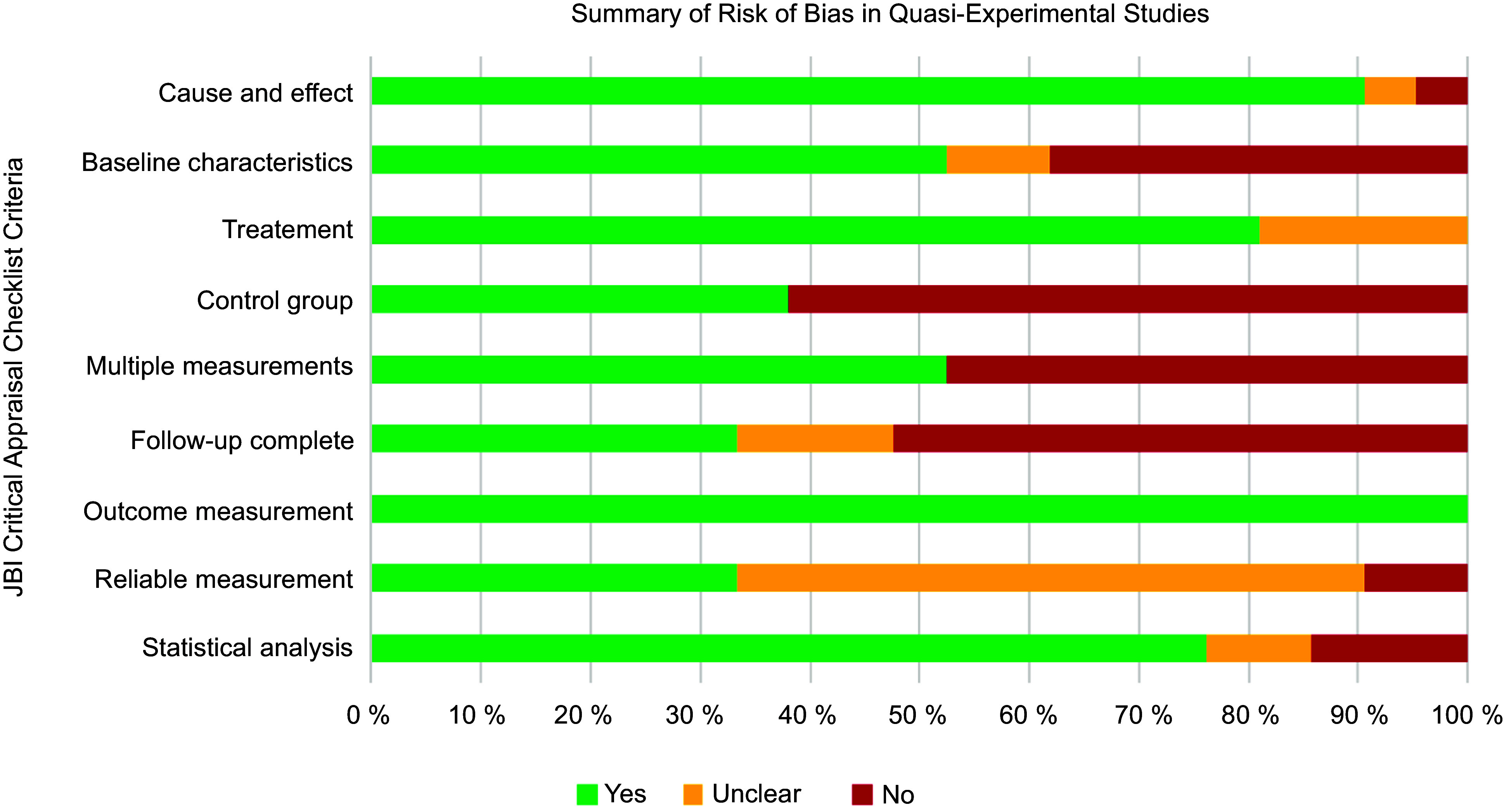
Summary of risk of bias in quasi-experimental studies (*n* 21). Assessed using JBI Critical Appraisal Checklist for Quasi-Experimental Studies (includes single-group, pre-test/post-test and two-group, non-randomised study designs)

The ten RCT had a generally unclear or high risk of bias (Fig. 2). Only one study reported blinding of participants and delivery personnel^(61)^. There was unclear or no blinding of outcome assessors in 70 % of RCT (*n* 7)^(57,58,60–64)^, and 70 % (*n* 7) did not adequately describe or analyse differences between groups when incomplete follow-up was reported^(57–60,62–64)^. Selection bias was a concern, given that 60 % of the RCT (*n* 6) did not adequately report procedures for randomisation^(57,58,60,62–64)^ and allocation concealment^(57,58,62–64,66)^. Similarly, the twenty-one quasi-experimental studies had an unclear or high risk of bias (Fig. 3) due to lack of a comparator group (*n* 13, 62 %)^(46–56,68,74)^, inadequate description and analysis of groups when incomplete follow-up was reported (*n* 14, 67 %)^(46,48–52,54,56,67,69,71,72,74,76)^ and unreliable outcome measurements (*n* 14, 67 %)^(46,48–50,52–56,71,73–76)^. Full critical appraisal findings for each study are available in Supplemental Tables 6–7.

### Nutrition outcomes

#### Food and fluid intake

The twenty-two interventions assessing food and fluid intake included nutrition education with BCT (*n* 14, 64 %)^(51,52,56,58–60,64–66,69,70,74–76)^, didactic nutrition education (*n* 3, 14 %)^(55,67,73)^, interactive nutrition education (*n* 2, 9 %)^(47,61)^, food access (*n* 2, 9 %)^(46,53)^ and nutrition education with BCT and food access (*n* 1, 4 %)^(49)^. Food and fluid intake (e.g. vegetables and fruit, water and whole grains) were captured using a variety of tools, such as FFQ (*n* 10, 45 %)^(49,51,52,55,60,64,66,67,69,75)^, 24-hour diet recalls (*n* 3, 14 %)^(51,65,69)^ and food records (*n* 2, 9 %)^(47,61)^ (Table 2).

**Table 2:** Food and fluid intake (*n* 22)

Study ID	Description of intervention/comparator	Data collection tool	Outcome	Effect size (95 % CI or sd, *P*-value)	Risk of bias
**Nutrition education with behaviour change technique interventions**					
Gallois 2013^*^^(69)^	I: Tools to track FV, dairy products, fish, and PA; performance feedback and advice. Standard health info on PA, nutrition, recipes. C: Standard health info and recipes by mail	24-hour diet recall	Five servings FV/d, I *v*. C at end of study	Adjusted OR 1·29 (0·84, 1·96)	7/9
			Three servings dairy products/d, I *v*. C at end of study	Adjusted OR 1·09 (0·71, 1·68)	
		FFQ	One serving fish/week, I *v*. C at end of study	Adjusted OR 0·94 (0·54, 1·64)	
Geller 2012^*^^(58)^	I1: Decisional balance sheet for FV intake. Provides basic health knowledge and empowers individuals to consider pros and cons of behaviour adoption. I2: Identical programme targeting PA instead of FV	National Health and Nutrition Examination Survey single item instrument	FV intake (servings/d)	I1: MD –0·74 (2·82), descriptive statistics only I2: MD 0·27 (2·97), descriptive statistics only	5/13
Hersey 2015^*^^(70)^	I: ‘Eat Smart, Live Strong’ nutrition education, including FV intake and PA goal setting; recipe modification; food assistance resources and community programmes; recipe cards; fact sheets. C: Waitlist control	University of California Cooperative Extension Food Behaviour Checklist	FV intake (cups/d)	**MD 0·52 (0·23, 0·82)**	8/9
			Fruit intake (cups/d)	**MD 0·2 (0·01, 0·38)**	
			Vegetable intake (cups/d)	**MD 0·31 (0·16, 0·47)**	
Jancey 2017^*^^(59)^	I: PA and nutrition education (e.g. goal setting, monitoring and feedback; skill building; social support; exercise demo); educational resources (booklet, calendar, exercise chart, resistance bands, newsletters); motivational interviewing (goal setting, adherence, sustainability) C: No intervention	Fat and fibre Barometer	% Participants consuming > 2 servings fruit 3–7 d/week	**MD 11·3 %,** ***P *** **= 0·007**	10/13
			% Participants consuming > 2 two servings vegetables 3–7 d/week	MD 4·3 %, *P* = 0·052	
			Fibre intake score (range 1–5 with 1 indicating low fibre and 5 indicating high fibre)	MD 0·07 (–0·07, 0·21)	
			Fat intake score (range 1–5 with 1 indicating high fat and 5 indicating low fat)	MD 0·04 (–0·07, 0·15)	
			Fat avoidance score (range 1 to 5, interpretation NR)	MD –0·06 (–0·27, 0·15)	
Kimura 2013^*^^(60)^	I: ‘Sumida TAKE10’ programme. Lecture on good dietary habits; participants self-monitored dietary check sheets during lecture and received instructor feedback; stretching and strengthening exercise. C: Crossover	Food frequency intake questionnaire (tool NR)	Food Frequency Score (range 0 to 30, sum of intake scores across food groups)	**MD 2·7 (0·79, 4·61)**	7/13
			Dietary Variety Score Overall score (range 0 to 10, higher score indicates greater variety)	**MD 1·5 (0·42, 2·58)**	
MacNab 2017^(74)^	I1: Interactive whole grain nutrition education programme, hands-on activities to identify whole grains, case scenarios to apply knowledge, taste testing, worksheets, handouts and recipes I2: Modified intervention based on delivery style (same activities)	Dietary Screening Tool, three-item subscale	Total grain frequency score (max score 15, higher score indicates greater frequency)	**I1 and I2 combined: MD 0·9 (4·23),** ***P *** **< 0·001**	5/9
		Dietary Screening Tool, two-item subscale	Whole grain frequency score (max score 10, higher score indicates greater frequency)	**I1 and I2 combined: MD 0·7 (3·01),** ***P *** **< 0·001**	
Murayama 2020^(75)^	I: Drama-style lectures on nutrition (protein, fat, carbohydrates) and dietary variety; food tasting; discussion to share knowledge, success and failures; home activities. C: Crossover	Food frequency intake questionnaire (tool NR)	Dietary Variety Score Overall score (range 0 to 10, higher score indicates greater variety)	**MD 1·60 (0·75, 2·45)**	7/9
		Diet history questionnaire	Energy (kJ/d)	MD 548·61 (–296·69, 1393·90)	
			Protein (g/d)	MD 4·15 (–2·63, 10·94)	
			Fat (g/d)	**MD 5·46 (0·12, 10·8)**	
			Carbohydrate (g/d)	MD –8·90 (–27·85, 10·05)	
			Fibre (g/d)	**MD 1·75 (0·22, 3·27)**	
Salehi 2011^(64)^	I: Group-based tailored nutrition intervention based on stages of change aiming for 5 FV servings/d. Included goal setting, action planning, reinforcement. C: 4 weekly general health education	FFQ	FV consumption (servings/d)	**MD 1·26 (1·03, 1·49)**	4/13
Schwingel 2017^*^^(51)^	I: Nutrition education and culturally tailored lifestyle change curriculum, including healthy living, healthy eating, nutrition labels, buying healthy food, stress management, barriers, goal setting, action plans, home activities (e.g. healthy meal prep, grocery shopping with grandchildren, PA, pedometer step-tracking), motivational telephone calls C: Baseline	24-hour diet recall	# Fruits consumed/d	NS difference	7/9
			# Vegetables consumed/d	NS difference	
			# Fried foods consumed/d	**Decreased from baseline to end of study (** ***P *** **< 0·05)**	
			% Consumed 3 meals/d	**+30 % at end of study; maintained at follow up** (***P *** **< 0·05)**	
		FFQ	# Days fruits consumed/week	NS difference	
			# Days vegetables consumed/week	**Decreased from baseline to end of study (** ***P *** **< 0·05)**	
Silva-Smith 2013^*^^(65)^	I: ‘Promoting Older Adult Wellness’, education, social network, motivational support and short/long term goal setting (for PA and DASH diet); supervised, progressive walking programme C: Attention control health newsletters	24-hour diet recall	Fruit (servings/d)	MD –0·03 (90 % CI: –0·44, 0·38)	8/13
			Vegetable (servings/d)	MD 0·61 (90 % CI: –0·18, 1·39)	
			Grain (servings/d)	MD –0·13 (90 % CI: –1·15, 0·88)	
			Dairy products (servings/d)	MD 0 (90 % CI: –0·54, 0·54)	
			Meat and bean (servings/d)	MD 0·21 (90 % CI: –1·41, 1·82)	
			Fat (g/d)	MD 5·26 (90 % CI: –11·02, 21·53)	
			Total (kJ/d)	MD 420·45 (90 % CI: –786·22, 1627·12)	
			Na (mg/d)	MD 139·62 (90 % CI: –633·07, 912·31)	
			Cholesterol (g/d)	MD 21·82 (90 % CI: –68, 111·64)	
Smith 2015^*^^(52,95)^	I: Texercise Select. Education on healthy dietary habits and cooking; PA and nutrition logs; goal setting, action plans and group brainstorming; PA component incorporating flexibility, strength, balance, endurance. C: Baseline	FFQ	FV consumption (servings/week)	**MD 0·42,** ***P *** **= 0·002**	6/9
			Soda consumption (drinks/week)	MD –0·13, *P* = 0·255	
			Water consumption (cups/d)	**MD 0·59,** ***P *** **< 0·001**	
			Fast food consumption (times/week)	MD –0·21, *P* = 0·2	
Smith 2020^*^^(76,96)^	I: Texercise Select. Education on healthy dietary habits and cooking; PA and nutrition logs; goal setting, action plans and group brainstorming; PA component incorporating flexibility, strength, balance and endurance. C: Usual care, waitlist control	Diet recall (modified Starting the Conversation instrument)	FV consumption (proportional odds of a larger number of servings/week *v*. baseline)	End of study **I: OR = 1·68 (1·15, 2·47)** C: OR = 0·83 (0·60, 1·15), **I** ***v*** **. C,** ***P *** **= 0·006** Follow-up I: OR = 0·84 (0·53, 1·35) C: OR = 0·74 (0·50, 1·09), I *v*. C, *P* = 0·656	6/9
			Soda/sugar drink consumption (proportional odds of a larger number of drinks/week)	End of study I: OR = 0·88 (0·60, 1·30) C: OR = 1·09 (0·82, 1·44), I *v*. C: *P* = 0·393 Follow-up I: 0·73 (0·45, 1·19) C: 0·82 (0·58, 1·16), I *v*. C *P* = 0·71	
			Water consumption (proportional odds of a larger number of cups/d)	End of study **I: OR = 1·46 (1·10, 1·94)** C: OR = 1·14 (0·89, 1·47), I *v*. C: *P* = 0·204 Follow up: NR	
			Fast food consumption (proportional odds of a larger number of servings/week *v*. baseline)	End of study **I: OR = 0·66 (0·48, 0·91)** C: OR = 1·04 (0·77, 1·40), **I** ***v*** **. C:** ***P *** **= 0·046** Follow-up I: 0·65 (0·42, 1·02) C: 0·97 (0·66, 1·42), I *v*. C, *P* = 0·184	
Uemura 2018^*^^(66)^	I: Educational health promotion intervention on exercise, diet, nutrition, cognitive activity including malnutrition, food labelling, walking, resistance exercise. PA self-monitored via accelerometer. Self-planning for and implementing behavioural change C: No intervention	Food frequency intake questionnaire (tool NR)	Food Frequency Score (range 0 to 30, sum of intake scores across food groups)	**MD 2·49 (se 0·73),** ***P *** **= 0·001**	10/13
			Dietary Variety Score (Range 0 to 10, higher scores indicates greater variety)	**MD 0·81 (se 0·38),** ***P *** **= 0·04**	
Wunderlich 2011^(56)^	I: Elderly Nutrition Program, including education sessions focused on common conditions among older adults (e.g. hypertension and salt intake) and cooking demos. C: Baseline	Self-reported checklist	% Who consumed ≥ five servings FV/d	+3·4 %, *P* = 0·398	3/9
**Didactic nutrition education interventions**					
Brewer 2016^(67)^	I: FV nutrition education (e.g. phytochemicals, serving sizes, shopping) and educational tools (e.g. recipe cards, phytochemical guide, health information) C: Educational tools	FFQ	FV intake (servings/week)	MD 2·72 (–3·77, 9·21)	8/9
Luten 2016^*^^(73)^	I: Community-based media campaign to promote healthy eating and PA C: Region where no intervention took place	Number of days/week and amount consumed (tool NR)	Fruit intake (g/d)	Partial eta squared (mid-point): –0·01 (NS) Partial eta squared (end of study): –0·10 (NS)	7/9
			Vegetable intake (g/d)	Partial eta squared (mid-point): 0·19 (NS) Partial eta squared (end of study): 0·16 (NS)	
Turk 2016^*^^(55)^	I: ‘Wise Choices’ sessions focused on nutrition and PA, including FV, Ca and fibre intake; portion sizes; USDA MyPlate food choices; step goals using pedometers given. C: Baseline	Seventeen-item nutrition questionnaire	% Who consumed three servings of fruits/d	**+11 %,** ***P *** **= 0·035**	6/9
			% Who consumed three servings of vegetables/d	**+10 %,** ***P *** **= 0·032**	
			% Who consumed three servings of whole grains and high-fibre foods/d	**+10·3 %,** ***P *** **= 0·004**	
			% Who consumed three servings of milk, cheese, and yogurt/d	+12·1 %, *P* = 0·072	
			% Who consumed three 8-oz servings of water/d	+0·1 %, *P* = 0·124	
**Interactive nutrition education interventions**					
Beasley 2019^*^^(47)^	I: Diabetes Prevention Program, including reducing calories and fat, overall healthy eating, PA and managing eating triggers. C: Baseline	4-Day Food Record	Total fruits (servings/d)	MD 0·2 (–0·4, 0·8)	7/9
			Vegetables (servings/d)	MD 0·3 (–0·7, 1·3)	
			Total fat (g/d)	MD –5 (–19, 9)	
			Cholesterol (mg/d)	MD –18 (–49, 85)	
			Na (mg/d)	MD –185 (–986, 616)	
			Total carbohydrate (g/d)	MD 4 (–30, 38)	
			Total protein (g/d)	MD 1 (–16, 18)	
			Vitamin D (mcg/d)	MD 3 (0, 6)	
			Ca (mg/d)	MD –77 (–287, 133)	
			Fe (mg/d)	MD –4 (–8, 0)	
			Potassium (mg/d)	MD 141 (–307, 589)	
			Calories (kJ/d)	MD –133·89 (–1414·19, 1146·42)	
Lara 2015^(61)^	I: Group education including benefits of Mediterranean diet, shopping tips, meal planning. Material package including guidelines, menus, recipes; asked to adopt for 3 weeks. C: Educational group session and package (without menus, recipes or follow up)	3-Day Food Record	Adherence to Mediterranean Diet (9-point score)	MD 0·1 (se 0·3), *P* = 0·721	11/13
			Fish	NS difference	
			FV	NS difference	
			Legumes	NS difference	
			Cereals	NS difference	
			Meat	NS difference	
			Dairy products	NS difference	
**Food access interventions**					
Abusabha 2011^(46)^	I: Veggie Mobile van delivers discounted fresh produce to low-income neighbourhoods C: Baseline	Modified Behavioural Risk Factor Surveillance System survey	FV intake (servings/d)	MD 0·45 (–0·23, 1·14)	3/9
			Fruit (servings/d)	MD –0·23 (–0·74, 0·21)	
			Vegetables (servings/d)	**MD 0·60 (0·07, 1·14)**	
Strout 2017^(53)^	I: ‘GROW’ (Green Organic Vegetable Gardens). Participants given a raised garden bed, ergonomic tools and supplies and chose seeds and recipes. C: Baseline	Mini Nutritional Assessment	Adequate protein intake	% Reporting positive change: 50 % Reporting negative change: 10 (descriptive statistics only)	5/9
			Consumes two or more FV/d	% Reporting positive change: 10 % Reporting negative change: 0 (descriptive statistics only)	
			Consumes 5+ cups of water/d	% Reporting positive change: 30 % Reporting negative change: 0 (descriptive statistics only)	
**Nutrition education with behaviour change techniques and food access interventions**					
Moreau 2015^(49)^	I: Nutrition education and cooking workshops including healthy eating, cancer, CVD prevention, nutrition for aging, labels, fibre, bone health, eating for pleasure, social support, barriers and strategies, recipes, take-home meals. C: Baseline	Abbreviated FFQ	Consumption of recommended portions of food groups (FV, cereal or whole grain, meats and alternatives, water and milk)	**Significant improvement pre-post intervention (** ***P *** **< 0·05, values NR)**	4/9

I, intervention group; C, comparator group; FV, fruits and vegetables; PA, physical activity; MD, mean difference; NR, not reported.

* Asterisks indicate interventions that also included a physical activity component. Bold text indicates statistical significance.

Between and within intervention categories, inconsistent findings were reported. Although the greatest number of studies utilised nutrition education with BCT interventions, findings were mixed. Five interventions found consistently positive changes in food and/or fluid intake^(60,64,66,70,74)^. The Sumida TAKE10 programme (3 months of bi-weekly lectures, take-home activities, monitoring and feedback) (moderate risk of bias)^(60)^, and a 24-week intervention incorporating nutrition education, skill-building activities and planning/implementing behavioural change (low risk of bias)^(66)^ improved both food intake frequency and dietary variety compared with a cross-over control and no-intervention comparator group, respectively. The ‘Eat Smart, Live Strong’ intervention (four weekly interactive nutrition education sessions with goal setting) improved vegetable and fruit intake when compared with a waitlist control (low risk of bias)^(70)^. Tailored nutrition education based on the stages of change with goal setting, action planning, and reinforcement resulted in increased vegetable and fruit consumption after four weekly sessions compared with general health education (high risk of bias)^(64)^. Two modes of delivery of a whole grain education programme (both including skill-building activities and taste testing) increased total and whole-grain intake frequency after three weekly sessions compared with baseline (moderate risk of bias)^(74)^.

Five studies showed improvements in some but not all aspects of food and fluid intake following nutrition education with BCT, as findings were inconsistent across outcomes^(51,52,59,75,76)^. Physical activity and nutrition education with goal setting and skill-building components increased the percentage of participants meeting recommended fruit intake, but not other food groups and macronutrients, as compared to no intervention (low risk of bias)^(59)^. Nutrition education and culturally tailored lifestyle programme incorporating goal setting, action planning and hands-on activities increased the number of participants consuming ≥ 3 meals/d and decreased fried food consumption, but also decreased vegetable intake and found no change in fruit intake as compared to baseline (low risk of bias)^(51)^. Eight weeks of bi-weekly drama-style lectures, food tasting and group discussion improved dietary variety compared to control, but inconsistent findings were noted for macronutrient consumption (low risk of bias)^(75)^. Two studies evaluated the effects of the Texercise Select intervention (10 weeks of twice-weekly education, physical activity, goal setting and action planning). In the first study, Texercise Select increased the likelihood of vegetable and fruit consumption and decreased the likelihood of fast-food intake but did not change soda or water consumption compared with a non-randomised waitlist control (moderate risk of bias)^(76)^; improvements were not sustained at 6-month follow-up. Texercise Select improved vegetable, fruit and water consumption but not soda and fast food consumption compared to baseline in the second study (moderate risk of bias)^(52)^.

Although heterogeneity across interventions was evident, similar nutrition education with BCT interventions was used in four studies (low to high risk of bias) that found no significant changes in food and fluid intake^(56,58,65,69)^. Didactic nutrition education^(55,67,73)^, interactive nutrition education^(47,61)^ and food access^(46,53)^ interventions alone did not appear to change food and fluid intake for the better. Only one study evaluated a nutrition education with BCT and food access (take-home meal portions) intervention and found improved consumption of recommended portions of all food groups (moderate risk of bias)^(49)^.

#### Nutrition risk

Nine studies evaluated the effectiveness of nutrition education with BCT (*n* 7, 78 %)^(48,56,57,62,63,71,72)^, food access (*n* 1, 11 %)^(53)^ and nutrition education with BCT and food access (*n* 1, 11 %)^(68)^ for decreasing nutrition risk. Measures such as the Mini Nutritional Assessment (*n* 3, 33 %)^(53,63,68)^, Dietary Screening Tool (*n* 2, 22 %)^(57,72)^, problematic dietary habits (*n* 2, 22 %)^(48,71)^ and food security (*n* 1, 11 %)^(57)^ were used (Table 3). Heterogeneous interventions and outcomes and inconsistent results were found.

**Table 3:** Nutrition risk (factors impacting food intake) (*n* 9)

Study ID	Description of intervention/comparator	Data collection tool	Outcome	Effect size (95 % CI or sd, *P*-value)	Risk of bias
**Nutrition education with behaviour change technique interventions**					
Francis 2014^*^^(57)^	I: Nutrition and health education including FV and calcium-rich food; PA; safe food handling; food security. Group discussion of smarter goal planning and taste-testing activity. C: Didactic education (newsletters) only	Dietary Screening Tool	Nutrition risk (maximum score 100 where < 60 is ‘at nutrition risk’, 60–75 is ‘possible nutrition risk’ and > 75 is ‘not at nutrition risk’)	**MD 5·35 (0·08, 10·61)**	6/13
		U.S. Household Food Security Survey	Food security (% in each category)	Very low: MD 3·3 (*P* = NR) Low: MD –13·3 (*P* = NR) Marginal/High: MD + 10 (*P* > 0·05) **Overall change in distribution,** ***P *** **< 0·001**	
Hsu 2010^*^^(71)^	I: Nutrition education and practice via dietary choice games (food categories, healthy diet, cooking principles, food recognition), guided by CCAA and NIA materials. PA component (endurance, strength, balance, flexibility) C: No intervention	Problematic dietary habits	Total number of problematic dietary behaviours	NS difference	4/9
			Poor appetite	NS difference	
			Eating alone	NS difference	
			Intake amount change	NS difference	
			Dietary change due to digestive problems	NS difference	
			Dietary change due to oral problems	**Decreased (** ***P *** **< 0·05)**	
			Lack of any categories of food	NS difference	
Lillehoj 2018^*^^(72)^	I: Supplemental Nutrition Assistance Program-Education (SNAP-Ed) including goal setting, recipe tasting, PA break. C: No intervention	Dietary Screening Tool	Nutrition risk (Maximum score 100 where < 60 is ‘at nutrition risk’, 60–75 is ‘possible nutrition risk’ and > 75 is ‘not at nutrition risk’)	**MD (frequent attenders [attended four or more sessions]** ***v*** **. control) 1·69 (sd 15·6),** ***P *** **= 0·04 (controlling for gender and self-efficacy)**	6/9
Manafo 2013^(48)^	I: Nutrition Information Series following Canada’s Food Guide to Healthy Eating; interactive activities including making a food record and reading food labels; healthy snack C: Baseline	Canadian Community Health Survey single item: ‘I consider my eating habits to be…’	Personal eating habits (‘very poor’ to ‘very good’ on 5-point Likert scale)	**MD 0·46,** ***P *** **= 0·046** **Pre: 4·08 (0·51), Post: 4·54 (0·97)**	4/9
Meethien 2011^(62)^	I: Nutrition education for elders and family members. Individual counselling; motivational plan for healthy eating; food preparation activities; training and guidance on meal planning; personal goal setting, behavioural monitoring and maintenance C: Usual care	Elder’s Healthy Eating Scale	Overall healthy eating score (114-item scale, total possible score range 114–570)	**MD (end of study): 147·8 (sd NR),** ***P *** **< 0·001** **MD (follow up): 172·4 (sd NR),** ***P *** **< 0·001**	6/13
Mendoza-Ruvalcaba 2015^*^^(63)^	I: ‘I am Active’ alternating sessions on nutrition or cognitive function; meal planning; goal setting; strength, balance, and mobility physical exercises C: Waitlist control, weekly social activities	Mini Nutritional Assessment	Normal nutritional status (%)	**MD + 31·4 %,** ***P *** **< 0·05**	5/13
			At risk for malnutrition (%)	**MD –31·4 %,** ***P *** **< 0·05**	
Wunderlich 2011^(56)^	I: Elderly Nutrition Program, including education sessions focused on common conditions among older adults (e.g. hypertension and salt intake) and cooking demos. C: Baseline	Nutrition Survey Risk Screening	Nutrition risk score (0–2 good, 3–5 moderate risk, ≥6 high risk)	MD –0·44, *P* = 0·14	3/9
**Food access interventions**					
Strout 2017^(53)^	I: ‘GROW’ (Green Organic Vegetable Gardens). Participants given a raised garden bed, ergonomic tools and supplies, and chose seeds and recipes. C: Baseline	Mini Nutritional Assessment	Self-view of nutritional status	% Reporting positive change: 30 % Reporting negative change: 20 (descriptive statistics only)	5/9
Nutrition education with behaviour change techniques and food access interventions					
Chung 2014^(68)^	I1: Nutrition seminars covering nutrient classification, healthy foods and labelling, recipes, cooking demo. Provided ingredient samples for low cost, nutrient-rich meals with 1-day food samples/week I2: As above with three, 1-day food samples/week	Mini Nutritional Assessment	Nutritional status (< 17 is malnourished, 17–23·5 is at risk of malnutrition, 24–30 is normal nutritional status)	I1: MD 0·9, *P* = 0·641 **I2: MD 2·7,** ***P *** **= 0·019**	7/9

I, intervention group; C, comparator group; FV, fruits and vegetables; PA, physical activity; MD, mean difference; NR, not reported; CCAA, Canadian Center for Activity and Aging; NIA, National Institute of Aging.

* Asterisks indicate interventions that also included a physical activity component.

Bold text indicates statistical significance.

Among seven studies that combined nutrition education with BCT, five demonstrated consistently positive effects^(48,57,62,63,72)^. The ‘I am Active’ intervention (twice weekly nutrition sessions including meal planning and goal setting for two months) increased the percentage of participants with ‘normal’ nutritional status (as defined by the Mini Nutritional Assessment) and decreased the number at risk for malnutrition compared to waitlist control (moderate risk of bias)^(63)^. Compared to didactic education alone, 6-monthly nutrition and health education sessions incorporating goal setting and taste testing decreased nutrition risk (moderate risk of bias)^(57)^. People who frequently attended Supplemental Nutrition Assistant Program-Education (SNAP-Ed) nutrition education sessions with goal setting and recipe tasting decreased their nutrition risk status as compared to control (moderate risk of bias)^(72)^; however, it is important to note that only those attending four or more sessions were included in the analysis. Compared to usual care, weekly nutrition education that incorporated counselling, food preparation, goal setting and behavioural monitoring improved overall healthy eating scores after 3 months (moderate risk of bias)^(62)^. Finally, interactive nutrition education and skill-building activities also improved personal eating habits as compared to baseline (moderate risk of bias)^(48)^.

Conversely, two additional studies that combined nutrition education with BCT did not improve nutritional status (moderate to high risk of bias)^(56,71)^. Two studies (low to moderate risk of bias) evaluated food access or nutrition education with BCT and food access^(53,68)^; these did not consistently reduce nutrition risk.

### Healthy eating knowledge

Five studies reported changes in healthy eating knowledge, generally using study-specific single-item questions (e.g. roles of nutrients, recommended servings) following nutrition education with BCT (*n* 3, 60 %)^(50,74,75)^, nutrition education with BCT and food access (*n* 1, 20 %)^(49)^, and didactic nutrition education (*n* 1, 20 %)^(54)^. Nutrition education with BCT may improve healthy eating knowledge, as found in four studies (low to moderate risk of bias) that incorporated skill-building activities into nutrition education interventions^(49,50,74,75)^ (Table 4).

**Table 4:** Healthy eating knowledge (*n* 5)

Study ID	Description of intervention/comparator	Data collection tool	Outcome	Effect size (95 % CI or sd *, P*-value)	Risk of Bias
**Nutrition education with behaviour change technique interventions**					
MacNab 2017^(74)^	I1: Interactive whole grain nutrition education programme, hands-on activities to identify whole grains, case scenarios to apply knowledge, taste testing, worksheets, handouts, recipes I2: Modified intervention based on delivery style (same activities)	Whole Grain Knowledge Questionnaire	Knowledge of whole grains score (maximum score 31)	**I1: 22·2 (0·4) at post** **I2: 20·3 (0·2) at post** **I1 and I2 combined: 15·1 (4·9) pre to 21·6 (4·0) post,** ***P *** **< 0·001**	5/9
Murayama 2020^(75)^	I: Drama-style lectures on nutrition (protein, fat, carbohydrates) and dietary variety; food tasting; discussion to share knowledge, success and failures; home activities. C: Crossover	Single item ‘I understand the roles of nutrients’	Knowledge score (7-point Likert scale where 1 is ‘disagree’ and 7 is ‘agree’)	**MD 0·69 (0·16, 1·21)**	7/9
		Single item ‘I understand my appropriate amount of food intake’	Knowledge score (7-point Likert scale where 1 is ‘disagree’ and 7 is ‘agree’)	**MD 1·31 (0·63, 1·99)**	
Pogge 2013^*^^(50)^	I: ‘Mindful Choices’ topics included calories, goal setting, building a support system, portion control, PA, nutrition, food labels, stress management. Snacks, tip sheets and calorie counting books provided. C: Baseline	Fifteen-item nutrition knowledge test	Knowledge score (higher score = more knowledge)	**MD 20·3,** ***P *** **< 0·001** **Pre: 61·4 (19·7), Post: 81·7 (19·5)**	5/9
**Nutrition education with behaviour change techniques and food access interventions**					
Moreau 2015^(49)^	I: Nutrition education and cooking workshops including healthy eating, cancer, CVD prevention, nutrition for aging, labels, fibre, bone health, eating for pleasure, social support, barriers and strategies, recipes and take-home meals. C: Baseline	Forty-eight-item questionnaire related to knowledge on nutrition, health and related diseases	Knowledge score (higher score = more knowledge)	**Significant increase pre-post intervention (** ***P *** **< 0·05, values NR)**	4/9
**Didactic nutrition education interventions**					
Thomas 2010^*^^(54)^	I: Educational booklet including nutrition knowledge, recommended food items and PA to improve or prevent chronic diseases C: Baseline	Single item: ‘Do you think health experts recommended that the average American should be eating more or less of these foods?’	Vegetable (% yes)	MD –0·017 (0·247), *P* = 0·367	2/9
			Sugar (% yes)	MD –0·011 (0·280), *P* = 0·594	
			Meat (% yes)	MD –0·028 (0·573), *P* = 0·517	
			Fat (% yes)	MD –0·011 (0·333), *P* = 0·656	
			Fibre (% yes)	MD 0·006 (0·343), *P* = 0·828	
			Fruit (% yes)	MD –0·011 (0·237), *P* = 0·529	
			Salt (% yes)	MD –0·028 (0·247), *P* = 0·132	

I, intervention group; C, comparator group; MD, mean difference; NR, not reported; PA, physical activity.

* Asterisks indicate interventions that also included a physical activity component.

Bold text indicates statistical significance.

### Physical mobility outcomes

#### Physical activity

Physical activity outcomes were assessed in thirteen studies consisting of nutrition education with BCT (*n* 10, 77 %)^(51,52,58–60,65,66,69,71,76)^, didactic nutrition education (*n* 2, 15 %)^(55,73)^ and interactive nutrition education (*n* 1, 8 %)^(47)^ interventions (Table 5). These were captured through both self-reported (e.g. International Physical Activity Questionnaire, 24-hour/7-day recall) and objective measurements (e.g. pedometers, accelerometers). All interventions included a physical activity component either through education or participation during the group-based sessions.

**Table 5:** Physical activity (*n* 13)

Study ID	Description of intervention/comparator	Physical activity co-intervention	Data collection tool	Outcome	Effect size (95 % CI or sd, *P*-value)	Risk of bias
**Nutrition education with behaviour change technique interventions**						
Gallois 2013^(69)^	I: Tools to track FV, dairy products, fish and PA; performance feedback and advice. Standard health info on PA, nutrition and recipes. C: Standard health info and recipes by mail	Education	24-hour recall	Achieving 30 MVPA min/d, I *v*. C at the end of study	Adjusted OR 0·78 (0·51, 1·19)	7/9
Geller 2012^(58)^	I1: Decisional balance sheet for FV intake. Provides basic health knowledge and empowers individuals to consider pros and cons of behaviour adoption. I2: Identical programme targeting PA instead of FV	Education	IPAQ	PA min/d	I1: MD 47·05 (79·77), descriptive statistics only I2: MD 32·19 (47·34), descriptive statistics only	5/13
Hsu 2010^(71)^	I: Nutrition education and practice via dietary choice games (food categories, healthy diet, cooking principles, food recognition), guided by CCAA and NIA materials. PA component (endurance, strength, balance, flexibility) C: No intervention	Participation	Regular exercise behaviour (tool NR)	% Doing exercise for at least 30 min, 3×/week (yes/no)	**Increased (** ***P *** **< 0·001)**	4/9
Jancey 2017^(59)^	I: PA and nutrition education (e.g. goal setting, monitoring and feedback; skill building; social support; exercise demo); educational resources (booklet, calendar, exercise chart, resistance bands, newsletters); motivational interviewing (goal setting, adherence, sustainability) C: No intervention	Education	IPAQ	Walking time (min/week)	MD –16·57 (–83·48, 50·34)	10/13
				Sitting time (min/week)	MD –211·6 (–457·59, 34·39)	
				Moderate activity (min/week)	**MD 71·09 (15·7, 126·48)**	
				Vigorous activity: % participating > 10 min	MD –2·7 %, *P* = 0·716	
				Strength exercise: % participating > 10 min	**MD 22·4 %,** ***P *** **= 0·002**	
Kimura 2013^(60)^	I: ‘Sumida TAKE10’ program. Lecture on good dietary habits; participants self-monitored dietary check sheets during lecture and received instructor feedback; stretching and strengthening exercise. C: Crossover	Participation	Physical activity questionnaire (tool NR)	Days of walking or exercise/week	No difference within or between groups (*P* > 0·05)	7/13
Schwingel 2017^(51)^	I: Nutrition education and culturally tailored lifestyle change curriculum, including healthy living, healthy eating, nutrition labels, buying healthy food, stress management, barriers, goal setting, action plans, home activities (e.g. healthy meal prep, grocery shopping with grandchildren, PA, pedometer step-tracking), motivational telephone calls C: Baseline	Participation	Accelerometers	MVPA (min/week)	NS difference	7/9
				% Meeting MVPA guidelines	% (end of study): +20·4 % % (follow up): +28·1 % *P* = 0·08 (linear trend)	
Silva-Smith 2013^(65)^	I: ‘Promoting Older Adult Wellness’, education, social network, motivational support and short/long term goal setting (for PA and DASH diet); supervised, progressive walking programme C: Attention control health newsletters	Participation	7-day pedometer	Steps/week	Adjusted MD 4573·24 (90 % CI: –259·37, 9405·86)	8/13
			7-day self-report	PA (units NR)	**Adjusted MD 48·17 (90 % CI: 5·77, 90·58)**	
Smith 2015^(52,95)^	I: Texercise Select. Education on healthy dietary habits and cooking; PA and nutrition logs; goal setting, action plans and group brainstorming; PA component incorporating flexibility, strength, balance, endurance. C: Baseline	Participation	RAPA-1	Aerobic PA score (Range 1–5, 5 = highest)	**MD 0·65,** ***P *** **< 0·001**	6/9
			RAPA-2	Participation in strength training (post *v*. pre)	**OR 4·04,** ***P *** **< 0·001**	
				Participation in flexibility training (post *v*. pre)	**OR 5·48,** ***P *** **< 0·001**	
Smith 2020^(76,96)^	I: Texercise Select. Education on healthy dietary habits and cooking; PA and nutrition logs; goal setting, action plans and group brainstorming; PA component incorporating flexibility, strength, balance, endurance. C: Usual care, waitlist control	Participation	IPAQ	Sedentary (h/d)	Adjusted MD (end of study): –0·77 (–1·63, 0·09) **Adjusted MD (follow up): –1·1 (–2·13, –0·07)**	6/9
				Light PA (min/week)	Adjusted MD (end of study): 27·24 (–35·96, 90·44) **Adjusted MD (follow up): 90·36 (15·07, 165·65)**	
				Moderate PA (min/week)	**Adjusted MD (end of study): 44·95 (11·59, 78·31)** **Adjusted MD (follow up): 59·94 (20·59, 99·29)**	
				Vigorous PA (min/week)	Adjusted MD (end of study): 14·36 (–3·58, 32·3) **Adjusted MD (follow up): 31·67 (10·41, 52·93)**	
Uemura 2018^(66)^	I: Educational health promotion on exercise, diet, nutrition, cognitive activity including malnutrition, food labelling, walking, resistance exercise. PA self-monitored via accelerometer. Self-planning for and implementing behavioural change C: No intervention	Participation	Accelerometer	Steps/d	**MD 1674 (se 452),** ***P *** **< 0·001**	10/13
				PA level (units NR)	**MD 0·05 (se 0·02),** ***P *** **= 0·01**	
**Didactic nutrition education interventions**						
Luten 2016^(73)^	I: Community-based media campaign to promote healthy eating and PA C: Region where no intervention took place	Education	Short QUestionnaire to ASsess Health-enhancing physical activity (SQUASH)	Total PA	Partial eta squared (end of study): –0·03 (NS)	7/9
				Transport-related PA	Partial eta squared (end of study): **0·38,** ***P *** **< 0·01**	
				Household-related PA	Partial eta squared (end of study): –0·10 (NS)	
				Leisure-time PA	Partial eta squared (end of study): –0·08 (NS)	
Turk 2016^(55)^	I: ‘Wise Choices’ sessions focused on nutrition and PA, including FV, Ca and fibre intake; portion sizes; USDA MyPlate food choices; step goals using pedometers given. C: Baseline	Participation	9-item PA questionnaire	Moderate activity (h/week)	Weekday: no difference (*P* = 0·99) Weekend: no difference (*P* = 0·83)	6/9
				Vigorous activity (h/week)	Weekday: no difference (*P* = 0·90) Weekend: no difference (*P* = 0·37)	
				City blocks walked/d	**Pre: Median 3 (Range: 0–36), Post: Median 6 (Range: 0–90),** ***P *** **< 0·001**	
			Pedometers	Steps/d	**Pre: Median 3143 (Range: 274–10 593), Post: Median 3480 (Range: 662–18 592),** ***P *** **< 0·001**	
**Interactive nutrition education interventions**						
Beasley 2019^(47)^	I: Diabetes Prevention Program, including reducing calories and fat, overall healthy eating, PA and managing eating triggers. C: Baseline	Education	CHAMP	Moderate PA (min/week)	MD 66 (–178, 310)	7/9
				All PA (min/week)	MD 127 (–264, 518)	
			PA trackers (Fitbit)	Lightly active (min/week)	MD –10 (–55, 35)	
				Fairly active (min/week)	MD –2 (–13, 9)	
				Very active (min/week)	MD –3 (–4, –2)	
				Total activity (min/week)	MD –15 (–76, 46)	

I, intervention group; FV, fruits and vegetable; PA, physical activity;. C, comparator group; IPAQ, International Physical Activity Questionnaire short form; MD, mean difference; CCAA, Canadian Centre for Activity and Aging; NIA, National Institute of Aging; MVPA, moderate to vigorous intensity physical activity; NR, not reported; NS, not significant; RAPA, Rapid Assessment of Physical Activity; CHAMP, Cardiovascular Healthy Activities Model Program for Seniors. Bold text indicates statistical significance.

Across ten studies evaluating nutrition education with BCT, findings were mixed. Four studies found a consistent increase, including participation in regular exercise^(71)^ aerobic/strength training^(52)^, steps per day^(66)^ and time spent in light, moderate or vigorous physical activity^(76)^ (low to moderate risk of bias); each of these included physical activity participation within group-based sessions. Six other studies (low to moderate risk of bias) did not report consistent improvements, with three studies including physical activity education only^(58,59,69)^ and three^(51,60,65)^ including physical activity participation. Didactic nutrition education^(55,73)^ and interactive nutrition education^(47)^ interventions alone did not appear to increase physical activity.

#### Functional outcomes

Five studies reported the impact of nutrition education with BCT (*n* 4, 80 %)^(52,63,66,71)^ and didactic nutrition education (*n* 1, 20 %)^(55)^ on functional mobility (Table 6). Although heterogeneous intervention and outcome types were again noted, both nutrition education with BCT and didactic nutrition education generally improved functional outcomes (e.g. Timed Up and Go, gait speed), as noted in four studies (low to moderate risk of bias)^(52,55,63,66)^. Each of these also included participation in physical activity as a co-intervention.

**Table 6:** Functional outcomes (*n* 5)

Study ID	Description of intervention/comparator	Physical activity co-intervention	Data collection tool	Outcome	Effect size (95 % CI or sd, *P*-value)	Risk of bias
**Nutrition education with behaviour change technique interventions**						
Hsu 2010^(71)^	I: Nutrition education and practice via dietary choice games (food categories, healthy diet, cooking principles, food recognition), guided by CCAA and NIA materials. PA component (endurance, strength, balance and flexibility) C: No intervention	Participation	Elderly Functional Index	ADL difficulty	NS difference	4/9
				Total physical function difficulty	NS difference	
Mendoza-Ruvalcaba 2015^(63)^	I: ‘I am Active’ alternating sessions on nutrition or cognitive function; meal planning; goal setting; strength, balance and mobility physical exercises C: Waitlist control, weekly social activities	Participation	Tinetti scale	Risk of falls	**I: Cohen’s** ***d*** **(end of study): 0·34 (** ***P *** **< 0·05);** Cohen’s *d* (follow-up): 0·24 (NS) C: Cohen’s *d* (end of study): 0·02 (NS); Cohen’s *d* (follow-up): 0·21 (NS)	5/13
				Balance	**I: Cohen’s** ***d*** **(end of study): 0·41 (** ***P *** **< 0·05);** Cohen’s d (follow-up): 0·01 (NS) **C: Cohen’s** ***d*** **(end of study) 0·01 (** ***P *** **< 0·05);** Cohen’s d (follow-up): 0·12 (NS)	
				Gait	I: Cohen’s *d* (end of study): 0·16 (NS); Cohen’s *d* (follow-up): 0·48 (NS) C: Cohen’s *d* (end of study): 0·33 (NS); Cohen’s *d* (follow-up): 0·29 (NS)	
			Goniometer	Flexibility	**I: Cohen’s** ***d*** **(end of study): 0·65 (** ***P *** **< 0·05);** Cohen’s *d* (follow up): 0·07 (NS) C: Cohen’s *d* (end of study): 0·01 (NS); Cohen’s *d* (follow-up): 0·22 (NS)	
			Hand-held dynamometer	Grip strength (right)	I: Cohen’s *d* (end of study): 0·08 (NS); Cohen’s *d* (follow-up): 0·20 (NS) C: Cohen’s *d* (end of study): 0·03 (NS); Cohen’s *d* (follow-up): 0·10 (NS)	
				Grip strength (left)	I: Cohen’s *d* (end of study): 0·14 (NS); Cohen’s *d* (follow-up): 0 (NS) C: Cohen’s *d* (end of study): 0·04 (NS); Cohen’s *d* (follow-up): 0·03 (NS)	
Smith 2015^(52,95)^	I: Texercise Select. Education on healthy dietary habits and cooking; PA and nutrition logs; goal setting, action plans and group brainstorming; PA component incorporating flexibility, strength, balance and endurance. C: Baseline	Participation	–	TUG (s)	**MD –1·5,** ***P *** **< 0·01** **Pre: 13·03 (5·19), Post: 11·53 (4·38)**	6/9
Uemura 2018^(66)^	I: Educational health promotion on exercise, diet, nutrition and cognitive activity including malnutrition, food labelling, walking and resistance exercise. PA self-monitored via accelerometer. Self-planning for and implementing behavioural change C: No intervention	Participation	–	TUG (s)	**MD –0·84 (se 0·18),** ***P *** **< 0·001**	10/13
			5-m walking test	Gait speed (m/s)	**MD 0·18 (se 0·04),** ***P *** **< 0·001**	
			Dynamometer	Grip strength (kg)	MD 0·99 (se 0·59), *P* = 0·09	
**Didactic nutrition education interventions**						
Turk 2016^(55)^	I: ‘Wise Choices’ sessions focused on nutrition and PA, including FV, Ca and fibre intake; portion sizes; USDA MyPlate food choices; step goals using pedometers given. C: Baseline	Participation	–	TUG (s)	**Median –0·9 (range: –23·4–9·7),** ***P *** **< 0·001**	6/9
			Nine-item PA questionnaire	Self-reported walking pace (mph)	**Significant change in proportion of participants who increased pace,** ***P *** **= 0·009**	

I, intervention group; ADL, activities of daily living; C, comparator group; CCAA, Canadian Centre for Activity and Aging; NIA, National Institute of Aging; PA, physical activity; TUG, Timed Up and Go test; MD, mean difference; NS, not significant; FV, fruits and vegetable. Bold text indicates statistical significance.

## Discussion

Given the wide heterogeneity and inconsistent findings across this body of literature, our certainty in the effectiveness of group-based community nutrition interventions to improve food and fluid intake, nutritional status, healthy eating knowledge and measures of physical activity or physical function in older adults is low. The available evidence suggests that nutrition education with BCT may be the most promising approach to improving food and fluid intake, nutritional status and healthy eating knowledge. Given the variation across interventions and outcomes, it is unclear which intervention is optimal for implementation in community-based settings. Both intervention duration and frequency varied widely across studies, with no discernable patterns to suggest a minimally or optimally effective intervention ‘dose’. While one would suspect that longer programmes or more frequent sessions would have a greater impact, this did not appear to be the case in the studies included in this review. Overall, these conclusions should be interpreted with caution related to high variability among intervention components and outcome measurements, in addition to unclear to high risk of bias within the studies themselves.

Most of the interventions combined nutrition education with BCT. Although we broadly grouped interventions as either including BCT or not, we did not explicitly code these based on the BCT Taxonomy^(77)^ to identify the discrete strategies used. The effectiveness of nutrition education with BCT, particularly concerning food and fluid intake and nutrition risk, remains unclear; there is a lack of evidence on which specific BCT are required to elicit significant change. Given wide heterogeneity across intervention components, duration, frequency, interventionists, locations and theoretical frameworks used, we could not distinguish any noticeable patterns among nutrition education with BCT interventions that were consistently effective *v*. those that were not. Interventions that described nutrition education with BCT appeared to be more intensive than interventions that focussed on didactic or interactive nutrition education alone. However, it is conceivable that individuals who consent to participate in a more intensive programme could perhaps be more committed to overall behavioural change. Appropriately selecting and evaluating the effectiveness of BCT remains an emerging area of inquiry^(78)^; thus, understanding the most relevant and effective BCT to improve nutrition and mobility outcomes among community-dwelling older adults is an important next step. More fulsome reporting of intervention components following definitions from the BCT Taxonomy^(77)^ or using a recognised framework such as the TIDIeR checklist^(34)^ would allow future exploration of key intervention components.

We explored physical activity and functional outcomes gave the established link between adequate nutritional intake and mobility in older adults; however, all studies that explored mobility outcomes also included a physical activity co-intervention. The existence of a co-intervention made it difficult to determine which component(s) of these multifaceted interventions were driving change when observed. Although we hypothesised that comprehensive healthy lifestyle programmes might have a greater impact on behavioural change overall, we did not observe any clear trends to indicate whether the interventions that included both nutrition and physical activity components were more effective for either nutrition or mobility-related outcomes than those focussed on nutrition alone (Table 2–6). There is limited available evidence regarding the effectiveness of single *v*. multiple health behaviour change interventions in older adults^(79)^, highlighting a potential area for further investigation^(80)^.

Given the complex factors (e.g. financial, environmental, cultural) known to impact older adults’ ability to maintain a healthy diet^(14)^, it is important to recognise that while nutrition education and skill building may be effective at increasing healthy eating knowledge and intentions, they may be insufficient to change outcomes such as food and fluid intake or nutrition risk. Using an equity lens, we assessed the nine studies included in this review that explicitly targeted populations with low socio-economic status (e.g. recruitment from low-income housing). Overall, findings were inconsistent, with improvements following education with BCT noted in some but not others. This may not be surprising if the primary barriers to quality food intake (e.g. vegetable and fruit consumption) are cost or ease of access^(9)^. Environmental support and policy-level public health interventions are likely needed to ensure equitable access to healthy food before nutrition education and skill building can be expected to make a meaningful difference^(81–83)^.

To our knowledge, this review is the first to systematically identify, appraise and synthesise evidence regarding the effectiveness of nutrition-focused group-based interventions targeting food and fluid intake, nutrition risk and mobility outcomes in community-dwelling older adults. However, our results are consistent with recommendations from a pair of evidence syntheses and an expert commentary published in 2003 that concluded nutrition education alone was insufficient to improve nutritional status among older adults^(24,84,85)^. In line with our findings, the authors recommended that education be paired with behaviour change strategies and community participation to enhance programme effectiveness. Similarly, a 2007 review of Canadian research highlighted successful components of community nutrition programmes for older adults, including cooking classes, recipe exchanges, counselling, social support and engagement, motivation and interactivity^(86)^. Consistent with our findings, these strategies would also be considered techniques to support behaviour change.

Several important considerations should be made while interpreting the findings from this review. Although our search strategy was comprehensive, it was restricted to studies published in English since 2010, which may be a limitation. However, our results are consistent with findings from older, related reviews described above that considered single studies dating back to 1993^(24,84–86)^. Further, despite the updated Consolidated Standards of Reporting Trials (CONSORT) 2010 guidelines^(87)^, methodological and reporting challenges contributed to the unclear to high risk of bias in the studies included in this review. Therefore, it is unlikely that studies published before 2010 would be of higher methodological quality or change our overall conclusions. Given that the aim of this review was to explore the effectiveness of group-based interventions, it was appropriate to focus on intervention studies only. Qualitative data may highlight important insights into reasons for variable intervention effectiveness (e.g. implementation insights). While we did include two mixed-methods studies, only quantitative data were extracted. Further, although we did endeavour to integrate considerations about study quality, consistency and directness throughout the wide variability in outcomes across included studies limited us from applying a formal approach, such as GRADE^(88)^ to assess certainty in this body of evidence.

Our conclusions are also limited by the nature of the primarily quasi-experimental single studies with incomplete follow-up included within the review. We did not observe any differences in the types of interventions or findings among the studies that reported > 20 % attrition. The large dropout rate observed might be attributed to the population; researchers often face difficulties recruiting and retaining older adults in research due to health and mobility challenges among this population^(89)^. When considering intervention context, it is also possible that participation may have been fluid because of the nature of delivery in settings such as congregate meal sites and seniors’ centres that may operate on a drop-in basis. Lack of reliable outcome measurement tools may explain some of the inconsistency across studies. Challenges associated with measuring the impact of community nutrition programmes have previously been documented^(90)^; given the nature of self-reported data, outcomes such as food intake, dietary behaviour and knowledge are notoriously complex constructs to measure accurately. Despite previous calls for community nutrition interventions for older adults based on behaviour change theories^(85)^, less than half of the studies in this review used a theoretical framework to inform intervention delivery; this might further explain some of the variability noted in our results. We also observed variability in the content of the nutrition education provided across interventions. It is unclear if recommendations were consistently based on current, evidence-based healthy eating guidelines for older adults, further explaining the inconsistent effectiveness observed.

### Implications for research

More studies using RCT designs are needed to increase confidence in the impact of group-based community nutrition interventions. Although blinding of participants and interventionists is nearly impossible given the nature of the interventions, future studies should strive to blind outcome assessors and data analysts to enhance internal validity. Authors using quasi-experimental approaches should include control groups to facilitate stronger comparisons. In an attempt to overcome potential attrition bias due to incomplete follow up with older adult participants in community settings, future studies may consider strategies such as providing transportation and involving older adults/community providers during intervention planning to ensure issues that may lead to decreased retention are considered and addressed^(89)^. Given that community-based nutrition programming tends to be delivered via public health initiatives and not always through funded programmes of research, challenges noted with intervention design, outcome assessment, study quality and inappropriate statistical analyses might be attributed to the probable lack of resources available to support community programme development and evaluation. Prioritising research funding to support the development and evaluation of community-based nutrition programmes for older adults is necessary to improve the quality of the evidence base.

### Implications for practice

For organisations looking to design and implement community-based nutrition programming for older adults, nutrition education with embedded BCT (e.g. goal setting, hands-on skill-building activities, taste testing) demonstrated the most promise to improve healthy eating outcomes. However, there is wide heterogeneity in the available evidence, including programme length and session frequency. The discrete techniques and intervention components that might be most important to include have yet to be determined. These will likely need to be tailored based on the needs and preferences of the community and local context. Future programme design should be based on recognised theories of behaviour change. There is a potential to draw upon significant recent advancements in behaviour change theory^(91,92)^, which have been applied in developing complex interventions for healthy eating^(93,94)^.

## Conclusion

Group-based nutrition education with BCT demonstrated the most promise in improving food and fluid intake, nutritional status and healthy eating knowledge among community-dwelling older adults. The impact of these programmes on mobility outcomes is less clear. These findings should be interpreted with caution, given the generally unclear to high risk of bias and low quality, heterogeneous evidence base. We have highlighted several key takeaways regarding how the quality of this body of literature could be improved. Future group and community-based programmes should use recognised behavioural change theories to develop and implement evidence-based nutrition education with skill-building activities to improve healthy eating among older adults.

## Supplementary material

For supplementary material accompanying this paper visit https://doi.org/10.1017/S136898002200115X. click here to view supplementary material

## References

[1] United Nations (2020) World Population Ageing 2020 Highlights. https://www.un.org/development/desa/pd/sites/www.un.org.development.desa.pd/files/undesa_pd-2020_world_population_ageing_highlights.pdf (accessed June 2021).

[2] Barnett K , Mercer SW , Norbury M et al. (2012) Epidemiology of multimorbidity and implications for health care, research, and medical education: a cross-sectional study. Lancet 380, 37–43. 22579043 10.1016/S0140-6736(12)60240-2

[3] Raina P , Gilsing A , Mayhew AJ et al. (2020) Individual and population level impact of chronic conditions on functional disability in older adults. PLOS ONE 15, e0229160. 32078637 10.1371/journal.pone.0229160 PMC7032687

[4] Bassim C , Mayhew AJ , Ma J et al. (2020) Oral health, diet, and frailty at baseline of the canadian longitudinal study on aging. J Am Geriatr Soc 68, 959–966. 32162690 10.1111/jgs.16377

[5] Raina P , Ali MU , Joshi D et al. (2021) The combined effect of behavioural risk factors on disability in aging adults from the Canadian Longitudinal Study on Aging (CLSA). Prev Med 149, 106609. 33984371 10.1016/j.ypmed.2021.106609

[6] Government of Canada (2021) Canada’s Food Guide: Healthy Eating for Seniors. https://food-guide.canada.ca/en/tips-for-healthy-eating/seniors/ (accessed June 2021).

[7] U.S. Department of Agriculture (2020) Dietary Guidelines for Americans, 2020–2025. https://www.dietaryguidelines.gov/sites/default/files/2021-03/Dietary_Guidelines_for_Americans-2020-2025.pdf (accessed June 2021).

[8] Leslie W & Hankey C (2015) Aging, nutritional status and health. Healthcare 3, 648–658. 27417787 10.3390/healthcare3030648 PMC4939559

[9] Choi YJ , Crimmins EM , Kim JK et al. (2021) Food and nutrient intake and diet quality among older Americans. Public Health Nutr 24, 1638–1647. 33557974 10.1017/S1368980021000586 PMC8094430

[10] Wakimoto P & Block G (2001) Dietary intake, dietary patterns, and changes with age: an epidemiological perspective. J Gerontol A Biol Sci Med Sci 56, 65–80. 11730239 10.1093/gerona/56.suppl_2.65

[11] Kaur D , Rasane P , Singh J et al. (2019) Nutritional interventions for elderly and considerations for the development of geriatric foods. Curr Aging Sci 12, 15–27. 31109282 10.2174/1874609812666190521110548 PMC6971894

[12] Conklin AI , Forouhi NG , Surtees P et al. (2014) Social relationships and healthful dietary behaviour: evidence from over-50s in the EPIC cohort, UK. Soc Sci Med 100, 167–175. 24035440 10.1016/j.socscimed.2013.08.018 PMC3969105

[13] Vesnaver E & Keller HH (2011) Social influences and eating behavior in later life: a review. J Nutr Gerontol Geriatr 30, 2–23. 23286638 10.1080/01639366.2011.545038

[14] Keller HH , Dwyer JJM , Senson C et al. (2007) A social ecological perspective of the influential factors for food access described by low-income seniors. J Hunger Environ Nutr 1, 27–44.

[15] Webber SC , Porter MM & Menec VH (2010) Mobility in older adults: a comprehensive framework. Gerontologist 50, 443–450. 20145017 10.1093/geront/gnq013

[16] Schwartz N , Buliung R & Wilson K (2019) Disability and food access and insecurity: a scoping review of the literature. Health Place 57, 107–121. 31026771 10.1016/j.healthplace.2019.03.011

[17] Morley JE , Abbatecola AM , Argiles JM et al. (2011) Sarcopenia with limited mobility: an international consensus. J Am Med Dir Assoc 12, 403–409. 21640657 10.1016/j.jamda.2011.04.014 PMC5100674

[18] Robinson S , Granic A & Sayer AA (2019) Nutrition and muscle strength, as the key component of sarcopenia: an overview of current evidence. Nutrients 11, 2942. 31817048 10.3390/nu11122942 PMC6950468

[19] Cruz-Jentoft AJ , Kiesswetter E , Drey M et al. (2017) Nutrition, frailty, and sarcopenia. Aging Clin Exp Res 29, 43–48. 28155181 10.1007/s40520-016-0709-0

[20] Milaneschi Y , Tanaka T & Ferrucci L (2010) Nutritional determinants of mobility. Curr Opin Clin Nutr Metab Care 13, 625–629. 20736822 10.1097/MCO.0b013e32833e337d PMC2964392

[21] Hopewell S , Adedire O , Copsey BJ et al. (2018) Multifactorial and multiple component interventions for preventing falls in older people living in the community. Cochrane Database Syst Rev 7, CD012221. 30035305 10.1002/14651858.CD012221.pub2 PMC6513234

[22] Webb GP (2019) Nutrition: Maintaining and Improving Health. Boca Raton: CRC Press.

[23] Srivarathan A , Jensen AN & Kristiansen M (2019) Community-based interventions to enhance healthy aging in disadvantaged areas: perceptions of older adults and health care professionals. BMC Health Serv Res 19, 7. 30611262 10.1186/s12913-018-3855-6 PMC6321658

[24] Higgins MM & Clarke Barkley M (2004) Group nutrition education classes for older adults. J Nutr Elder 23, 67–98. 15149942 10.1300/J052v23n04_06

[25] Manilla B , Keller HH & Hedley MR (2010) Food tasting as nutrition education for older adults. Can J Diet Pract Res 71, 99–102. 20525423 10.3148/71.1.2010.99

[26] Keller HH , Hedley M , Hadley T et al. (2005) Food workshops, nutrition education, and older adults: a process evaluation. J Nutr Elder 24, 5–23. 10.1300/J052v24n03_03 15911522

[27] Agronin M (2009) Group therapy in older adults. Curr Psychiatr Rep 11, 27–32. 10.1007/s11920-009-0005-1 19187705

[28] Keller HH , Gibbs A , Wong S et al. (2004) Men can cook! Development, implementation, and evaluation of a senior men’s cooking group. J Nutr Elder 24, 71–87. 15339722 10.1300/J052v24n01_06

[29] Keller HH , Hedley MR , Wong SS et al. (2006) Community organized food and nutrition education: participation, attitudes and nutritional risk in seniors. J Nutr Health Aging 10, 15–20. 16453053

[30] Neil-Sztramko SE , Teggart K , Moore C et al. (2021) Community-Based Physical Activity and/or Nutrition Interventions to Promote Mobility in Older Adults: an Umbrella Review (PREPRINT (Version 2). 10.21203/rs.3.rs-578194/v2 (accessed September 2021). PMC10549072 36944044

[31] Page MJ , McKenzie JE , Bossuyt PM et al. (2021) The PRISMA 2020 statement: an updated guideline for reporting systematic reviews. BMJ 372, 1–9. 10.1136/bmj.n71 PMC8005924 33782057

[32] Keller HH , Goy R & Kane SL (2005) Validity and reliability of SCREEN II (Seniors in the community: risk evaluation for eating and nutrition, Version II). Eur J Clin Nutr 59, 1149–1157. 16015256 10.1038/sj.ejcn.1602225

[33] JBI (2021) Critical Appraisal Tools. https://jbi.global/critical-appraisal-tools (accessed June 2021).

[34] Hoffmann TC , Glasziou PP , Boutron I et al. (2014) Better reporting of interventions: template for intervention description and replication (TIDieR) checklist and guide. BMJ 348, 1–12. 10.1136/bmj.g1687 24609605

[35] Tufanaru C , Munn Z , Aromataris E et al. (2020) Systematic Reviews of Effectiveness. Aromataris. JBI Manual for Evidence Synthesis. 10.46658/JBIMES-20-04 (accessed June 2021).

[36] Higgins JPT , Li T & Deeks JJ (2021) Choosing Effect Measures and Computing Estimates of Effect. Cochrane Handbook for Systematic Reviews of Interventions Version 6.2. Weinheim: Wiley; available at http://www.training.cochrane.org/handbook (accessed February 2021).

[37] The Cochrane Collaboration (2014) Review Manager (RevMan) (Computer Program). Copenhagen: The Nordic Cochrane Centre.

[38] Institute of Medicine (US) (2021) Committee on Dietary Risk Assessment in the WIC Program (2002) Dietary Risk Assessment in the WIC Program: Food-Based Assessment of Dietary Intake. https://www.ncbi.nlm.nih.gov/books/NBK220560/ (accessed June 2021).

[39] Torheim LE , Barikmo I , Parr CL et al. (2003) Validation of food variety as an indicator of diet quality assessed with a food frequency questionnaire for Western Mali. Eur J Clin Nutr 57, 1283–1291. 14506490 10.1038/sj.ejcn.1601686

[40] Leon Guerrero RT , Chong M , Novotny R et al. (2015) Relative validity and reliability of a quantitative food frequency questionnaire for adults in Guam. Food Nutr Res 59, 26276. 25947296 10.3402/fnr.v59.26276 PMC4422845

[41] Cade JE , Burley VJ , Warm DL et al. (2004) Food-frequency questionnaires: a review of their design, validation and utilisation. Nutr Res Rev 17, 5–22. 19079912 10.1079/NRR200370

[42] Cade J , Thompson R , Burley V et al. (2002) Development, validation and utilisation of food-frequency questionnaires – a review. Public Health Nutr 5, 567–587. 12186666 10.1079/PHN2001318

[43] Cleland C , Ferguson S , Ellis G et al. (2018) Validity of the International Physical Activity Questionnaire (IPAQ) for assessing moderate-to-vigorous physical activity and sedentary behaviour of older adults in the United Kingdom. BMC Med Res Methodol 18, 176–176. 30577770 10.1186/s12874-018-0642-3 PMC6303992

[44] Tomioka K , Iwamoto J , Saeki K et al. (2011) Reliability and validity of the International Physical Activity Questionnaire (IPAQ) in elderly adults: the Fujiwara-kyo Study. J Epidemiol 21, 459–465. 21946625 10.2188/jea.JE20110003 PMC3899462

[45] Topolski TD , LoGerfo J , Patrick DL et al. (2006) The Rapid Assessment of Physical Activity (RAPA) among older adults. Prev Chronic Dis 3, A118–A118. 16978493 PMC1779282

[46] Abusabha R , Namjoshi D & Klein A (2011) Increasing access and affordability of produce improves perceived consumption of vegetables in low-income seniors. Am Dietetic Assoc 111, 1549–1555. 10.1016/j.jada.2011.07.003 21963022

[47] Beasley JM , Kirshner L , Wylie-Rosett J et al. (2019) BRInging the Diabetes prevention program to GEriatric populations (BRIDGE): a feasibility study. Pilot Feasibility Stud 5, 129. 31741744 10.1186/s40814-019-0513-7 PMC6849183

[48] Manafo E , Jose K & Silverberg D (2013) Promoting nutritional well-being in seniors: feasibility study of a nutrition information series. Can J Diet Pract Res 74, 175–180. 24472165 10.3148/74.4.2013.175

[49] Moreau M , Plourde H , Hendrickson-Nelson M et al. (2015) Efficacy of nutrition education-based cooking workshops in community-dwelling adults aged 50 years and older. J Nutr Gerontol Geriatr 34, 369–387. 26571355 10.1080/21551197.2015.1084257

[50] Pogge EK & Eddings L (2013) Effect of a 12-week nutrition and wellness program in independent living seniors. J Nutr Educ Behav 45, 471–472. 23478170 10.1016/j.jneb.2013.01.014

[51] Schwingel A , Galvez P , Linares D et al. (2017) Using a mixed-methods RE-AIM framework to evaluate community health programs for older Latinas. J Aging Health 29, 551–593. 27079919 10.1177/0898264316641075

[52] Smith ML , Ory MG , Jiang L et al. (2015) Texercise select effectiveness: an examination of physical activity and nutrition outcomes. Transl Behav Med 5, 433–442. 26622916 10.1007/s13142-014-0299-3 PMC4656227

[53] Strout K , Jemison J , O’Brien L et al. (2017) GROW: green organic vegetable gardens to promote older adult wellness: a feasibility study. J Community Health Nurs 34, 115–125. 28767290 10.1080/07370016.2017.1340554

[54] Thomas L , Almanza B & Ghiselli R (2010) Nutrition knowledge of rural older populations: can congregate meal site participants manage their own diets? J Nutr Elder 29, 325–344. 20711926 10.1080/01639366.2010.500951

[55] Turk MT , Elci OU , Resick LK et al. (2016) Wise choices: nutrition and exercise for older adults: a community-based health promotion intervention. Fam Community Health 39, 263–272. 27536931 10.1097/FCH.0000000000000116

[56] Wunderlich S , Bai Y & Piemonte J (2011) Nutrition risk factors among home delivered and congregate meal participants: need for enhancement of nutrition education and counseling among home delivered meal participants. J Nutr Health Aging 15, 768–773. 22089226 10.1007/s12603-011-0090-9 PMC12878094

[57] Francis SL , MacNab L & Shelley M (2014) A theory-based newsletter nutrition education program reduces nutritional risk and improves dietary intake for congregate meal participants. J Nutr Gerontol Geriatr 33, 91–107. 24827061 10.1080/21551197.2014.906336

[58] Geller KS , Mendoza ID , Timbobolan J et al. (2012) The decisional balance sheet to promote healthy behavior among ethnically diverse older adults. Public Health Nurs 29, 241–246. 22512425 10.1111/j.1525-1446.2011.00987.x PMC3334875

[59] Jancey J , Holt A-M , Lee A et al. (2017) Effects of a physical activity and nutrition program in retirement villages: a cluster randomised controlled trial. Int J Behav Nutr Phys Act 14, 92. 28697803 10.1186/s12966-017-0543-6 PMC5504569

[60] Kimura M , Moriyasu A , Kumagai S et al. (2013) Community-based intervention to improve dietary habits and promote physical activity among older adults: a cluster randomized trial. BMC Geriatr 13, 8. 23343312 10.1186/1471-2318-13-8 PMC3560222

[61] Lara J , Turbett E , McKevic A et al. (2015) The Mediterranean diet among British older adults: its understanding, acceptability and the feasibility of a randomised brief intervention with two levels of dietary advice. Maturitas 82, 387–393. 26316025 10.1016/j.maturitas.2015.07.029

[62] Meethien N , Pothiban L , Ostwald SK et al. (2011) Effectiveness of nutritional education in promoting healthy eating among elders in northeastern Thailand. Pac Rim Int J Nurs Res Thail 15, 188–201.

[63] Mendoza-Ruvalcaba NM & Arias-Merino ED (2015) ‘I am active’: effects of a program to promote active aging. Clin Interv Aging 10, 829–837. 26005337 10.2147/CIA.S79511 PMC4427596

[64] Salehi L , Mohammad K & Montazeri A (2011) Fruit and vegetables intake among elderly Iranians: a theory-based interventional study using the five-a-day program. Nutr J 10, 123. 22078240 10.1186/1475-2891-10-123 PMC3247847

[65] Silva-Smith AL , Fleury J & Belyea M (2013) Effects of a physical activity and healthy eating intervention to reduce stroke risk factors in older adults. Prev Med 57, 708–711. 23859934 10.1016/j.ypmed.2013.07.004

[66] Uemura K , Yamada M & Okamoto H (2018) Effects of active learning on health literacy and behavior in older adults: a randomized controlled trial. J Am Geriatr Soc 66, 1721–1729. 30019768 10.1111/jgs.15458

[67] Brewer D , Dickens E , Humphrey A et al. (2016) Increased fruit and vegetable intake among older adults participating in Kentucky’s congregate meal site program. Educ Gerontol 42, 771–784. 28642630 10.1080/03601277.2016.1231511 PMC5476306

[68] Chung LMY & Chung JWY (2014) Effectiveness of a food education program in improving appetite and nutritional status of elderly adults living at home. Asia Pac J Clin Nutr 23, 315–320. 24901103 10.6133/apjcn.2014.23.2.18

[69] Gallois KM , Buck C , Dreas JA et al. (2013) Evaluation of an intervention using a self-regulatory counselling aid: pre- and post- intervention results of the OPTIMAHL 60plus study. Int J Public Health 58, 449–458. 23111370 10.1007/s00038-012-0420-7

[70] Hersey JC , Cates SC , Blitstein JL et al. (2015) Eat smart, live strong intervention increases fruit and vegetable consumption among low-income older adults. J Nutr Gerontol Geriatr 34, 66–80. 25803605 10.1080/21551197.2015.1007199

[71] Hsu H-C , Wang C-H , Chen Y-C et al. (2010) Evaluation of a community-based aging intervention program. Educ Gerontol 36, 547–572.

[72] Lillehoj CJ , Yap L , Montgomery D et al. (2018) Nutritional risk among congregate meal site participants: benefits of a SNAP-Ed program. J Nutr Gerontol Geriatr 37, 204–217. 30285574 10.1080/21551197.2018.1516592

[73] Luten KA , Reijneveld SA , Dijkstra A et al. (2016) Reach and effectiveness of an integrated community-based intervention on physical activity and healthy eating of older adults in a socioeconomically disadvantaged community. Health Educ Res 31, 98–106. 26675175 10.1093/her/cyv064 PMC4883033

[74] MacNab LR , Davis K , Francis SL et al. (2017) Whole grain nutrition education program improves whole grain knowledge and behaviors among community-residing older adults. J Nutr Gerontol Geriatr 36, 189–198. 29252144 10.1080/21551197.2017.1384424

[75] Murayama H , Taguchi A , Spencer MS et al. (2020) Efficacy of a community health worker-based intervention in improving dietary habits among community-dwelling older people: a controlled, crossover trial in Japan. Health Educ Behav 47, 47–56. 31933395 10.1177/1090198119891975

[76] Smith ML , Lee S , Towne SD et al. (2020) Impact of a behavioral intervention on diet, eating patterns, self-efficacy, and social support. J Nutr Educ Behav 52, 180–186. 31540863 10.1016/j.jneb.2019.06.008

[77] Michie S , Richardson M , Johnston M et al. (2013) The behavior change technique taxonomy (v1) of 93 hierarchically clustered techniques: building an international consensus for the reporting of behavior change interventions. Ann Behav Med 46, 81–95. 23512568 10.1007/s12160-013-9486-6

[78] Michie S , West R , Sheals K et al. (2018) Evaluating the effectiveness of behavior change techniques in health-related behavior: a scoping review of methods used. Transl Behav Med 8, 212–224. 29381786 10.1093/tbm/ibx019 PMC6062857

[79] Nigg CR & Long CR (2012) A systematic review of single health behavior change interventions vs. multiple health behavior change interventions among older adults. Transl Behav Med 2, 163–179. 24073109 10.1007/s13142-012-0130-y PMC3717889

[80] Geller K , Lippke S & Nigg CR (2017) Future directions of multiple behavior change research. J Behav Med 40, 194–202. 27785652 10.1007/s10865-016-9809-8

[81] Menezes MC , Diez Roux AV , Costa BVL et al. (2018) Individual and food environmental factors: association with diet. Public Health Nutr 21, 2782–2792. 29976270 10.1017/S1368980018001623 PMC10260899

[82] Vanderlee L , Goorang S , Karbasy K et al. (2019) Policies to create healthier food environments in canada: experts’ evaluation and prioritized actions using the healthy food Environment Policy Index (Food-EPI). Int J Environ Res Public Health 16, 4473. 31739397 10.3390/ijerph16224473 PMC6888279

[83] Blumenthal SJ , Hoffnagle EE , Leung CW et al. (2014) Strategies to improve the dietary quality of Supplemental Nutrition Assistance Program (SNAP) beneficiaries: an assessment of stakeholder opinions. Public Health Nutr 17, 2824–2833. 24476898 10.1017/S1368980013002942 PMC4014633

[84] Higgins MM & Barkley MC (2003) Important nutrition education issues and recommendations related to a review of the literature on older adults. J Nutr Elder 22, 65–78.

[85] Higgins MM & Barkley MC (2003) Concepts, theories and design components for nutrition education programs aimed at older adults. J Nutr Elder 23, 57–75. 14714681 10.1300/J052v23n02_05

[86] Keller HH (2007) Promoting food intake in older adults living in the community: a review. Appl Physiol Nutr Metab 32, 991–1000. 18059571 10.1139/H07-067

[87] Schulz KF , Altman DG , Moher D et al. (2010) CONSORT 2010 statement: updated guidelines for reporting parallel group randomised trials. BMJ 340, c332. 20332509 10.1136/bmj.c332 PMC2844940

[88] Schünemann H , Brożek J , Guyatt G et al. (2013) Handbook for Grading the Quality of Evidence and the Strength of Recommendations using the GRADE Approach. https://gdt.gradepro.org/app/handbook/handbook.html#h.svwngs6pm0f2 (accessed June 2021).

[89] Cherubini A & Gasperini B (2017) How to increase the participation of older subjects in research: good practices and more evidence are needed! Age Ageing 46, 878–881. 10.1093/ageing/afx123 29088365

[90] Higgins MM & Barkley MC (2003) Evaluating outcomes and impact of nutrition education programs designed for older adults. J Nutr Elder 22, 69–81.

[91] Michie S , van Stralen MM & West R (2011) The behaviour change wheel: a new method for characterising and designing behaviour change interventions. Imp Sci 6, 42. 10.1186/1748-5908-6-42 PMC3096582 21513547

[92] French SD , Green SE , O’Connor DA et al. (2012) Developing theory-informed behaviour change interventions to implement evidence into practice: a systematic approach using the theoretical domains framework. Imp Sci 7, 38. 10.1186/1748-5908-7-38 PMC3443064 22531013

[93] Moore AP , Rivas CA , Stanton-Fay S et al. (2019) Designing the Healthy Eating and Active Lifestyles for Diabetes (HEAL-D) self-management and support programme for UK African and Caribbean communities: a culturally tailored, complex intervention under-pinned by behaviour change theory. BMC Public Health 19, 1146–1146. 31429735 10.1186/s12889-019-7411-z PMC6702734

[94] Atkins L & Michie S (2015) Designing interventions to change eating behaviours. Proc Nutr Soc 74, 164–170. 25998679 10.1017/S0029665115000075

[95] Akanni OO , Smith ML & Ory MG (2017) Cost-effectiveness of a community exercise and nutrition program for older adults: texercise select. Int J Environ Res Public Health 14, 1–16. 10.3390/ijerph14050545 PMC5451995 28531094

[96] Ory MG , Lee S , Han G et al. (2018) Effectiveness of a lifestyle intervention on social support, self-efficacy, and physical activity among older adults: evaluation of texercise select. Int J Environ Res Public Health 15, 1–19. 10.3390/ijerph15020234 PMC5858303 29385779

