# Supplementary material for: Group-based nutrition interventions to promote healthy eating and mobility in community-dwelling older adults: a systematic review
Source: Public Health Nutr. 2022 May 16;25(10):2920–51. doi: 10.1017/S136898002200115X (PMC9991860; doi:10.1017/S136898002200115X)
Supplement: Supplementary file 1 [file S136898002200115Xsup.zip › S136898002200115Xsup001.docx]

**Group-based nutrition interventions to promote healthy eating and mobility in community-dwelling older adults: A systematic review**

###### Supplementary Material

###### Supplementary Table 1: MEDLINE (Ovid) Search Strategy

###### Supplementary Table 2: CINAHL (EBSCO) Search Strategy

###### Supplementary Table 3: EMBASE (Ovid) Search Strategy

###### Supplementary Table 4: PsychINFO (Ovid) Search Strategy

###### Supplementary Table 5: Sociological Abstracts (ProQuest) Search Strategy

###### Supplementary Table 6: JBI Critical Appraisal Checklist for Randomized Controlled Trials

Supplementary Table 7: JBI Critical Appraisal Checklist for Quasi-Experimental Studies

Supplementary Appendix 1: Excluded Studies Following Full-Text Review

**Supplementary Table 1**: MEDLINE (Ovid). Search conducted July 15, 2020

| **Search** | **Query** | **Records retrieved** |
| --- | --- | --- |
| #1 | exp Aged OR exp "Aged, 80 and Over" OR exp Elderly OR exp Middle Aged OR Aged OR "Aged, 80 and Over" OR Middle aged OR Elder* OR Older adult* OR Older people | 5,270,176 |
| #2 | Community program* OR Health promotion OR Health education OR Community-based* or Community health services OR Senior centers OR Congregate din* OR Shared din* OR Garden* OR Community intervention OR Community din* | 217,769 |
| #3 | Diet OR Nutrition Therapy OR Nutrition OR Diet, healthy OR Food OR Food assistance OR Healthy eat* OR Food security | 413,356 |
| #4 | #1 AND #2 AND #3 (Limited to 2010-2020, English) | 2,289 |

**Supplementary Table 2**: CINAHL (EBSCO). Search conducted July 19, 2020

| **Search** | **Query** | **Records retrieved** |
| --- | --- | --- |
| #1 | MH Aged+ OR MH Aged, 80 and over+ OR MH Elderly OR MH Middle aged OR Elder* OR Older adult* or Older people* | 860,261 |
| #2 | Community program* OR MH Health promotion OR MH Health education OR Community-based* or MH Community health services OR MH Senior centers OR Congregate din* OR Shared din* OR Garden* OR Community intervention OR Community din* | 90,376 |
| #3 | MH Diet OR Nutrition therapy OR Nutrition OR Healthy diet OR MH Food OR Food assistance OR Healthy eat* OR Food security | 117,323 |
| #4 | #1 AND #2 AND #3 (Limited to 2010-2020, English) | 1,693 |

**Supplementary Table 3**: EMBASE (Ovid). Search conducted on July 15, 2020

| **Search** | **Query** | **Records retrieved** |
| --- | --- | --- |
| #1 | exp Aged OR exp "Aged, 80 and Over" OR exp Elderly OR exp Middle Aged OR Aged OR "Aged, 80 and Over" OR Middle Aged OR Elder* OR Older adult* OR Older people* | 3,488,574 |
| #2 | Community program* OR Health promotion OR Health education OR Community-based* or Community health services OR Senior centers OR Congregate din* OR Shared din* OR Garden* OR Community intervention OR Community din* | 257,448 |
| #3 | Diet OR Nutrition therapy OR Nutrition OR Diet, healthy OR Food OR Food assistance OR Healthy eat* OR Food security | 518,141 |
| #4 | #1 AND #2 AND #3 (Limited to 2010-2020, English) | 2,433 |

**Supplementary Table 4**: PsycINFO (Ovid). Search conducted on July 22, 2020

| **Search** | **Query** | **Records retrieved** |
| --- | --- | --- |
| #1 | exp Aged OR exp "Aged, 80 and Over" OR exp Elderly OR exp Middle Aged OR Aged OR "Aged, 80 and Over" OR Middle Aged OR elder* OR Older adult* OR Older people* | 141,630 |
| #2 | Community program* OR Health promotion OR Health education OR Community-based* or Community senior cent* OR Shared din* OR Congregate din* OR Garden* OR Community intervention OR Community din* | 68,982 |
| #3 | Diet OR Nutrition therapy OR Nutrition OR Diet, healthy OR Food OR Food assistance OR Healthy eat* OR Food security | 71,245 |
| #4 | #1 AND #2 AND #3 (Limited to 2010-2020, English) | 235 |

**Supplementary Table 5**: Sociological Abstracts (ProQuest). Search conducted on July 22, 2020

| **Search** | **Query** | **Records retrieved** |
| --- | --- | --- |
| #1 | Noft (older adult) OR noft (aged) OR noft (old aged) OR noft (older persons) OR noft (65 AND older) OR noft (senior) OR noft (elder*) | 115,337 |
| #2 | Noft (community program*) OR noft (aged health promotion) OR noft (education health) OR noft (community-based) OR noft (seniors care) OR noft (community AND health care) | 116,386 |
| #3 | noft (nutrition) OR noft (diet) OR noft (food) OR noft (dining) OR (eat AND healthy) OR noft (Food Security) AND noft (food AND assistance) | 33,150 |
| #4 | #1 AND #2 AND #3 (Limited to 2010-2020, English) | 344 |

**Supplementary Table 6:** JBI Critical Appraisal Checklist for Randomized Controlled Trials

| **Study** | **Q1** | **Q2** | **Q3** | **Q4** | **Q5** | **Q6** | **Q7** | **Q8** | **Q9** | **Q10** | **Q11** | **Q12** | **Q13** | **Score By Study** |
| --- | --- | --- | --- | --- | --- | --- | --- | --- | --- | --- | --- | --- | --- | --- |
| Francis 2014 | U | U | Y | Y | U | U | U | N | U | Y | Y | Y | Y | **6/13** |
| Geller 2012 | U | U | U | Y | U | U | U | N | Y | Y | Y | N | Y | **5/13** |
| Jancey 2017 | Y | Y | Y | Y | N | N | Y | N | Y | Y | Y | Y | Y | **10/13** |
| Kimura 2013 | U | Y | Y | Y | N | N | U | N | U | Y | Y | Y | Y | **7/13** |
| Lara 2015 | Y | Y | Y | Y | Y | Y | U | Y | Y | Y | U | Y | Y | **11/13** |
| Meethien 2011 | U | U | Y | Y | U | U | U | N | U | Y | Y | Y | Y | **6/13** |
| Mendoza-Ruvalcaba 2015 | U | U | Y | U | U | U | U | U | U | Y | Y | Y | Y | **5/13** |
| Salehi 2011 | U | U | N | Y | U | U | U | U | Y | Y | U | Y | U | **4/13** |
| Silva-Smith 2013 | Y | Y | N | U | U | U | Y | Y | N | Y | Y | Y | Y | **8/13** |
| Uemura 2018 | Y | U | Y | Y | N | N | Y | Y | Y | Y | Y | Y | Y | **10/13** |
| **Total (%)** | **40** | **40** | **70** | **80** | **10** | **10** | **30** | **30** | **50** | **100** | **80** | **90** | **90** |  |
| Note: Y, yes. U, unclear. N, no. 1. Was true randomization used for assignment of participants to treatment groups? 2. Was allocation to treatment groups concealed? 3. Were treatment groups similar at baseline? 4. Were treatment groups treated identically other than the intervention of interest? 5. Were participants blind to treatment assignment? 6. Were those delivering treatment blind to treatment assignment? 7. Were outcomes assessors blind to treatment assignment? 8. Was follow up complete and if not, were differences between groups in terms of their follow up adequately described and analyzed? 9. Were participants analyzed in the groups to which they were randomized? 10. Were outcomes measured in the same way for treatment groups? 11. Were outcomes measured in a reliable way? 12. Was appropriate statistical analysis used? 13. Was the trial design appropriate for the topic, and any deviation from the standard RCT design accounted for in the conduct and analysis? | | | | | | | | | | | | | | |

**Supplementary Table 7:** JBI Critical Appraisal Checklist for Quasi-Experimental Studies

| **Study** | **Q1** | **Q2** | **Q3** | **Q4** | **Q5** | **Q6** | **Q7** | **Q8** | **Q9** | **Score By Study** |
| --- | --- | --- | --- | --- | --- | --- | --- | --- | --- | --- |
| AbuSabha 2011 | Y | U | U | N | N | N | Y | U | Y | **3/9** |
| Beasley 2019 | U | Y | Y | N | Y | Y | Y | Y | Y | **7/9** |
| Brewer 2016 | Y | Y | Y | Y | Y | N | Y | Y | Y | **8/9** |
| Chung 2014 | Y | U | Y | N | Y | Y | Y | Y | Y | **7/9** |
| Gallois 2013 | Y | N | Y | Y | Y | N | Y | Y | Y | **7/9** |
| Hersey 2015 | Y | N | Y | Y | Y | Y | Y | Y | Y | **8/9** |
| Hsu 2010 | Y | N | Y | Y | N | U | Y | U | U | **4/9** |
| Lillehoj 2018 | Y | N | Y | Y | Y | N | Y | Y | N | **6/9** |
| Luten 2016 | Y | N | Y | Y | Y | Y | Y | U | Y | **7/9** |
| MacNab 2017 | Y | Y | Y | N | N | U | Y | N | Y | **5/9** |
| Manafo 2013 | Y | Y | U | N | N | N | Y | U | Y | **4/9** |
| Moreau 2015 | N | Y | Y | N | N | N | Y | U | Y | **4/9** |
| Murayama 2020 | Y | N | Y | Y | Y | Y | Y | U | Y | **7/9** |
| Pogge 2013 | Y | Y | Y | N | N | N | Y | U | Y | **5/9** |
| Schwingel 2017 | Y | Y | Y | N | Y | U | Y | Y | Y | **7/9** |
| Smith 2015 | Y | Y | Y | N | Y | N | Y | U | Y | **6/9** |
| Smith 2020 | Y | N | Y | Y | Y | N | Y | U | Y | **6/9** |
| Strout 2017 | Y | Y | Y | N | N | Y | Y | U | U | **5/9** |
| Thomas 2010 | Y | N | U | N | N | N | Y | N | N | **2/9** |
| Turk 2016 | Y | Y | Y | N | N | Y | Y | U | Y | **6/9** |
| Wunderlich 2011 | Y | Y | U | N | N | N | Y | U | N | **3/9** |
| **Total (%)** | **90.5** | **52.4** | **81.0** | **38.1** | **52.4** | **33.3** | **100.0** | **33.3** | **76.2** |  |
| Note: Y, yes. U, unclear. N, no. 1. Is it clear in the study what is the “cause” and what is the “effect” (i.e., there is no confusion about which variable comes first)? 2. Were the participants included in any comparisons similar? 3. Were the participants included in any comparisons receiving similar treatment/care, other than the exposure or intervention of interest? 4. Was there a control group? 5. Were there multiple measurements of the outcome both pre and post the intervention/exposure? 6. Was follow up complete and if not, were differences between groups in terms of their follow up adequately described and analyzed? 7. Were the outcomes of participants included in any comparisons measured in the same way? 8. Were outcomes measured in a reliable way? 9. Was appropriate statistical analysis used? | | | | | | | | | | |

**Supplementary Appendix 1: Excluded Studies Following Full-Text Review**

1. Aburrow A., Wallis K., Steward K., Cholet A., Murphy J.L. Managing malnutrition (as undernutrition) and caring for older people living in the community: The development and publishing of a new workbook and training videos for staff working in community teams (e.g. nursing, integrated and therapy teams). Clinical Nutrition ESPEN 2020;35:247-248.

*Reason for exclusion*: Wrong patient population

1. Ahn J., Park J., Kim C. Effects of an individualised nutritional education and support programme on dietary habits, nutritional knowledge and nutritional status of older adults living alone. Journal of Clinical Nursing 2018;27(9-10):2142-2151.

*Reason for exclusion*: Wrong intervention

1. Ajala O., Erqou S., Bambs C., Sharbaugh M., Althouse A., Aiyer A., Kip K., Reis S. Ideal cardiovascular health metrics in couples: A community-based population study. Journal of the American College of Cardiology 2017;69(11):1810.

*Reason for exclusion*: Wrong study design

1. Alemu, F., Mecha, M., Medhin, G. Impact of permagarden intervention on improving fruit and vegetable intake among vulnerable groups in an urban setting of Ethiopia: A quasi-experimental study. PloS one 2019;14(12):e0213705.

*Reason for exclusion*: Wrong patient population

1. Ali N.M., Shahar S., Kee Y.L. Norizan A.R., Noah S.A.M. Design of an interactive digital nutritional education package for elderly people. Informatics for Health & Social Care 2012;37(4):217-29.

*Reason for exclusion*: Wrong setting

1. An R. Association of home-delivered meals on daily energy and nutrient intakes: Findings from the National Health and Nutrition Examination Surveys. Journal of Nutrition in Gerontology and Geriatrics 2015;34(2):263-272.

*Reason for exclusion*: Wrong study design

1. Anderson A.L, Netterville L.L., Sahyoun N.R. "Food safety on the go": A course for home-delivered meal programs. Journal of Nutrition Education and Behavior 2014;46(2):148-150.

*Reason for exclusion*: Wrong setting

1. Apovian, C.M., Singer M.R., Campbell W.W., Bhasin S., McCarthy A.C., Shah M., Basaria S., Moore L.L. Development of a Novel Six-Month Nutrition Intervention for a Randomized Trial in Older Men with Mobility Limitations. The Journal of Nutrition, Health & Aging 2017;21(10):1081-1088.

*Reason for exclusion*: Wrong intervention

1. Arcury T.A., Nguyen H.T., Sandberg J.C., Neiberg R.H., Altizer K.P., Bell, Ronny A, Grzywacz J.G., Lang W., Quandt, S.A. Use of Complementary Therapies for Health Promotion Among Older Adults. Journal of Applied Gerontology 2015;34(5):552.

*Reason for exclusion*: Wrong intervention

1. Astley S. Exploitation of European Union Framework Programme results in food, health and wellbeing by small‐ and medium‐sized enterprises: The ‘Quantify Life – Feed Yourself’ (QuaLiFY) project. Nutrition Bulletin 2018;43(2):195-201.

*Reason for exclusion*: Wrong study design

1. Aure C.F., Kluge A., Moen A. Promoting dietary awareness: Home-dwelling older adults' perspectives on using a nutrition application. International Journal of Older People Nursing 2020;15(4):e12332.

*Reason for exclusion:* Wrong setting

1. Austin B., Royster J. Exploring the Use of Skype Technology for Teaching Food, Nutrition and Health to African American Elders in Seattle, Washington: A Pilot Program. Journal of the Academy of Nutrition & Dietetics Supplement 2016;116(9):A80.

*Reason for exclusion*: Wrong study design

1. Ayala G.X., Baquero B., Pickrel J.L., Mayer J., Belch G., Rock C.L., Linnan L., Gittelsohn J., Sanchez-Flack J., Elder J.P. A store-based intervention to increase fruit and vegetable consumption: The El Valor de Nuestra Salud cluster randomized controlled trial. Contemporary Clinical Trials 2015;42:228-38.

*Reason for exclusion*: Wrong patient population

1. Bahl M., Francis S.L., Yap L., Montgomery D., Lillehoj C. Fresh Conversations, a SNAP-ed program for older adults: Feedback from program facilitators. Journal of Nutrition Education and Behavior 2019;51(4):486-491.

*Reason for exclusion*: Wrong study design

1. Ball K., McNaughton S.A., Mhurchu C.N., Andrianopoulos N., Inglis V., McNeilly B., Le H.N.D., Leslie D., Pollard C., Crawford D. Supermarket Healthy Eating for Life (SHELf): protocol of a randomised controlled trial promoting healthy food and beverage consumption through price reduction and skill-building strategies. BMC Public Health 2011;11(1):1-11.

*Reason for exclusion*: Wrong patient population

1. Bandayrel K., Wong S. Systematic literature review of randomized control trials assessing the effectiveness of nutrition interventions in community-dwelling older adults. Journal of Nutrition Education and Behavior 2011;43(4):251-62.

*Reason for exclusion*: Wrong study design

1. Barnfield A., Savolainen N., Lounamaa A. Health promotion interventions: Lessons from the transfer of good practices in CHRODIS-PLUS. International Journal of Environmental Research and Public Health 2020;17(4):1281.

*Reason for exclusion*: Wrong study design

1. Basu S., Gardner C.D., White J.S., Rigdon J., Carroll M.M., Akers M., Seligman H.K. Effects Of Alternative Food Voucher Delivery Strategies On Nutrition Among Low-Income Adults. Health Affairs 2019;38(4):577-584.

*Reason for exclusion*: Wrong patient population

1. Bech-Larsen T., Kazbare L. Spillover of diet changes on intentions to approach healthy food and avoid unhealthy food. Health Education 2014;114(5):367-377.

*Reason for exclusion*: Wrong study design

1. Begley A., Paynter E., Butcher L.M., Dhaliwal S.S. Effectiveness of an Adult Food Literacy Program. Nutrients 2019;11(4):797.

*Reason for exclusion*: Wrong patient population

1. Bertram A.G., Burr B.K., Sears K., Powers M., Atkins L., Holmes T., Kambour T., Kuns J.B. Generations learning together: pilot study for a multigenerational program. Journal of Intergenerational Relationships 2018;16(3):243-255.

*Reason for exclusion*: Wrong intervention

1. Besdine R.W., Wetle T.F. Improving health for elderly people: an international health promotion and disease prevention agenda. Aging Clinical and Experimental Research 2010;22(3):219-30.

*Reason for exclusion*: Wrong intervention

1. Bhurosy T., Jeewon R. Effectiveness of a theory-driven nutritional education program in improving calcium intake among older mauritian adults. Scientific World Journal 2013;750128:1-16.

*Reason for exclusion*: Wrong patient population

1. Bhurosy T., Jeewon R. Effectiveness of a theory-driven nutritional education program in improving calcium intake among older mauritian adults. Scientific World Journal 2014;750128:1-16.

*Reason for exclusion*: Wrong patient population

1. Bihan H., Mejean C., Castetbon K., Faure H., Ducros V., Sedeaud A., Galan P., Le Clesiau H., Peneau S., Hercberg S. Impact of fruit and vegetable vouchers and dietary advice on fruit and vegetable intake in a low-income population. European Journal of Clinical Nutrition 2012;66(3):369-75.

*Reason for exclusion*: Wrong patient population

1. Block G., Azar K.M.J., Romanelli R.J., Block T.J., Palaniappan L.P., Dolginsky M., Block C.H. Improving diet, activity and wellness in adults at risk of diabetes: randomized controlled trial. Nutrition & Diabetes 2016;6(9):e231.

*Reason for exclusion*: Wrong intervention

1. Bobitt J.L. Factors impacting the implementation of evidence-based wellness programs in Illinois senior centers. Dissertation Abstracts International: Section B: The Sciences and Engineering 2015.

*Reason for exclusion*: Wrong study design

1. Bracher M., Steward K., Wallis K., May C.R., Aburrow A., Murphy J. Implementing professional behaviour change in teams under pressure: Results from phase one of a prospective process evaluation (the Implementing Nutrition Screening in Community Care for Older People (INSCCOPe) project). BMJ Open 2019;9(8):e025966.

*Reason for exclusion*: Wrong intervention

1. Brand T., Pischke C.R., Steenbock B., Schoenbach J., Poettgen S., Samkange-Zeeb F., Zeeb, H. What works in community-based interventions promoting physical activity and healthy eating? A review of reviews. International Journal of Environmental Research and Public Health 2014;11(6):5866-88.

*Reason for exclusion*: Wrong study design

1. Broekhuizen K., Kroeze W., van Poppel M.N.M., Oenema A., Brug J. A systematic review of randomized controlled trials on the effectiveness of computer-tailored physical activity and dietary behavior promotion programs: An update. Annals of Behavioral Medicine 2012;44(2):259-286.

*Reason for exclusion*: Wrong patient population

1. Buman M.P., Winter S.J., Baker C., Hekler E.B., Otten J.J., King A.C. Neighborhood Eating and Activity Advocacy Teams (NEAAT): Engaging older adults in policy activities to improve food and physical environments. Translational Behavioral Medicine 2012;2(2):249-253.

*Reason for exclusion*: Wrong study design

1. Burke D., Jennings M., McClinchy J., Masey H., Westwood D., Dickinson, A. Community luncheon clubs benefit the nutritional and social well-being of free living older people. Journal of Human Nutrition & Dietetics 2011;24(3):278.

*Reason for exclusion*: Wrong study design

1. Burke L., Jancey J., Howat P., Lee A., Kerr D., Shilton T., Hills A., Anderson A. Physical activity and nutrition program for seniors (PANS): protocol of a randomized controlled trial. BMC Public Health 2010;10(1):1-7.

*Reason for exclusion*: Wrong intervention

1. Buys D.R., Locher Julie L What Does the Evidence Reveal Regarding Home- and Community-Based Nutrition Services for Older Adults? Journal of Nutrition in Gerontology and Geriatrics 2015;34(2):81-4.

*Reason for exclusion*: Wrong study design

1. Buys, David R; Marler, Malcolm L; Robinson, Caroline O; Hamlin, Christopher M; Locher, Julie L. Recruitment of volunteers for a home-delivered meals programme serving homebound older adults: a theoretically derived programme among faith communities. Public Health Nutrition 2011;14(8):1473-8.

*Reason for exclusion*: Wrong study design

1. Buyuktuncer Z., Kearney M., Ryan C.L., Thurston M., Ellahi B. Fruit and vegetables on prescription: a brief intervention in primary care. Journal of Human Nutrition and Dietetics: The Official Journal of the British Dietetic Association 2014;27(2):186-193.

*Reason for exclusion*: Wrong patient population

1. Campbell A.D., Godfryd A., Buys D.R., Locher J.L. Does participation in home-delivered meals programs improve outcomes for older adults? Results of a systematic review. Journal of Nutrition in Gerontology and Geriatrics 2015;34(2):124-167.

*Reason for exclusion*: Wrong study design

1. Carney P.A., Hamada J.L., Rdesinski R., Sprager L., Nichols K.R., Liu B.Y., Pelayo J., Sanchez M.A., Shannon J. Impact of a community gardening project on vegetable intake, food security and family relationships: a community-based participatory research study. Journal of Community Health 2012;37(4):874-81.

*Reason for exclusion*: Wrong patient population

1. Carter S.R., Walker A., Abdul-Latif S., Maurer L., Masunungure D., Tedaldi E., Patterson F. Nice to your heart: A pilot community-based intervention to improve heart health behaviours in urban residents. Health Education Journal 2015;75(3):306-317.

*Reason for exclusion*: Wrong patient population

1. Cavill J., Jancey J., Howat P. Review and recommendations for online physical activity and nutrition programmes targeted at over 40s. Global Health Promotion 2012;19(2):44-53.

*Reason for exclusion*: Wrong patient population

1. Charlton K.E., Walton K., Moon L., Smith K., McMahon A.T., Ralph F., Stuckey M., Manning F., Krassie J. "It could probably help someone else but not me": A feasibility study of a snack programme offered to meals on wheels clients. Journal of Nutrition, Health and Aging 2013;17(4):364-369.

*Reason for exclusion*: Wrong setting

1. Chastonay P., Weber D., Mattig T. A Nationally Coordinated Health Promotion Program for the Elderly in a Federal State, Switzerland. Journal of Population Ageing 2019;13(3):1-14.

*Reason for exclusion*: Wrong study design

1. Chedraui P., Pérez-López F.R. Nutrition and health during mid-life: searching for solutions and meeting challenges for the aging population. Climacteric Supplement 2013;16(1):85-95.

*Reason for exclusion*: Wrong study design

1. Chen Y., Wu F., Wu Y., Li J., Yue P., Deng Y., Lamb K.V., Fong S., Liu Y., Zhang Y. Development of interventions for an intelligent and individualized mobile health care system to promote healthy diet and physical activity: using an intervention mapping framework. BMC Public Health 2019;19(1):1311.

*Reason for exclusion*: Wrong study design

1. Chiu C., Kuo S., Lin D. Technology-embedded health education on nutrition for middle-aged and older adults living in the community. Global Health Promotion 2019;26(3):80-87.

*Reason for exclusion*: Wrong setting

1. Chojenta C., Mingay E., Gresham E., Byles J. Cooking for One or Two: Applying Participatory Action Research to improve community-dwelling older adults' health and well-being. Health Promotion Journal of Australia 2018;29(1):105-107

*Reason for exclusion*: Wrong intervention

1. Churchill S., Pavey L. Promoting fruit and vegetable consumption: the role of message framing and autonomy. British Journal of Health Psychology 2013;18(3):610-22.

*Reason for exclusion*: Wrong patient population

1. Clark P.G., Blissmer B.J., Greene G.W., Lees F.D., Riebe D.A., Stamm K.E. Maintaining exercise and healthful eating in older adults: the SENIOR project II: study design and methodology. Contemporary Clinical Trials 2011;32(1):129-39.

*Reason for exclusion*: Wrong patient population

1. Clark P.G., Greene G.W., Blissmer B.J., Lees F.D., Riebe D.A., Stamm K.E. Trajectories of Maintenance and Resilience in Healthful Eating and Exercise Behaviors in Older Adults. Journal of Aging and Health 2019;31(5):861-882.

*Reason for exclusion*: Wrong intervention

1. Coberley C., Rula E.Y., Pope J.E. Effectiveness of health and wellness initiatives for seniors. Population Health Management 2011;14(1):S45-50.

*Reason for exclusion*: Wrong intervention

1. Cohen A.J., Lachance L.L., Richardson C.R., Mahmoudi E., Buxbaum J.D., Noonan G.K., Murphy E.C., Roberson D.N., Hesterman O.B., Heisler M., Zick S.M. "Doubling Up" on Produce at Detroit Farmers Markets: Patterns and Correlates of Use of a Healthy Food Incentive. American Journal of Preventive Medicine 2018;54(2):181-189.

*Reason for exclusion*: Wrong patient population

1. Conneely M., Andrews S., Axelrod C., Bevier W., Chavez E., Swartzentruber J., Kerr D. Ocho Pasos A La Buena Salud (eight steps to better health): A successful community group education program for Latino adults with or at risk for type 2 diabetes. Diabetes 2017;66(1):A173.

*Reason for exclusion*: Wrong patient population

1. Connell C.L., Thomson J.L., Huye H.F., Landry A.S., Crook L.B., Yadrick K. Mississippi Communities for Healthy Living: Implementing a nutrition intervention effectiveness study in a rural health disparate region. Contemporary Clinical Trials 2015;42:196-203.

*Reason for exclusion*: Wrong patient population

1. Contrady, A.; Francis, S.; Montgomery, D. Fresh Conversations: Helping Older Adults Make Lifestyle Changes...Food & Nutrition Conference & Expo, 26-29 October 2019, Philadelphia, PA. Journal of the Academy of Nutrition & Dietetics Supplement 2019;119(9):A69-A69.

*Reason for exclusion*: Conference abstract only

1. Cook R.F., Hersch R.K., Schlossberg D., Leaf S.L. A Web-based health promotion program for older workers: randomized controlled trial. Journal of Medical Internet Research 2015;17(3):e82.

*Reason for exclusion*: Wrong intervention

1. Corcoran M.P., Nelson M.E., Sacheck J.M., Reid K.F., Kirn D., Fielding R.A., Chui K.K.H., Folta S.C. Efficacy of an Exercise and Nutritional Supplement Program on Physical Performance and Nutritional Status in Older Adults With Mobility Limitations Residing at Senior Living Facilities. Journal of Aging & Physical Activity 2017;25(3):453-463.

*Reason for exclusion*: Wrong intervention

1. Corcoran M.P., Nelson M.E., Sacheck J.M., Reid K.F., Kirn D., Fielding R.A., Folta S.C. Recruitment of Mobility Limited Older Adults Into a Facility-Led Exercise-Nutrition Study: The Effect of Social Involvement. The Gerontologist 2016;56(4):669.

*Reason for exclusion*: Wrong study design

1. Costa A.I.A., Jongen W.M.F. Designing new meals for an ageing population. Critical Reviews in Food Science and Nutrition 2010;50(6):489-502.

*Reason for exclusion*: Wrong study design

1. Cotter E.W., Teixeira C., Bontrager A., Horton K., Soriano D. Low-income adults' perceptions of farmers' markets and community-supported agriculture programmes. Public Health Nutrition 2017;20(8):1452-1460.

*Reason for exclusion*: Wrong study design

1. Curbach J., Warrelmann B., Brandstetter S., Lindacher V., Rueter J., Loss J. Empowering senior citizens for healthy nutrition in Germany: A pilot study. Health & Social Care in the Community 2018;26(5):675-684.

*Reason for exclusion*: Wrong study design

1. Cuthbert E.M.E. Garden-based nutrition education to increase older African Americans' consumption of fresh fruits and vegetables. Dissertation Abstracts International Section A: Humanities and Social Sciences 2016.

*Reason for exclusion*: Conference abstract only

1. Dapp U., Neumann L., Golgert S., Klugmann B., Profener F., Von Renteln-Kruse W., Minder C. Long-term effects of the intervention "active health promotion in old age" over a 13 year period-results from the LUCAS cohort study (prolong health). European Journal of Epidemiology 2016;31(1):S39.

*Reason for exclusion*: Wrong intervention

1. de Souto Barreto P., Rolland Y., Maltais M., Vellas B. Associations of Multidomain Lifestyle Intervention with Frailty: Secondary Analysis of a Randomized Controlled Trial. American Journal of Medicine 2018;131(11):1382.

*Reason for exclusion*: Wrong intervention

1. Dexter A.S., Pope J.F., Erickson D., Fontenot C., Ollendike E., Walker E. Cooking Education Improves Cooking Confidence and Dietary Habits in Veterans. The Diabetes Educator 2019;45(4):442-449.

*Reason for exclusion*: Wrong patient population

1. DiMaria-Ghalili R.A., Laverty N., Baron K., Nasser J.A. Benchmarking a Home-Delivered Meal Program's Annual Satisfaction Survey: A Metropolitan Area Neighborhood Nutrition Alliance (MANNA) Initiative in Philadelphia. Journal of Nutrition in Gerontology and Geriatrics 2015;34(2):189-206.

*Reason for exclusion*: Wrong study design

1. Dirige O.V., Carlson J.A., Alcaraz J., Moy K.L., Rock C.L., Oades R., Sallis J.F. Siglang Buhay: nutrition and physical activity promotion in Filipino-Americans through community organizations. Journal of Public Health Management and Practice 2013;19(2):162-8.

*Reason for exclusion:* Wrong patient population

1. Dorner T.E., Lackinger C., Haider S., Luger E., Kapan A. Luger M., Schindler K.E. Nutritional intervention and physical training in malnourished frail community-dwelling elderly persons carried out by trained lay “buddies”: study protocol of a randomized controlled trial. BMC Public Health 2013;13(1):1232.

*Reason for exclusion*: Wrong setting

1. Dover S.E., Buys D.R., Allocca S., Locher J.L. Farmers' Market Produce Delivery Program for Mitigating Nutritional Risk in Older Adults. Journal of Hunger and Environmental Nutrition 2013;8(1):106-108.

*Reason for exclusion*: Wrong study design

1. Downes L.S., Buchholz S.W., Bruster B., Girimurugan S.B., Fogg L.F., Frock M.S. Delivery of a community-based nutrition education program for minority adults. Journal of the American Association of Nurse Practitioners 2019;31(4):269-277.

*Reason for exclusion*: Wrong patient population

1. Duncan A.M., Dunn H.A., Stratton L.M., Vella M.N. Translating knowledge into dietetic practice: a Functional Foods for Healthy Aging Toolkit. Applied Physiology, Nutrition, and Metabolism 2014;39(5):600-3.

*Reason for exclusion*: Wrong study design

1. Ellis J.L., Morzinski J.A. Reaching and Supporting At-Risk Community Based Seniors: Results of a Multi-church Partnership. Journal of Community Health 2018;43(5):920-928.

*Reason for exclusion*: Wrong intervention

1. Endevelt R., Lemberger J., Bregman J., Kowen G., Berger-Fecht I., Lander H., Karpati T., Shahar D.R. Intensive dietary intervention by a dietitian as a case manager among community dwelling older adults: the EDIT study. The Journal of Nutrition, Health & Aging 2011;15(8):624-30.

*Reason for exclusion*: Wrong patient population

1. Fallaize R., Macready A.L., Butler L.T., Ellis J.A., Berezowska A., Fischer A.R.H., Walsh M.C., Gallagher C., Stewart-Knox B.J., Kuznesof S., Frewer L.J, Gibney M.J., Lovegrove J.A. The perceived impact of the National Health Service on personalised nutrition service delivery among the UK public. The British Journal of Nutrition 2015;113(8):1271-9.

*Reason for exclusion*: Wrong patient population

1. Farsjo C., Kluge A., Moen A. Using a tablet application about nutrition in home care-Experiences and perspectives of healthcare professionals. Health & Social Care in the Community 2019;27(3):683-692.

*Reason for exclusion*: Wrong patient population

1. Feldman C., Mahadevan M., Su H., Brusca J., Ruzsilla J. Menu engineering: A strategy for seniors to select healthier meals. Perspectives in Public Health 2011;131(6):267-274.

*Reason for exclusion*: Wrong study design

1. Fernandez-Barres S., Garcia-Barco M., Basora Josep., Martinez T., Pedret R., Arija V. The efficacy of a nutrition education intervention to prevent risk of malnutrition for dependent elderly patients receiving Home Care: A randomized controlled trial. International Journal of Nursing Studies 2017;70:131-141.

*Reason for exclusion*: Wrong intervention

1. Franzen-Castle L., Versch R. Transitioning Nutrition Education Programming Delivery to Online Formats. Journal of Nutrition Education & Behavior 2014;46(5):454-455.

*Reason for exclusion*: Wrong intervention

1. Friedman S., Rankin P., Morton D. Do older adults benefit from lifestyle modification in the Coronary Health Improvement Program (CHIP)? Journal of the American Geriatrics Society 2017;65(1):S144.

*Reason for exclusion*: Wrong intervention

1. Friesen C.A. Seniors Eating Well: A Sourcebook of Lesson Plans and Activities for Educating Older Adults About Nutrition and Health. Journal of Nutrition Education & Behavior 2017;49(9):796.

*Reason for exclusion*: Wrong study design

1. Frost R., Belk C., Jovicic A., Ricciardi F., Kharicha K., Gardner B., Iliffe S., Goodman C., Manthorpe J., Drennan V.M., Walters K. Health promotion interventions for community-dwelling older people with mild or pre-frailty: a systematic review and meta-analysis. BMC Geriatrics 2017;17(1):157.

*Reason for exclusion*: Wrong study design

1. Fuglerud K.S., Leister W., Bai A., Farsjø C., Moen A. Inspiring Older People to Eat Healthily...15th International Conference on Wearable, Micro and Nano Technologies for Personalized Health, 12-14 June 2018, Gjøvik, Norway. Studies in Health Technology & Informatics 2018;249:194-198.

*Reason for exclusion*: Wrong study design

1. Gans K.M., Risica P.M., Dulin-Keita Ak., Mello J., Dawood M., Strolla L.O.; Harel O. Innovative video tailoring for dietary change: final results of the Good for you! cluster randomized trial. The International Journal of Behavioral Nutrition and Physical Activity 2015;12(1):1-13.

*Reason for exclusion*: Wrong intervention

1. Garcia A.L., Reardon R., Hammond E., Parrett A., Gebbie-Diben A. Evaluation of the "Eat Better Feel Better" Cooking Programme to Tackle Barriers to Healthy Eating. International Journal of Environmental Research and Public Health 2017;14(4):380.

*Reason for exclusion*: Wrong patient population

1. Geaney F., Kelly C., Di Marrazzo J.S., Harrington J.M., Fitzgerald A.P., Greiner B.A., Perry I.J. The effect of complex workplace dietary interventions on employees' dietary intakes, nutrition knowledge and health status: A cluster controlled trial. Preventive Medicine 2016;89:76-83.

*Reason for exclusion*: Wrong patient population

1. Gené H.L., Navarro G.M., Kostov B., Ortega C.M., Colungo F.C., Carpallo N.M., Hervás D.A., Vilarrasa S.R., García P.R.; Sisó-Almirall A. Pre Frail 80: Multifactorial Intervention to Prevent Progression of Pre-Frailty to Frailty in the Elderly. Journal of Nutrition, Health & Aging 2018;22(10):1266-1274.

*Reason for exclusion*: Wrong intervention

1. Gergerich E., Shobe M., Christy K. Sustaining our nation's seniors through federal food and nutrition programs. Journal of Nutrition in Gerontology and Geriatrics 2015;34(3):273-291.

*Reason for exclusion*: Wrong study design

1. Getty M., Amella E., Mueller M., Finney M., Fraser, A. Self-Reported Knowledge by Older Adults after Exposure to Cooking Healthy, Eating Smart: A Community-Based Nutrition Intervention...2014 Food & Nutrition Conference & Expo, October 18-21, 2014, Atlanta, GA. Journal of the Academy of Nutrition & Dietetics 2014;114(9):A55.

*Reason for exclusion*: Conference abstract only

1. Gilbert R.M., Rawlings A., Dixon M., Irving S.S.C., Sivaprasad S. Dietary change for macular health of older people: Eating for Eye Health, a pilot community kitchens intervention. The Lancet 2018;392(2):S37.

*Reason for exclusion*: Wrong intervention

1. Gilbride J.A. The Multistate Research Experience Reexamined: Focus on Food and Nutrition Approaches for Older Adults. Topics in Clinical Nutrition 2020;35(1):3-8.

*Reason for exclusion*: Wrong study design

1. Glasson C., Chapman K., Wilson T. Gander K., Hughes C., Hudson N., James E. Increased exposure to community-based education and 'below the line' social marketing results in increased fruit and vegetable consumption. Public Health Nutrition 2013;16(11):1961-70.

*Reason for exclusion*: Wrong patient population

1. Gomez-Busto F., Andia V., Prada S., Nadador N., Garagalza A., Elkoroaristizabal X., Ibarra B., Lopez de la Puente A. Nutritional status of elderly users of a home-delivered meals service. European Geriatric Medicine 2010;1(1):S133.

*Reason for exclusion*: Conference abstract only

1. Gotwals B. Self-Efficacy and Nutrition Education: A Study of the Effect of an Intervention with Faith Community Nurses. Journal of Religion & Health 2018;57(1):333-348.

*Reason for exclusion*: Wrong patient population

1. Guenther P.M., Luick B.R. Improved Overall Quality of Diets Reported by Expanded Food and Nutrition Education Program Participants in the Mountain Region. Journal of Nutrition Education and Behavior 2015;47(5):421-6.

*Reason for exclusion*: Wrong patient population

1. Guillaumie L., Godin G., Manderscheid J., Spitz E., Muller L. The impact of self-efficacy and implementation intentions-based interventions on fruit and vegetable intake among adults. Psychology & Health 2012;27(1):30-50.

*Reason for exclusion*: Wrong patient population

1. Guillaumie L., Godin G., Manderscheid J., Spitz E., Muller L. Self-efficacy and implementation intentions-based interventions on fruit and vegetable intake among adults: impact at 12-month follow-up. Global Health Promotion 2013;20(2):83-7.

*Reason for exclusion*: Wrong patient population

1. Gutschall M.D., Marchetti J.M., Thompson K.L. Counseling Strategies to Improve Nutrition Care for Rural Appalachian Patients. Topics in Clinical Nutrition 2019;34(1):77-87.

*Reason for exclusion*: Wrong study design

1. Hale D., Marshall K. Healthy Aging: How It’s Done. Home Healthcare Now 2018;36(5):326-326.

*Reason for exclusion*: Wrong intervention

1. Hamirudin A.H., Carrie A., Charlton K., Walton K., Tapsell L., Milosavljevic M., Pang G., Potter J. Home-based dietetic intervention improves nutritional status post hospital discharge in older people. Annals of Nutrition and Metabolism 2013;63(1):652.

*Reason for exclusion*: Wrong patient population

1. Harland, J. I.; Buttriss, J.; Gibson, S. Achieving eatwell plate recommendations: is this a route to improving both sustainability and healthy eating? Nutrition Bulletin 2012;37(4):324-343.

*Reason for exclusion*: Wrong patient population

1. Hassel H., Schulte B., Keimer K.M. Participatory development of an instrument for the elderly for an autonomous optimization of their nutrition and physical activity profile. Health Education Journal 2010;69(3):353-361.

*Reason for exclusion*: Wrong study design

1. Hau C., Reid K.F., Wong K.F., Chin R.J., Botto T.J., Eliasziw M., Bermudez O.I., Fielding R.A. Collaborative Evaluation of the Healthy Habits Program: An Effective Community Intervention to Improve Mobility and Cognition of Chinese Older Adults Living in the U.S. The Journal of Nutrition, Health & Aging 2016;20(4):391-7.

*Reason for exclusion*: Wrong intervention

1. Hazavehei S.M.M., Afshari M. The role of nutritional interventions in increasing fruit and vegetable intake in the elderlies: a systematic review. Aging Clinical and Experimental Research 2016;28(4):583-598.

*Reason for exclusion*: Wrong study design

1. Hetherington, S.A., Borodzicz J.A., Shing C.M. Assessing the real world effectiveness of the Healthy Eating Activity and Lifestyle (HEAL) program. Health Promotion Journal of Australia: Official Journal of Australian Association of Health Promotion Professionals 2015;26(2):93-98.

*Reason for exclusion*: Wrong patient population

1. Hekmatpou D., Shamsi M., Zamani M. The effect of a healthy lifestyle program on the elderly's health in Arak. Indian Journal of Medical Sciences 2013;67(3-4):70-7.

*Reason for exclusion*: Wrong intervention

1. Herbert J., Flego A., Gibbs L., Waters E., Swinburn B., Reynolds J., Moodie M. Wider impacts of a 10-week community cooking skills program--Jamie's Ministry of Food, Australia. BMC Public Health 2014;14(1):1-4.

*Reason for exclusion*: Wrong patient population

1. Herghelegiu A.M.; Wenzel K.M.; Moser A.; Prada G.I.; Nuta C.R.; Stuck A.E. Effects of Health Risk Assessment and Counselling on Fruit and Vegetable Intake in Older People: A Pragmatic Randomised Controlled Trial. Journal of Nutrition, Health and Aging 2020;24(6):591-597.

*Reason for exclusion*: Wrong intervention

1. Hillier F.C., Batterham A.M., Nixon C.A., Crayton A.M., Pedley C.L., Summerbell C.D. A community-based health promotion intervention using brief negotiation techniques and a pledge on dietary intake, physical activity levels and weight outcomes: lessons learnt from an exploratory trial. Public Health Nutrition 2012;15(8):1446-55.

*Reason for exclusion*: Wrong patient population

1. Hoerr K.A., Francis S.L., Margrett J.A., Peterson M., Franke W.D. Promoting the Congregate Meal Program to the next generation of rural-residing older adults. Journal of Nutrition in Gerontology and Geriatrics 2016;35(2):113-123.

*Reason for exclusion*: Wrong study design

1. Horta P.M., dos Santos L.C. Effectiveness of high-intensity nutritional interventions for overweight Brazilian women. Public Health 2016;139:231-233.

*Reason for exclusion*: Wrong patient population

1. Horwath C.C., Schembre S.M., Motl R.W., Dishman R.K., Nigg C.R. Does the transtheoretical model of behavior change provide a useful basis for interventions to promote fruit and vegetable consumption?. American Journal of Health Promotion 2013;27(6):351-7.

*Reason for exclusion*: Wrong patient population

1. Host A., McMahon A., Walton K., Charlton K. Factors Influencing Food Choice for Independently Living Older People-A Systematic Literature Review. Journal of Nutrition in Gerontology and Geriatrics 2016;35(2):67-94.

*Reason for exclusion*: Wrong study design

1. Hsiao B., Sibeko L., Troy L.M. A Systematic Review of Mobile Produce Markets: Facilitators and Barriers to Use, and Associations with Reported Fruit and Vegetable Intake. Journal of the Academy of Nutrition and Dietetics 2019;119(1):76-97.

*Reason for exclusion*: Wrong study design

1. Hsu H., Kuo T., Lin J., Hsu W., Yu C., Chen Y., Xie W., Hsu W., Hsu Y., Yu M. A Cross-Disciplinary Successful Aging Intervention and Evaluation: Comparison of Person-to-Person and Digital-Assisted Approaches. International Journal of Environmental Research and Public Health 2018;15(5):913.

*Reason for exclusion*: Wrong intervention

1. Hughes S.L., Seymour R.B., Campbell R.T., Shaw J.W., Fabiyi C., Sokas R. Comparison of two health-promotion programs for older workers. American Journal of Public Health 2011;101(5):883-90.

*Reason for exclusion*: Wrong patient population

1. Hutchinson J., Watt J.F., Strachan E.K., Cade J.E. Evaluation of the effectiveness of the Ministry of Food cooking programme on self-reported food consumption and confidence with cooking. Public Health Nutrition 2016;19(18):3417-3427.

*Reason for exclusion*: Wrong patient population

1. Ibrahim N., Moy F.M., Awalludin I.A.N., Ali Z.M., Ismail I.S. Effects of a community-based healthy lifestyle intervention program (Co-HELP) among adults with prediabetes in a developing country: A quasi-experimental study. PLoS ONE 2016;11(12):e0167123.

*Reason for exclusion*: Wrong patient population

1. Ivery J.M., Benton L., Harrison A., Paul M., Cortes M. The DASH Pilot Project: Developing Community-Based Nutrition Education for Older Adults. Journal of Gerontological Social Work 2017;60(4):286-299.

*Reason for exclusion*: Wrong study design

1. Jadczak A.D., Luscombe-Marsh N., Taylor P., Barnard R., Makwana N., Visvanathan R. The EXPRESS Study: Exercise and Protein Effectiveness Supplementation Study supporting autonomy in community dwelling frail older people-study protocol for a randomized controlled pilot and feasibility study. Pilot and Feasibility Studies 2018;4(1):1-10.

*Reason for exclusion*: Wrong patient population

1. Jih J. Le G., Woo K., Tsoh J.Y. Stewart S., Gildengorin G., Burke A., Wong C., Chan E., Fung L., Yu F., Pasick R., McPhee S.J., Nguyen T.T. Educational Interventions to Promote Healthy Nutrition and Physical Activity Among Older Chinese Americans: A Cluster-Randomized Trial. American Journal of Public Health 2016;106(6):1092-8.

*Reason for exclusion*: Wrong patient population

1. Jimenez D.E., Begley A., Bartels S.J., Alegria M., Thomas S.B., Quinn S.C., Reynolds C.F. Improving health-related quality of life in older African American and non-Latino White patients. The American Journal of Geriatric Psychiatry: Official Journal of the American Association for Geriatric Psychiatry 2015;23(6):548-58.

*Reason for exclusion*: Wrong intervention

1. Johnson M.A. Strategies to improve diet in older adults. The Proceedings of the Nutrition Society 2013;72(1):166-72.

*Reason for exclusion*: Wrong study design

1. Johnson S., McLeod B., Gupta S., McLeod K. Impact of a home-based nutrition and exercise intervention in improving functional capacity associated with falls among rural seniors in Canada. Quality in Ageing & Older Adults 2018;19(4):261-272.

*Reason for exclusion*: Wrong study design

1. Kalache A., de Hoogh A.I., Howlett S.E., Kennedy B., Eggersdorfer M., Marsman D.S., Shao A., Griffiths J.C. Nutrition interventions for healthy ageing across the lifespan: a conference report. European Journal of Nutrition 2019;58:1-11.

*Reason for exclusion*: Wrong study design

1. Kamp B.J., Wellman N.S., Russell C. Position of the American Dietetic Association, American Society for Nutrition, and Society for Nutrition Education: Food and nutrition programs for community-residing older adults. Journal of the American Dietetic Association 2010;110(3):463-72.

*Reason for exclusion*: Wrong study design

1. Kennelly S., Kennedy N.P., Corish C.A., Flanagan-Rughoobur G., Glennon-Slattery C., Sugrue S. Sustained benefits of a community dietetics intervention designed to improve oral nutritional supplement prescribing practices. Journal of Human Nutrition & Dietetics 2011;24(5):496-504.

*Reason for exclusion*: Wrong patient population

1. King A.C., Castro C.M., Buman M.P., Hekler E.B., Urizar G.G.J., Ahn D.K. Behavioral impacts of sequentially versus simultaneously delivered dietary plus physical activity interventions: the CALM trial. Annals of Behavioral Medicine: A Publication of the Society of Behavioral Medicine 2013;46(2):157-68.

*Reason for exclusion*: Wrong intervention

1. Klusmann V., Sproesser G., Wolff J.K., Renner B. Positive Self-perceptions of Aging Promote Healthy Eating Behavior Across the Life Span via Social-Cognitive Processes. Journals of Gerontology Series B: Psychological Sciences & Social Sciences 2019;74(5):735-744.

*Reason for exclusion*: Wrong patient population

1. Kogan A.C., Gonzalez J., Hart B., Halloran S., Thomason B., Levine M., Enguidanos S. Be Well: results of a nutrition, exercise, and weight management intervention among at-risk older adults. Journal of Applied Gerontology: The Official Journal of the Southern Gerontological Society 2013;32(7):889-901.

*Reason for exclusion*: Wrong intervention

1. Kosa K.M., Cates S.C., Godwin S.L., Ball M., Harrison R.E. Effectiveness of educational interventions to improve food safety practices among older adults. Journal of Nutrition in Gerontology and Geriatrics 2011;30(4):369-83.

*Reason for exclusion*: Wrong intervention

1. Koutoukidis D.A., Jebb S.A., Ordonez-Mena J.M., Noreik M., Tsiountsioura M., Kennedy S., Payne-Riches S., Aveyard P., Piernas C. Prominent positioning and food swaps are effective interventions to reduce the saturated fat content of the shopping basket in an experimental online supermarket: a randomized controlled trial. The International Journal of Behavioral Nutrition and Physical Activity 2019;16(1):50.

*Reason for exclusion*: Wrong patient population

1. Kwon S.H. Wheel of Wellness Counseling in Community Dwelling, Korean Elders: A Randomized, Controlled Trial. Journal of Korean Academy of Nursing 2015;45(3):459-68.

*Reason for exclusion*: Wrong intervention

1. Lammes E., Rydwik E., Akner G. Effects of nutritional intervention and physical training on energy intake, resting metabolic rate and body composition in frail elderly. a randomised, controlled pilot study. The Journal of Nutrition, Health & Aging 2012;16(2):162-7.

*Reason for exclusion*: Wrong intervention

1. Lando A.M., Lo S.C. Single-larger-portion-size and dual-column nutrition labeling may help consumers make more healthful food choices. Journal of the Academy of Nutrition and Dietetics 2013;113(2):241-50.

*Reason for exclusion*: Wrong patient population

1. Landry A.S., Thomson J.L., Huye H.F., Yadrick K., Connell C.L. Mississippi Communities for Healthy Living: Results of a 6-Month Nutrition Education Comparative Effectiveness Trial. Health Education & Behavior 2017;44(2):316-325.

*Reason for exclusion*: Wrong patient population

1. Lee A.H., Jancey J., Howat P., Burke L., Kerr D.A., Shilton T. Effectiveness of a Home-Based Postal and Telephone Physical Activity and Nutrition Pilot Program for Seniors. Journal of Obesity 2011;786827:1-8.

*Reason for exclusion*: Wrong intervention

1. Lee E.J. Ko L.K. Feng A. Tang C. Lee C. Healthy eating healthy aging: A community-based intervention program for older adult Asian American, native Hawaiian and pacific islanders. Circulation 2017;136(1):A14984.

*Reason for exclusion*: Conference abstract only

1. Lee E.O., Yoon H., Lee J., Yoon J., Chang E. Body-mind-spirit practice for healthy aging. Educational Gerontology 2012;38(7):473-485.

*Reason for exclusion*: Wrong intervention

1. Lee J.S., Johnson M.A., Brown A. Older Americans Act Nutrition Program Improves Participants' Food Security in Georgia. Journal of Nutrition in Gerontology and Geriatrics 2011;30(2):122-139.

*Reason for exclusion*: Wrong intervention

1. Lee J.S., Shannon J., Brown A. Characteristics of Older Georgians Receiving Older Americans Act Nutrition Program Services and Other Home- and Community-Based Services: Findings from the Georgia Aging Information Management System (GA AIMS). Journal of Nutrition in Gerontology and Geriatrics 2015;34(2):168-88.

*Reason for exclusion*: Wrong study design

1. Lee J.S., Sinnett S., Bengle R., Johnson M.A., Brown A. Unmet needs for the Older Americans Act nutrition program. Journal of Applied Gerontology 2011;30(5):587-606.

*Reason for exclusion*: Wrong study design

1. Lee S.Y. The effects of a community-based education, counseling, and exercise camp program on health behaviors in patients with metabolic syndrome. Obesity Facts 2018;11(1):297.

*Reason for exclusion*: Wrong patient population

1. Lee T. Community-based home healthcare project for korean older adults. Osong Public Health and Research Perspectives 2013;4(5):233-9.

*Reason for exclusion*: Wrong intervention

1. Leong J., Jang S.H., Bishop S.K., Brown E.V.R., Lee E.J., Ko L.K. "We understand our community": implementation of the Healthy Eating Healthy Aging program among community-based organizations. Translational Behavioral Medicine 2021;11(2):462-9.

*Reason for exclusion*: Wrong patient population

1. Levin S.M., Ferdowsian H.R., Hoover V.J., Green A.A., Barnard N.D. A worksite programme significantly alters nutrient intakes. Public Health Nutrition 2010;13(10):1629-35.

*Reason for exclusion*: Wrong patient population

1. Lidard M. Cooking up some fun: intergenerational cooking series teaches healthy eating. Johns Hopkins Nursing Summer 2010;8(2):10.

*Reason for exclusion*: Wrong study design

1. Lindegaard Pedersen J. Early nutritional follow-up after discharge prevents deterioration of adl functions in malnourished, independent, geriatric patients who live alone - A randomized clinical trial...T. Suzuki T, Maeda K, Wakabayashi H. Effect of Early Nutritional Follow-Up. Journal of Nutrition, Health & Aging 05 2016;20(5):584.

*Reason for exclusion*: Wrong study design

1. Lloyd J.L., Wellman N.S. Older Americans Act Nutrition Programs: A Community-Based Nutrition Program Helping Older Adults Remain at Home. Journal of Nutrition in Gerontology and Geriatrics 2015;34(2):90-109.

*Reason for exclusion*: Wrong study design

1. Locher J.L., Vickers K.S., Buys D.R., Ellis A., Lawrence J.C., Newton L.E., Roth D.L., Ritchie C.S., Bales C.W. A randomized controlled trial of a theoretically-based behavioral nutrition intervention for community elders: lessons learned from the Behavioral Nutrition Intervention for Community Elders Study. Journal of the Academy of Nutrition and Dietetics 2013;113(12):1675-82.

*Reason for exclusion*: Wrong patient population

1. Logmangino K. Improving nutrition of at-risk-older adults in the community. Clinical Nutrition Insight 2012;38(10):8-9.

*Reason for exclusion*: Wrong study design

1. Loh D.A., Hairi N.N., Choo W.Y., Mohd Hairi F., Peramalah D., Kandiben S., Lee P.L., Gani N., Madzlan M.F., Abd H., Mohd A.I., Akram Z., Chu A.S., Bulgiba A., Cumming R.G. MultiComponent Exercise and theRApeutic lifeStyle (CERgAS) intervention to improve physical performance and maintain independent living among urban poor older people--a cluster randomised controlled trial. BMC Geriatrics 2015;15(1):1-9.

*Reason for exclusion*: Wrong intervention

1. Lorefält B., Wilhelmsson S. A multifaceted intervention model can give a lasting improvement of older peoples’ nutritional status. The Journal of Nutrition Health and Aging 2012;16(4):378-382.

*Reason for exclusion*: Wrong patient population

1. Luger E., Dorner T.E., Haider S., Kapan A., Lackinger C., Schindler K. Effects of a Home-Based and Volunteer-Administered Physical Training, Nutritional, and Social Support Program on Malnutrition and Frailty in Older Persons: A Randomized Controlled Trial. Journal of the American Medical Directors Association 2016;17(7):671.e9-671.e16.

*Reason for exclusion*: Wrong patient population

1. Luten K.A., Dijkstra A., de Winter A.F., Reijneveld S.A. Developing a community-based intervention for Dutch older adults in a socioeconomically disadvantaged community. Health Promotion International 2019;34(3):567-580.

*Reason for exclusion*: Wrong study design

1. Ma G.X., Zhu L., Shive S.E., Zhang G., Senter Y.R., Topete P., Seals B., Zhai S., Wang M., Tan Y. The Evaluation of IDEAL-REACH Program to Improve Nutrition among Asian American Community Members in the Philadelphia Metropolitan Area. International Journal of Environmental Research and Public Health 2019;16(17):3054.

*Reason for exclusion*: Wrong patient population

1. Mabli J., Ohls J. Supplemental nutrition assistance program participation is associated with an increase in household food security in a national evaluation. Journal of Nutrition 2015;145(2):344-351.

*Reason for exclusion*: Wrong patient population

1. Madigan S.M., Fleming P., Wright M.E., Stevenson M., Macauley D. A cluster randomised controlled trial of a nutrition education intervention in the community. Journal of Human Nutrition and Dietetics: The Official Journal of the British Dietetic 2014;27(2):12-20.

*Reason for exclusion*: Wrong patient population

1. Madigan S.M., Fleming P., Wright M.E., Stevenson M., Macauley D. A cluster randomised controlled trial of a nutrition education intervention in the community. Journal of Human Nutrition and Dietetics: The Official Journal of the British Dietetic 2014;27(2):12-20.

*Reason for exclusion*: Wrong patient population

1. Malekafzali H., Eftekhari M.B., Hejazi F., Khojasteh T., Noot R. The effectiveness of educational intervention in the health promotion in elderly people. Iranian Journal of Public Health 06 2010;39(2):18-23.

*Reason for exclusion*: Wrong intervention

1. ManafÒ E., Wong S. Promoting eHealth Literacy in Older Adults: Key Informant Perspectives. Canadian Journal of Dietetic Practice & Research Spring 2013;74(1):37-41.

*Reason for exclusion*: Wrong study design

1. Manilla B., Keller H.H., Hedley M.R. Food tasting as nutrition education for older adults. Canadian Journal of Dietetic Practice and Research 2010;71(2):99-102.

*Reason for exclusion*: Wrong study design

1. Manta C., Alchoufete T., Dodd J. Efficacy of Older Adult Community Nutrition Education...2017 Food & Nutrition Conference & Expo, 2017, Chicago, IL, 21–24 October 2017. Journal of the Academy of Nutrition & Dietetics Supplement 2017;117(9):A44-A44.

*Reason for exclusion*: Wrong conference abstract only

1. Markle-Reid M., Browne G., Gafni A. Nurse-led health promotion interventions improve quality of life in frail older home care clients: Lessons learned from three randomized trials in Ontario, Canada. Journal of Evaluation in Clinical Practice 2013;19(1):118-131.

*Reason for exclusion*: Wrong intervention

1. Marshall S., Agarwal E., Young A., Isenring E. Role of domiciliary and family carers in individualised nutrition support for older adults living in the community. Maturitas 2017;98:20-29.

*Reason for exclusion*: Wrong study design

1. Martin P., Consales J., Scheromm P., Marchand P., Ghestem F., Darmon N. Community gardening in poor neighborhoods in France: A way to re-think food practices? Appetite 2017;116:589-598.

*Reason for exclusion*: Wrong patient population

1. Masters R. Tackling undernutrition in older people from a regional perspective. British Journal of Community Nursing 2010;15(6):266-270.

*Reason for exclusion*: Wrong study design

1. Maximova K., Hanusaik N., Kishchuk N., Paradis G., O'Loughlin J.L. Public health strategies promoting physical activity and healthy eating in Canada: are we changing paradigms?. International Journal of Public Health 2016;61(5):565-72.

*Reason for exclusion*: Wrong study design

1. Mayer R. Dining by design. Advance for Physical Therapy & Rehab Medicine 21(21):12-14.

*Reason for exclusion*: Wrong study design

1. McCarthy S. Weekly patterns, diet quality and energy balance. Physiology & Behavior 2014;134:55-9.

*Reason for exclusion*: Wrong study design

1. McElwaine K.M., Freund M., Campbell E.M., Knight J., Bowman J.A., Doherty E.L., Wye P.M., Wolfenden L., Lecathelinais C., McLachlan S., Wiggers J.H. The delivery of preventive care to clients of community health services. BMC Health Services Research 2013;13(1):1-11.

*Reason for exclusion*: Wrong study design

1. Meengs J.S., Roe L.S., Rolls B.J. Vegetable Variety: An Effective Strategy to Increase Vegetable Intake in Adults. Journal of the Academy of Nutrition and Dietetics 2012;112(8):1211-1215.

*Reason for exclusion*: Wrong patient population

1. Mendonca R., Lopes A.C.S. The effects of health interventions on dietary habits and physical measurements. Revista da Escola de Enfermagem da U S P 2012;46(3):573-9.

*Reason for exclusion*: Wrong patient population

1. Mikkelsen B.E., Novotny R., Gittelsohn J. Multi-Level, Multi-Component Approaches to Community Based Interventions for Healthy Living-A Three Case Comparison. International Journal of Environmental Research and Public Health 2016;13(10):1023.

*Reason for exclusion*: Wrong patient population

1. Molitor F., Sugerman S., Yu H., Biehl M., Aydin M., Levy M., Ponce N.A. Reach of Supplemental Nutrition Assistance Program-Education (SNAP-Ed) interventions and nutrition and physical activity-related outcomes, California, 2011-2012. Preventing Chronic Disease 2015;12:E33.

*Reason for exclusion*: Wrong patient population

1. Morgan G. Health promotion for older people in Wales: Preliminary evaluation of the national service framework for older people. International Journal of Health Promotion and Education 2012;50(1):45-49.

*Reason for exclusion*: Wrong intervention

1. Mountford C.G., Thompson N.P., Hart K. Can a community-based nutritional screening and intervention programme improve clinical outcomes in Newcastle elderly care home residents? Proceedings of the Nutrition Society 2013;72(OCE4):E250.

*Reason for exclusion:* Wrong setting

1. Mountford C.G., Okonkwo A.C.O., Hart K., Thompson N.P. Managing Malnutrition in Older Persons Residing in Care Homes: Nutritional and Clinical Outcomes Following a Screening and Intervention Program. Journal of Nutrition in Gerontology and Geriatrics 2016;35(1):52-66.

*Reason for exclusion*: Wrong patient population

1. Mouodi S., Hosseini S.R., Ghadimi R., Cumming R.G., Bijani A., Mouodi M., Zahed Pasha Y. Lifestyle Interventions to Promote Healthy Nutrition and Physical Activity in Middle-Age (40-60 Years) Adults: A Randomized Controlled Trial in the North of Iran. Journal of Research in Health Sciences 2019;19(1):e00434.

*Reason for exclusion*: Wrong patient population

1. Munoz-Plaza C.E., Morland K.B., Pierre J.A., Spark A., Filomena S.E., Noyes P. Navigating the Urban Food Environment: Challenges and Resilience of Community-dwelling Older Adults. Journal of Nutrition Education & Behavior 2013;45(4):322-331.

*Reason for exclusion*: Wrong study design

1. Murimi M.W., Kanyi M., Mupfudze T., Amin M.R., Mbogori T., Aldubayan K. Factors Influencing Efficacy of Nutrition Education Interventions: A Systematic Review. Journal of Nutrition Education and Behavior 2017;49(2):142-165.

*Reason for exclusion*: Wrong patient population

1. Myers E.F., Spence L.A., Leslie B., Brauer P.M., Spahn J.M., Snetselaar L. Nutrition and telephone counseling: future implications for dietitians and teledietetics. Topics in Clinical Nutrition 2010;25(2):88-108.

*Reason for exclusion*: Wrong study design

1. Nagai N., Shindo N., Wada A., Izu H., Fujii T., Matsubara K., Wada Y., Sakane N. Effects of Rice Wine Lees on Cognitive Function in Community-Dwelling Physically Active Older Adults: A Pilot Randomized Controlled Trial. Journal of Prevention of Alzheimer's Disease 2020;7(2):95-103.

*Reason for exclusion*: Wrong intervention

1. Neelemaat F., Bosmans J.E., Thijs A., Seidell J.C., van Bokhorst-de S., Marian A.E. Post-Discharge Nutritional Support in Malnourished Elderly Individuals Improves Functional Limitations. Journal of the American Medical Directors Association 2011;12(4):295-301.

*Reason for exclusion*: Wrong intervention

1. Neuenschwander L.M., Abbott A., Mobley A.R. Comparison of a web-based vs in-person nutrition education program for low-income adults. Journal of the Academy of Nutrition and Dietetics 2013;113(1):120-6.

*Reason for exclusion*: Wrong patient population

1. Neville, Charlotte E; McKinley, Michelle C; Draffin, Claire R; Gallagher, Nicola E; Appleton, Katherine M; Young, Ian S; Edgar, J. David; Woodside, Jayne V. Participating in a fruit and vegetable intervention trial improves longer term fruit and vegetable consumption and barriers to fruit and vegetable consumption: A follow-up of the ADIT study. The International Journal of Behavioral Nutrition and Physical Activity 2015;12(1):1-7.

*Reason for exclusion*: Wrong intervention

1. Nykanen I., Rissanen T.H., Sulkava R., Hartikainen S. Effects of individual dietary counseling as part of a comprehensive geriatric assessment (CGA) on nutritional status: a population-based intervention study. The Journal of Nutrition, Health & Aging 2014;18(1):54-8.

*Reason for exclusion*: Wrong intervention

1. O'Dare Wilson K. Community food environments and healthy food access among older adults: A review of the evidence for the Senior Farmers' Market Nutrition Program (SFMNP). Social Work in Health Care 2017;56(4):227-243.

*Reason for exclusion*: Wrong study design

1. O'Hara B.J., Phongsavan P., Eakin E.G., Develin E., Smith J., Greenaway M., Bauman A.E. Effectiveness of Australia's Get Healthy Information and Coaching Service: maintenance of self-reported anthropometric and behavioural changes after program completion. BMC Public Health 2013;13(1):1-4.

*Reason for exclusion*: Wrong patient population

1. Oemichen M., Smith C. Investigation of the Food Choice, Promoters and Barriers to Food Access Issues, and Food Insecurity Among Low-Income, Free-Living Minnesotan Seniors. Journal of Nutrition Education and Behavior 2016;48(6):397-404.

*Reason for exclusion*: Wrong study design

1. Osman M., Thornton K. Traffic light labelling of meals to promote sustainable consumption and healthy eating. Appetite 2019;138:60-71.

*Reason for exclusion*: Wrong patient population

1. Otsuka R., Tange C., Tomida M., Nishita Y., Kato Y., Yuki A., Ando F., Shimokata H., Arai H. Dietary factors associated with the development of physical frailty in community-dwelling older adults. The Journal of Nutrition, Health & Aging 2019;23(1):89-95.

*Reason for exclusion*: Wrong study design

1. Panagiotakos D.B. Lessons derived from studies in the elderly: the role of nutrition education in cardiovascular disease prevention. Hormones 2013;12(3):325-6.

*Reason for exclusion*: Wrong study design

1. Parekh S., King D., Boyle F.M., Vandelanotte C. Randomized controlled trial of a computer-tailored multiple health behaviour intervention in general practice: 12-month follow-up results. The International Journal of Behavioral Nutrition and Physical Activity 2014;11(1):41.

*Reason for exclusion*: Wrong patient population

1. Pettigrew S., Jongenelis M.I., Biagioni N., Pratt I.S., Moore S. Results of a long-term follow-up evaluation of an Australian adult nutrition education program. Asia Pacific Journal of Clinical Nutrition 2018;27(5):1155-1159.

*Reason for exclusion*: Wrong patient population

1. Pettigrew S., Jongenelis M.I., Moore S., Pratt I.S. A comparison of the effectiveness of an adult nutrition education program for Aboriginal and non-Aboriginal Australians. Social Science & Medicine 2015;145:120-4.

*Reason for exclusion*: Wrong patient population

1. Pierce M.B., Hudson K.A., Lora K.R., Havens E.K., Ferris A.M. The Husky Byte Program: Delivering Nutrition Education One Sound Byte at a Time. Journal of Nutrition Education & Behavior 2011;43(2):135-136.

*Reason for exclusion*: Wrong patient population

1. Pivonka E., Seymour J., McKenna J., Baxter S.D., Williams S. Development of the behaviorally focused fruits & Veggies--More Matters public health initiative. Journal of the American Dietetic Association 2011;111(10):1570-7.

*Reason for exclusion*: Wrong patient population

1. Pooler J.A., Morgan R.E., Wong K., Wilkin M.K., Blitstein J.L. Cooking Matters for Adults Improves Food Resource Management Skills and Self-confidence Among Low-Income Participants. Journal of Nutrition Education and Behavior 2017;49(7):545-553.

*Reason for exclusion*: Wrong patient population

1. Poscia A., Milovanovic S., La Milia D.I., Duplaga M., Grysztar M., Landi F., Moscato U., Magnavita N., Collamati A., Ricciardi W. Effectiveness of nutritional interventions addressed to elderly persons: umbrella systematic review with meta-analysis. European Journal of Public Health 2018;28(2):275-283.

*Reason for exclusion*: Wrong study design

1. Ramalho R., Medeiros M., Pires J., Ventura L., Coelho F., Pereira, P. Nutritionist knocks on the door: A community nutrition program for non-institutionalized seniors...40th European Society for Clinical Nutrition and Metabolism Congress, September 1-4, 2018, Madrid, Spain. Clinical Nutrition 2018;37:S249-S249.

*Reason for exclusion*: Conference abstract only

1. Rea J., Avgerinou C., Walters K. 162Can Dietary Education and Support Alone Improve Outcomes for Older People at Risk of Malnutrition in the Community?...66th Annual & Scientific Meeting of the Irish Gerontological Society Transforming Ageing Across Borders at Slieve Russell Hotel, Co. Age & Ageing Supplement 2018;47:v13-v60.

*Reason for exclusion*: Wrong study design

1. Reicks M., Kocher M., Reeder J. Impact of Cooking and Home Food Preparation Interventions Among Adults: A Systematic Review (2011-2016). Journal of Nutrition Education and Behavior 2018;50(2):148-172.

*Reason for exclusion*: Wrong study design

1. Resnick B., Hammersla M., Michael K., Galik E., Klinedinst J., Demehin M. Changing behavior in senior housing residents: Testing of phase I of the PRAISEDD-2 intervention. Applied Nursing Research 2014;27(3):162-169.

*Reason for exclusion*: Wrong intervention

1. Resnick B., Michael K., Griffith K., Klinedinst J., Galik E. The Impact of PRAISEDD on Adherence and Initiation of Heart Health Behaviors in Senior Housing...People Reducing Risk And Improving Strength through Exercise, Diet, and Drug Adherence. Public Health Nursing 2014;31(4):309-316.

*Reason for exclusion*: Wrong study design

1. Robare J.F., Milas N.C., Bayles C.M., Williams K., Newman A.B., Lovalekar M.T., Boudreau R., McTigue K., Albert S.M., Kuller L.H. The key to life nutrition program: results from a community-based dietary sodium reduction trial. Public Health Nutrition 2010;13(5):606-14.

*Reason for exclusion*: Wrong intervention

1. Robinson S.M. Improving nutrition to support healthy ageing: what are the opportunities for intervention? The Proceedings of the Nutrition Society 2018;77(3):257-264.

*Reason for exclusion*: Wrong study design

1. Robroek S.J.W., Polinder S., Bredt F.J., Burdorf A. Cost-effectiveness of a long-term Internet-delivered worksite health promotion programme on physical activity and nutrition: a cluster randomized controlled trial. Health Education Research 2012;27(3):399-410.

*Reason for exclusion*: Wrong patient population

1. Rodgers J.T., Purnell J.Q. Healthcare navigation service in 2-1-1 San Diego: guiding individuals to the care they need. American Journal of Preventive Medicine 2012;43(6):S450-6.

*Reason for exclusion*: Wrong intervention

1. Rose Bell E., Rose M., Roll C., Dupont S. The Good Food Box Pilot Project as a Contribution to Addressing Food Accessibility in the Elderly. Canadian Journal of Dietetic Practice & Research 2014;75(4):191-194.

*Reason for exclusion*: Wrong intervention

1. Ross A.B., Pere-Trepat E., Montoliu I., Martin F., Collino S., Moco S., Godin J., Cleroux M., Guy P.A., Breton I., Bibiloni R., Thorimbert A., Tavazzi I., Tornier L., Bebuis A., Bruce S.J., Beaumont M., Fay L., Kochhar S. A whole-grain-rich diet reduces urinary excretion of markers of protein catabolism and gut microbiota metabolism in healthy men after one week. The Journal of Nutrition 2013;143(6):766-73.

*Reason for exclusion*: Wrong patient population

1. Russell J., Flood V., Rochtchina E., Gopinath B., Allman-Farinelli M., Bauman A., Mitchell P. Adherence to dietary guidelines and 15-year risk of all-cause mortality. The British Journal of Nutrition 2013;109(3):547-55.

*Reason for exclusion*: Wrong study design

1. Russell P., Pinnock-Herring R. Research on diet and nutrition in the older adult population. Home Healthcare Nurse 2010;28(9):568-573.

*Reason for exclusion*: Wrong intervention

1. Sadique Z., Harrison D.A., Bear D.E., Rowan K.M., Grieve R. Effectiveness and cost-effectiveness of early nutritional support via the parenteral versus the enteral route for critically ill adult patients. Journal of Critical Care 2019;52:237-241.

*Reason for exclusion*: Wrong study design

1. Saeed A., Fisher J., Mitchell-Smith Z., Brown L.J.E. "You've Got to Be Old to Go There": Psychosocial Barriers and Facilitators to Social Eating in Older Adults. The Gerontologist 2020;60(4):628.

*Reason for exclusion*: Wrong study design

1. Saffel-Shrier S., Johnson M.A., Francis S.L. Position of the Academy of Nutrition and Dietetics and the Society for Nutrition Education and Behavior: Food and Nutrition Programs for Community-Residing Older Adults. Journal of the Academy of Nutrition and Dietetics 2019;119(7):1188-1204.

*Reason for exclusion*: Wrong study design

1. Sakurai R., Yasunaga M., Saito K., Fukaya T., Kim M.-J., Tsunoda N., Muraki E., Suzuki H., Shinkai S., Watanabe S., Fujiwara Y. Effects of a comprehensive intervention program, including hot bathing, on physical function in community-dwelling healthy older adults: A pilot randomized controlled trial. Aging Clinical and Experimental Research 2013;25(4):453-461.

*Reason for exclusion*: Wrong intervention

1. Scarinci I.C., Moore A., Wynn-Wallace T., Cherrington A., Fouad M., Li Y. A community-based, culturally relevant intervention to promote healthy eating and physical activity among middle-aged African American women in rural Alabama: findings from a group randomized controlled trial. Preventive Medicine 2014;69:13-20.

*Reason for exclusion*: Wrong patient population

1. Schifferdecker K.E., Adachi-Mejia A.M., Butcher R.L., O'Connor S., Li Z., Bazos D.A. Translation of an Action Learning Collaborative Model Into a Community-Based Intervention to Promote Physical Activity and Healthy Eating. Health Promotion Practice 2016;17(1):70-9.

*Reason for exclusion*: Wrong patient population

1. Schwingel A., Galvez P. Divine Interventions: Faith-Based Approaches to Health Promotion Programs for Latinos. Journal of Religion and Health 2016;55(6):1891-906.

*Reason for exclusion*: Wrong study design

1. Seah S.J., Brown L.J., Bryant C. Efficacy and challenges of a culturally relevant intervention to improve attitudes to aging. Women's Health 2019;15:1-12.

*Reason for exclusion*: Wrong intervention

1. Seino S., Nishi M., Murayama H., Narita M., Yokoyama Y., Nofuji Y., Taniguchi Y., Amano H., Kitamura A., Shinkai S. Effects of a multifactorial intervention comprising resistance exercise, nutritional and psychosocial programs on frailty and functional health in community-dwelling older adults: A randomized, controlled, cross-over trial. Geriatrics & Gerontology International 2017;17(11):2034-2045.

*Reason for exclusion*: Wrong patient population

1. Seo S., Kim O.Y., Ahn J. Healthy Eating Exploratory Program for the Elderly: Low Salt Intake in Congregate Meal Service. The Journal of Nutrition, Health & Aging 2016;20(3):316-24.

*Reason for exclusion*: Wrong intervention

1. Sheats J.L., Winter S.J., Romero P.P., King A.C. FEAST: Empowering Community Residents to Use Technology to Assess and Advocate for Healthy Food Environments. Journal of Urban Health 2017;94(2):180-189.

*Reason for exclusion*: Wrong study design

1. Sheppard Christine L; Dube, Lise; Ducak, Kate; Myers, Anita M. Development and Evaluation of Let's Do Lunch: A Congregate Meal Program at an Urban Senior Center. Journal of Nutrition in Gerontology and Geriatrics 2018;37(2):49-58.

*Reason for exclusion*: Wrong study design

1. Simoes M., Dumith S.C., Goncalves C.V. Adults and the elderly who received nutritional counseling in a city of southern Brazil: a population-based study. Brazilian Journal of Epidemiology 2019;22(100954576):e190060.

*Reason for exclusion*: Wrong patient population

1. Smith M.L., Bergeron C.D., Lachenmayr S., Eagle L.A., Simon J.R. A Brief Intervention for Malnutrition among Older Adults: Stepping Up Your Nutrition. International Journal of Environmental Research and Public Health 2020;17(10):3590.

*Reason for exclusion*: Wrong study design

1. Smith R. Nutrition standard for community meals to help tackle malnutrition in older people. British Journal of Community Nursing 2010;15(Sup7):S22.

*Reason for exclusion*: Wrong study design

1. Song H., Simon J.R., Patel D.U. Food preferences of older adults in senior nutrition programs. Journal of Nutrition in Gerontology and Geriatrics 2014;33(1):55-67.

*Reason for exclusion*: Wrong intervention

1. Springvloet L., Lechner L., Candel M.J.J.M., de Vries H., Oenema A. Exploring individual cognitions, self-regulation skills, and environmental-level factors as mediating variables of two versions of a Web-based computer-tailored nutrition education intervention aimed at adults: A randomized controlled trial. Appetite 2016;98:101-14.

*Reason for exclusion*: Wrong patient population

1. Springvloet L., Lechner L., de Vries H., Candel M.J.J.M., Oenema A. Short- and medium-term efficacy of a Web-based computer-tailored nutrition education intervention for adults including cognitive and environmental feedback: randomized controlled trial. Journal of Medical Internet Research 2015;17(1):e23.

*Reason for exclusion*: Wrong patient population

1. Springvloet L., Lechner L., de Vries H., Oenema A. Long-term efficacy of a Web-based computer-tailored nutrition education intervention for adults including cognitive and environmental feedback: a randomized controlled trial. BMC Public Health 2015;15(1):1-16.

*Reason for exclusion*: Wrong patient population

1. Stewart D.W., Gabriele J.M., Fisher E.B. Directive support, nondirective support, and health behaviors in a community sample. Journal of Behavioral Medicine 2012;35(5):492-9.

*Reason for exclusion*: Wrong patient population

1. Stoutenberg M., Falcon A., Arheart K., Stasi S., Portacio F., Stepanenko B., Lan M.L., Castruccio-Prince C., Nackenson J. Implementation of Lifestyle Modification Program Focusing on Physical Activity and Dietary Habits in a Large Group, Community-Based Setting. Health Education & Behavior: The Official Publication of the Society for Public Health Education 2017;44(3):421-430.

*Reason for exclusion*: Wrong intervention

1. Stroebele-Benschop N., Depa J., de Castro J.M. Environmental Strategies to Promote Food Intake in Older Adults: A Narrative Review. Journal of Nutrition in Gerontology and Geriatrics 2016;35(2):95-112.

*Reason for exclusion*: Wrong intervention

1. Sutliffe J.T., Fuhrman J.H., Carnot M.J., Beetham R.M., Peddy M.S. Nutrient-dense, Plant-rich Dietary Intervention Effective at Reducing Cardiovascular Disease Risk Factors for Worksites: A Pilot Study. Alternative Therapies in Health and Medicine 2016;22(5):32-36.

*Reason for exclusion*: Wrong patient population

1. Swan J.H., Severance J.J., Turner K. Senior Centers and Nutritional Outcomes: A Texas Example. Social Work in Public Health 2016;31(5):439-52.

*Reason for exclusion*: Wrong study design

1. Sylvie A.K., Jiang Q., Cohen N. Identification of environmental supports for healthy eating in older adults. Journal of Nutrition in Gerontology and Geriatrics 2013;32(2):161-74.

*Reason for exclusion*: Wrong intervention

1. Teixeira P.D.S., Reis B.Z., Vieira D.A.S., da Costa D., Costa J.O., Raposo O.F.F., Wartha E.R.S.A., Netto R.S.M. Educational nutritional intervention as an effective tool for changing eating habits and body weight among those who practice physical activities. Ciencia e Saude Coletiva 2013;18(2):347-356.

*Reason for exclusion*: Wrong language

1. Thomas K.S., Akobundu U., Dosa D. More than a meal? A randomized control trial comparing the effects of home-delivered meals programs on participants' feelings of loneliness. The Journals of Gerontology: Series B: Psychological Sciences and Social Sciences 2016;71(6):1049-1058.

*Reason for exclusion*: Wrong setting

1. Thomson, J.L., Zoellner J.M., Tussing-Humphreys L.M., Goodman M.H. Moderators of intervention dose effects on diet quality and physical activity changes in a church-based, multicomponent, lifestyle study: Delta Body and Soul III. Health Education Research 2016;31(3):339-49.

*Reason for exclusion*: Wrong patient population

1. Thorpe M.G., Milte C.M., Crawford D., McNaughton S.A. Education and lifestyle predict change in dietary patterns and diet quality of adults 55 years and over. Nutrition Journal 2019;18(1):67.

*Reason for exclusion*: Wrong study design

1. Tsofliou F., Grammatikopoulou M.G., Lumley R., Gkiouras K., Lara J., Clark C. Effects of lunch club attendance on the dietary intake of older adults in the UK: A pilot cross-sectional study. Nutrition and health 2020;26(3):209-14.

*Reason for exclusion*: Wrong intervention

1. Tucker C.M., Kang S., Ukonu N.A., Linn G.S., DiSangro C.S., Arthur T.M., Ralston P.A. A Culturally Sensitive Church-Based Health-Smart Intervention for Increasing Health Literacy and Health-Promoting Behaviors among Black Adult Churchgoers. Journal of Health Care for the Poor and Underserved 2019;30(1):80-101.

*Reason for exclusion*: Wrong patient population

1. Tung E.L., Abramsohn E.M., Boyd K., Makelarski J.A., Beiser D.G., Chou C., Huang E.S., Ozik J., Kaligotla C., Lindau S.T. Impact of a Low-Intensity Resource Referral Intervention on Patients' Knowledge, Beliefs, and Use of Community Resources: Results from the CommunityRx Trial. Journal of General Internal Medicine 2020;35(3):815-823.

*Reason for exclusion*: Wrong intervention

1. Turconi G., Rossi M., Roggi C., Maccarini L. Nutritional status, dietary habits, nutritional knowledge and self-care assessment in a group of older adults attending community centres in Pavia, Northern Italy. Journal of Human Nutrition & Dietetics 2013;26(1):48-55.

*Reason for exclusion*: Wrong study design

1. Tussing-Humphreys L., Thomson J., Onufrak S. A Church-Based Pilot Study Designed to Improve Dietary Quality for Rural, Lower Mississippi Delta, African American Adults. Journal of Religion & Health 2015;54(2):455-469.

*Reason for exclusion*: Wrong patient population

1. Umoren J., Brasseur K., Yao P., Ozier A.D., Medina C., Sommer B., Maturrano J. Food Pantries Integrating Eating Competence, Interest/Enjoyment in Physical Activity and Self-Efficacy for Pantry Participants. Journal of Nutrition Education & Behavior 2020;52(2):195-198.

*Reason for exclusion*: Wrong patient population

1. van Dongen E.J.I., Haveman-Nies A., Wezenbeek N.L.W., Dorhout B.G., Doets E.L., de Groot L.C.P.G.M. Effect, process, and economic evaluation of a combined resistance exercise and diet intervention (ProMuscle in Practice) for community-dwelling older adults: design and methods of a randomised controlled trial. BMC Public Health 2018;18(1):877.

*Reason for exclusion*: Wrong intervention

1. van Doorn-van Atten M.N., Haveman-Nies A., Pilichowski P., Roca R., de Vries J.H.M., de Groot C.P.G.M. Telemonitoring to improve nutritional status in community-dwelling elderly: design and methods for process and effect evaluation of a non-randomized controlled trial. BMC Geriatrics 2018;18(1):284.

*Reason for exclusion*: Wrong intervention

1. Volkert D., Beck A.M., Cederholm T., Cruz-Jentoft A., Goisser S., Hooper L., Kiesswetter E., Maggio M., Raynaud-Simon A., Sieber C.C., Sobotka L., van Asselt D., Wirth R., Bischoff S.C. ESPEN guideline on clinical nutrition and hydration in geriatrics. Clinical Nutrition 2019;38(1):10-47.

*Reason for exclusion*: Wrong study design

1. von Berens A., Koochek A., Nydahl M., Fielding R.A., Gustafsson T., Kirn D.R., Cederholm T., Sodergren M. "Feeling More Self-Confident, Cheerful and Safe". Experiences from a Health-Promoting Intervention in Community Dwelling Older Adults - A Qualitative Study. The Journal of Nutrition, Health & Aging 2018;22(4):541-548.

*Reason for exclusion*: Wrong study design

1. Wagner, Meredith; Rhee, Y. Community-based Nutrition Education Improves Knowledge, Attitudes, and Behaviors Related to Fruit and Vegetable Consumption. Journal of Nutrition Education & Behavior Supplement 2013;45:S39-S39.

*Reason for exclusion*: Wrong patient population

1. Walker J., Ainsworth B., Hooker S., Keller C., Fleury J., Chisum J., Swan P. Optimal Health (Spirit, Mind, and Body): A Feasibility Study Promoting Well-Being for Health Behavior Change. Journal of Religion and Health 2015;54(5):1681-98.

*Reason for exclusion*: Wrong patient population

1. Walkinshaw L.P., Quinn E.L., Rocha A., Johnson D.B. An Evaluation of Washington State SNAP-Ed Farmers' Market Initiatives and SNAP Participant Behaviors. Journal of Nutrition Education and Behavior 2018;50(6):536-546.

*Reason for exclusion*: Wrong patient population

1. Wallace R., Lo J., Devine A. Tailored Nutrition Education in the Elderly Can Lead to Sustained Dietary Behaviour Change. The Journal of Nutrition, Health & Aging 2016;20(1):8-15.

*Reason for exclusion*: Wrong patient population

1. Walters K., Frost R., Kharicha K., Avgerinou C., Gardner B., Ricciardi F., Hunter R., Liljas A., Manthorpe J., Drennan V., Wood J., Goodman C., Jovicic A., Iliffe S. Home-based health promotion for older people with mild frailty: the HomeHealth intervention development and feasibility RCT. Health technology assessment 2017;21(73):1-128.

*Reason for exclusion*: Wrong intervention

1. Walton K., Pettingill H., Charlton K., McMahon A., North J., Whiting D., Pearson R. Evaluating the nutrition screening program within kiama meals on wheels: Perspectives of the clients and their general practitioners. Revista Espanola de Nutricion Humana y Dietetica 2016;20:366.

*Reason for exclusion*: Wrong study design

1. Wang D., Glicksman A. "Being Grounded": Benefits of Gardening for Older Adults in Low-Income Housing. Journal of Housing for the Elderly 2013;27(1-2):89-104.

*Reason for exclusion*: Wrong study design

1. Waterlander W.E., de Boer M.R., Schuit A.J., Seidell J.C., Steenhuis I.H.M. Price discounts significantly enhance fruit and vegetable purchases when combined with nutrition education: a randomized controlled supermarket trial. The American Journal of Clinical Nutrition 2013;97(4):886-95.

*Reason for exclusion*: Wrong patient population

1. Watkins I., Kules B., Yuan X., Xie B. Heuristic Evaluation of Healthy Eating Apps for Older Adults. Journal of Consumer Health on the Internet 2012;16(4):105-127.

*Reason for exclusion*: Wrong study design

1. Weddle D., Wilson F.L., Berkshire S.D., Heuberger R. Evaluating nutrition risk factors and other determinants of use of an urban congregate meal program by older African Americans. Journal of Nutrition in Gerontology and Geriatrics 2012;31(1):38-58.

*Reason for exclusion*: Wrong study design

1. Wedick N.M., Ma Y., Olendzki B.C., Procter-Gray E., Cheng J., Kan K.J., Ockene I.S., Pagoto S.L., Land T.G., Li W. Access to healthy food stores modifies effect of a dietary intervention. American Journal of Preventive Medicine 2015;48(3):309-17.

*Reason for exclusion*: Wrong patient population

1. Wielan M.L., Weis J.A., Hanza M.M.K., Meiers S.J., Patten C.A., Clark M.M., Sloan J.A., Novotny P.J., Njeru J.W., Abbenyi A., Levine J.A., Goodson M., Porraz C., Maria G.D., Osman A., Hared A., Nigon J.A., Sia I.G. Healthy immigrant families: Participatory development and baseline characteristics of a community-based physical activity and nutrition intervention. Contemporary Clinical Trials 2016;47:22-31.

*Reason for exclusion*: Wrong patient population

1. Wilcox S., Parrott A., Baruth M., Laken M., Condrasky M., Saunders R., Dowda M., Evans R., Addy C., Warren T.Y., Kinnard D., Zimmerma L. The Faith, Activity, and Nutrition program: a randomized controlled trial in African-American churches. American Journal of Preventive Medicine 2013;44(2):122-31.

*Reason for exclusion*: Wrong patient population

1. Wingrove K., Barbour L., Palermo C. Exploring nutrition capacity in Australia's charitable food sector. Nutrition & Dietetics 2017;74(5):495-501.

*Reason for exclusion*: Wrong study design

1. Wolfenden L., Paul C.L., Tzelepis F., Freund M., Wiggers J., Gillham K. Acceptability of proactive telephone recruitment to a telephone support service to encourage healthy eating, physical activity and weight loss. Australian and New Zealand Journal of Public Health 2012;36(3):295-6.

*Reason for exclusion*: Wrong patient population

1. Wong E.Y., Lee A.H., James A.P., Jancey J. Physical activity and nutrition intervention for Singaporean women aged 50 years and above: study protocol for a randomised controlled trial. Trials 2018;19(1):257.

*Reason for exclusion*: Wrong study design

1. Worawong C., Borden M.J., Cooper K.M., Pérez O.A., Lauver D. Evaluation of a Person-Centered, Theory-Based Intervention to Promote Health Behaviors. Nursing Research 2018;67(1):6-15.

*Reason for exclusion*: Wrong patient population

1. Worsley A., Wang W.C., Yeatman H., Byrne S., Wijayaratne P. Does school health and home economics education influence adults' food knowledge? Health Promotion International 2016;31(4):925-935.

*Reason for exclusion*: Wrong patient population

1. Wright L., Vance L., Sudduth C., Epps J.B. The Impact of a Home-Delivered Meal Program on Nutritional Risk, Dietary Intake, Food Security, Loneliness, and Social Well-Being. Journal of Nutrition in Gerontology and Geriatrics 2015;34(2):218-27.

*Reason for exclusion*: Wrong intervention

1. Wu S., Hsu L., Hsu C., Hsieh T., Su S., Peng Y., Guo T., Kang Y., Pan W. Dietary education with customised dishware and food supplements can reduce frailty and improve mental well-being in elderly people: A single-blind randomized controlled study. Asia Pacific Journal of Clinical Nutrition 2018;27(5):1018-1030.

*Reason for exclusion*: Wrong patient population

1. Wunderlich S., Bai Y., Gallop E. Interactive Nutrition Education Improves Nutrition Risk Factors among Older Adults Participating in Government- Sponsored Programs. Journal of the American Dietetic Association 2010;110(9):A118.

*Reason for exclusion*: Wrong study design

1. Wurzer B.M., Hurkmans E.J., Waters D.L. The Use of Peer-Led Community-Based Programs to Promote Healthy Aging. Current Geriatrics Reports 2017;6(3):202-211.

*Reason for exclusion*: Wrong study design

1. Yao J., Johnny J. It's a Matter of Perspective: The Role of Aging Expectations and Self-Efficacy Towards Engagement in Healthy Lifestyles Among Older Adults. Asian Pacific Island Nursing Journal 2019;4(3):116-122.

*Reason for exclusion*: Wrong study design

1. Yin Z., Brasher M.S., Kraus V.B., Lv Y., Shi X., Zeng Y. Dietary Diversity Was Positively Associated with Psychological Resilience among Elders: A Population-Based Study. Nutrients 2019;11(3):650.

*Reason for exclusion*: Wrong study design

1. Young K., Bunn F., Trivedi D., Dickinson A. Nutritional education for community dwelling older people: A systematic review of randomised controlled trials. International Journal of Nursing Studies 2011;48(6):751-780.

*Reason for exclusion*: Wrong study design

1. Zheng X., Zhang C.C. Effectiveness of the SMG model for improving health among empty nesters: A health management model in the community. Journal of the American Geriatrics Society 2019;67(4):S665.

*Reason for exclusion*: Wrong intervention

1. Zhou X., Perez-Cueto F.J.A., Dos Santos Q., Monteleone E., Giboreau A., Appleton K.M., Bjorner T., Bredie W.L.P., Hartwell H. A systematic review of behavioural interventions promoting healthy eating among older people. Nutrients 2018;10(2):128.

*Reason for exclusion*: Wrong study design

1. Zhu H., An R. Impact of home-delivered meal programs on diet and nutrition among older adults: a review. Nutrition and Health 2013;22(2):89-103.

*Reason for exclusion*: Wrong study design
